# Nrf2-mediated redox balance alleviates LPS-induced vascular endothelial cell inflammation by inhibiting endothelial cell ferroptosis

**DOI:** 10.1038/s41598-024-53976-3

**Published:** 2024-02-09

**Authors:** Huimin Hou, Xiujiao Qin, Gaokai Li, Zhitao Cui, Jin Zhang, Bin Dong, Zhicheng Wang, Huiying Zhao

**Affiliations:** 1 Department of Geriatrics, First Hospital of Jilin University, Changchun, 130021 China; 2 Department of Critical Care Medicine, Shandong First Medical University Affiliated Province Hospital, Jinan, 250023 China; 3 School of Life and Health Science, Huzhou College, Huzhou, 313000 China; 4 NHC Key Laboratory of Radiobiology, School of Public Health, Jilin University, Changchun, 130021 China

**Keywords:** Cell biology, Molecular biology, Cardiology

## Abstract

Ferroptosis plays an important role in inflammation and oxidative stress. Whether ferroptosis is involved in the inflammation of vascular endothelial cells and its regulation mechanism remains unclear. We estimated the correlation between serum iron ion levels and the inflammation index of 33 patients with arteriosclerosis. In vitro, HUVECs with or without ferrostatin-1 were exposed to Lipopolysaccharide. Corresponding cell models to verify the target signaling pathway. The results showed that serum iron ion levels had a significant positive correlation with N ratio, N/L, LDL level, and LDL/HDL (*P* < 0.05), and a negative correlation with L ratio (*P* < 0.05) in the arteriosclerosis patients. In vitro, ferroptosis is involved in HUVECs inflammation. Ferrostatin-1 can rescue LPS-induced HUVECs inflammation by decreasing HMGB1/IL-6/TNF-α expression. Nrf2 high expression could protect HUVECs against ferroptosis by activating the GPX4/GSH system, inhibiting ferritinophagy, and alleviating inflammation in HUVECs by inhibiting HMGB1/IL-6/TNF-α expression. It also found that Nrf2 is a key adaptive regulatory factor in the oxidative damage of HUVECs induced by NOX4 activation. These findings indicated that ferroptosis contributed to the pathogenesis of vascular endothelial cell damage by mediating endothelial cell inflammation. Nrf2-mediated redox balance in vascular inflammation may be a therapeutic strategy in vascular diseases.

## Introduction

Vascular endothelial cells are a layer of cells lining the inner surface of blood vessels that act as a barrier to regulate microenvironmental homeostasis^1^. Endothelial cell injury is an important factor inducing many kinds of vascular-related diseases, including cardiovascular disease (CAD), Atherosclerosis (AS), and stroke^2^. Studies suggested that multiple cell death modes are involved in endothelial cell injury, it involves cell apoptosis, necrosis, autophagy, and other regulatory cell death modes^3–5^. At the same time, dying cells induce oxidative stress and inflammation, further exacerbating disease progression^6^.

Ferroptosis is a new type of programmed cell death that depends on intracellular iron ions and lipotoxicity, which is triggered by lipid peroxidation caused by iron-mediated Fenton reaction^7^. Recent studies showed that ferroptosis plays an important role in inflammation, inhibition of ferroptosis can effectively alleviate the progression of inflammation-related diseases^8,9^. Inflammation is a critical risk factor that induces endothelial dysfunction and further mediates the initiation and plaque progression of AS^10^. At present, whether ferroptosis is involved in the inflammation of vascular endothelial cells and its mediating mechanism remains unclear.

Nuclear factor E2 related factor 2 (Nrf2) is an important antioxidant transcription factor that plays key roles in oxidative defense by regulating the expression of various antioxidant factors^11,12^. Studies have found that Nrf2 is also a negative regulator of ferroptosis, which can regulate the expression of several key genes of ferroptosis by activating the glutathione transport system, improving ferritinophagy, modulating mitochondrial activity and lipid peroxidation^13–15^. In addition, Nrf2 is also an important regulator of oxidative homeostasis in oxidative damage induced by the reactive oxygen species (ROS) producing factor NADPH oxidase 4 (NOX4)^16^. However, the roles of NOX4/Nrf2 redox balance in vascular endothelial cell oxidative injury still need to be systematically studied.

In our present study, we make a correlation analysis between serum iron levels and serum inflammation, and lipid metabolism dysfunction in arteriosclerosis patients. In vitro, we established a lipopolysaccharide (LPS)-induced HUVECs inflammation model, investigated whether ferroptosis is involved in the inflammation of vascular endothelial cells, and further explored the roles of the Nrf2 in vascular endothelial cells inflammation and cell survival. The results clearly stated that serum iron level was significantly positively correlated with N ratio, N/L, LDL levels, and LDL/HDL levels. Ferroptosis is involved in endothelial inflammation pathology. Nrf2-mediated redox balance plays an important protective role in ferroptosis-dependent inflammation of vascular endothelial cells.

## Materials and methods

### Clinical samples

The clinical data and serum of 33 patients with arteriosclerosis in the First Hospital of Jilin University have been collected following the Helsinki Declaration. Clinical data collection adheres to the following criteria. Inclusion Criteria: Meet the diagnostic criteria for atherosclerosis^17^. Exclusion Criteria: Individuals with missing clinical case data; individuals with severe liver, kidney, or blood system diseases; individuals with tumors; individuals with autoimmune diseases; individuals who have taken medications affecting iron metabolism in the past 6 months; individuals who have taken iron supplements in the past 6 months; individuals who have had severe infections in the past 6 months. The present study was approved by the Internal Review Board (IRB) of the First Hospital of Jilin University, and each participant signed the informed consent.

### Cell lines

Human umbilical vein endothelial cells (HUVEC) were purchased from Otwo Biotech (Shenzhen, China) Inc, and cultured in DMEM/High glucose (Hyclone, South Logan, UT, USA) supplemented with 10% Fetal bovine serum (Cellmax Lanzhou, China) and 1X Antibiotic–Antimycotic (100 μg/mL penicillin, and 100 μg/mL streptomycin (Hyclone, South Logan, UT, USA) in a 37 °C humidified incubator with 5% CO_2_.

### Reagents and antibodies

Ferrostatin-1 (Fer-1, HY-100579) and ML385 (HY-100523) were purchased from MedChemExpress (Monmouth Junction, NJ, USA). Lipopolysaccharide (LPS, L2880, 055:B5) and N-acetylcysteine(NAC, CAS#38520-57-9) were purchased from Sigma-Aldrich (St. Louis, MO, USA). FTH (DF6278, 1:1000) and xCT (DF12509, 1:1000) antibodies were purchased from Affinity Biosciences (Cincinnati, OH, USA). NOX4 (YN2975, 1:1000), PTGS2 (YT1073, 1:1000), NCOA4 (YT0302, 1:1000), NRF2 (YT3189, 1:1000), GPX4 (YN3047, 1:1000), β-Actin (YM3028, 1:5000), and HRP* Goat Anti Rabbit IgG (H + L) (RS0002, 1:10,000) antibodies were purchased from Immunoway (Newark, DE, USA). IL-6 (WL02841, 1:1000), TNF-α (WL01581, 1:1000), and Histone H3 (WL0984a, 1:500) antibodies were acquired from Wanleobio (Shenyang, China). HMGB1 antibody (T55060, 1:1000) was acquired from Abmart (Shanghai, China). Cy3 Goat Anti-Rabbit IgG (H + L) was purchased from Abclonal (Wuhan, China).

### Cell viability assay

In brief, HUVECs (5 × 10^3^ cells/well) were seeded into 96-well plates, the medium of 100 μl and CCK-8 assay kit (#IV08, Invigentech™, Carlsbad, CA, USA) of 10 μl were added into every well and incubated at 37 °C for 3 h, the absorbance value (A_450_) was measured at 450 nm.

### Intracellular and serum iron, malondialdehyde (MDA), and glutathione (GSH) assay

The levels of intracellular *and serum* iron ions, MDA, and GSH were measured using an Iron detection kit (A039-2-1), Micro-reduced glutathione detection kit (A006-2-1), and Cell MDA Detection Kit (A003-4-1) (Nanjing Jiancheng, Nanjing, China), respectively, according to the manufacturer’s instructions.

### Intracellular ROS assay

Intracellular ROS was measured by the DCFH-DA probes^18^. In brief, HUVECs were treated with indicated manners, then the cells were washed with PBS and stained with 10 μM DCFH-DA (S0033S, Beyotime, Shanghai, China) for 45 min at 37 °C in the dark. After incubation, the cells were washed with PBS three times, and then the cells were observed with a Cytation3 Imaging System (BioTek, Winooski, VT, USA).

### Immunofluorescence

For immunofluorescence analysis of NRF2 protein, in brief, sterile coverslips (22 mm × 22 mm) were placed in six-well plates. HUVECs (2.0 × 10^5^ cells/well) were seeded in these wells and were treated with indicated manners. After that, the cells were washed with PBS three times. Cells were fixed in 4% paraformaldehyde for 15 min, permeability in 0.5% Triton X-100 for 5 min, and blocked in 5% BSA for 1 h. Then the cells were added primary antibody (NRF2, 1:200) and incubated overnight at 4 °C, after that, the cells were added fluorescent secondary antibody (1:400, #AS007, Abclonal, Wuhan, China) for 1 h at room temperature. Cells nuclear were stained with DAPI for 5 min. Finally, cells were observed with a confocal microscope.

### Nrf2 and NOX4 overexpression plasmid transfection

Nrf2 and NOX4 overexpression plasmid were amplified in *Escherichia coli* (*E. coli*) and were extracted using EndoFree Maxi Plasmid Kit (TIANGEN, China). For cell transfection, in brief, HUVECs (2.0 × 10^5^ cells/well) were seeded in six-well plates. When approximately 70–80% confluence was reached, HUVECs were transfected with Nrf2 or NOX4 overexpression plasmid and empty plasmid for 48 h using Sage LipoPlus Transfection Reagent (Beijing Saizhi, China).

### Western blot

Add protease inhibitors and lyse HUVECs cells in cold lysis buffer. Equal amounts of protein samples are separated by SDS-PAGE and transferred onto nitrocellulose membranes. Cut out the corresponding target bands, block the nitrocellulose membrane with 5% skim milk, and incubate the cut target bands with a matching specific primary antibody at 4 °C overnight. Then incubate with a matching secondary antibody for 1 h. Finally, visualize the protein using a chemiluminescence imaging system (Saizhi, Beijing, China). If the protein bands have poor staining, adjust the imaging method to integrate the image.

Nuclear and cytoplasmic proteins of NRF2 were extracted using a Nuclear and Cytoplasmic Protein Extraction Kit (Beyotime, Shanghai, China) following the manufacturer’s protocol.

### RNA extraction and real-time quantitative PCR (RT-qPCR)

The total RNA was isolated using TRIzol reagent (Invitrogen, Carlsbad, CA, USA) and subjected to cDNA synthesis by RT EasyTM II (With gDNase) (Foregene, Chengdu, China) following the manufacturer's instruction. Then, 2 × Realtime PCR Super Mix (Mei5bio, Beijing, China) was used for RT-qPCR. Then the PCR amplification reactions were comprised as the following instruction: 95 °C for 1 min, followed by 40 cycles of 95 °C for 5 s, 60 °C for 10 s, and 72 °C for 15 s manufacturer’s protocol. The PCR primers were as follows: *β-actin*, forward primer sequence: 5′-CAGGTCATCACCATTGGCAATGAGC-3′, reverse primer sequence: 5′-CGGATGTCCACGTCACACTTCATGA-3′; *NRF2*, forward primer sequence: 5′-TCAGCGACGGAAAGAGTATGA-3′, reverse primer sequence: 5′-CCACTGGTTTCTGACTGGATGT-3′; *NOX4*, forward primer sequence: 5′-CCAAGCAGGAGAACCAGGAGATTG-3′, reverse primer sequence: 5′-AGAAGTTGAGGGCATTCACCAGATG-3′.

### Statistical analysis

Statistical analysis was performed by SPSS 24.0 and GraphPad Prism 8. Measurement data conforming to normal distribution were expressed as mean ± standard difference, and the data were calculated by independent sample t-test. Measurement data that did not conform to normal distribution were expressed as median (M) and interquartile range (Q25–Q75). Counting data were expressed as frequency and percentage (%). The Pearson correlation test was used for bivariate linear correlation analysis. *P* < 0.05 was considered statistically significant. All data were generated from three independent experiments.

### Ethical approval

The study was conducted in accordance with the Declaration of Helsinki, and approved by the Institutional Review Board of the First Hospital of Jilin University (2020-413). Informed consent was obtained from all subjects involved in the study.

## Results

### Serum iron levels are associated with inflammation and lipid dysfunction in arteriosclerosis patients

The patients with arteriosclerosis were collected to investigate whether ferroptosis is involved in the progress of vascular injury in arteriosclerosis. The basic clinical data of the patients were analyzed (Table 1).

**Table 1:** Clinical characteristics.

Variables	N = 33
Age(Y)	63.85 ± 6.66
Sex(M)	7 (21.21%)
Hypertension	18 (54.55%)
Diabetes mellitus	7 (21.21%)
Smoking	5 (15.15%)
Alcohol consummption	2 (6.00%)
WBC (*10^9^/L)	5.68 ± 1.35
N%	61.36 ± 8.95
L%	29.24 ± 7.15
N/L	2.21 (1.68,2.61)
LDL (mmol/L)	3.01 ± 0.84
LDL/HDL	2.60 ± 0.76
Serum iron levels (mg/L)	1.20 ± 0.37
Serum GSH levels (umol/L)	7.79 (5.19,13.00)
Serum MDA levels (nmol/L)	2.58 ± 0.82

**Figure 1:**
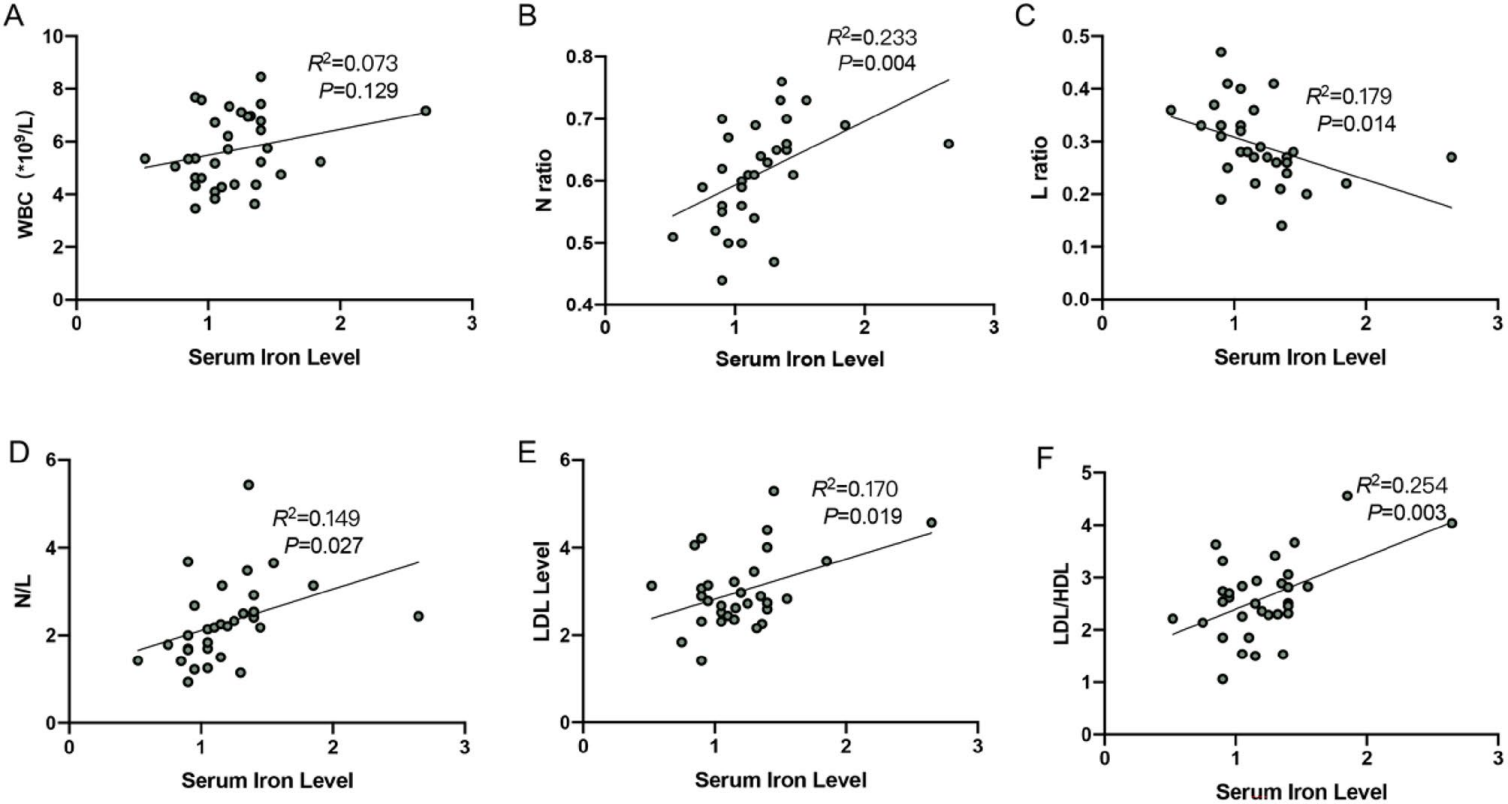
(**A**–**F**) The correlation analyses between serum iron levels and WBC, N ratio, L ratio, N/L, LDL level, and LDL/HDL in arteriosclerosis patients.

Iron overload is related to lipid metabolism disorders, atherosclerotic plaque growth, and instability^19^. The correlation between serum iron ion level and serum inflammation level and serum lipid level was analyzed. The results indicated that there was a significant positive correlation between serum iron levels and neutrophil ratio (N ratio), neutrophil/lymphocyte (N/L), low-density lipoprotein (LDL) level, and low-density lipoprotein/high-density lipoprotein (LDL/HDL) (*P* < 0.05) (Fig. 1B,D–F), a negative correlation between serum iron levels and lymphocyte ratio (L ratio) (*P* < 0.05) (Fig. 1C) in the arteriosclerosis patients, and we also found that with the increase of serum iron ion level, white blood cell (WBC) count also increased (Fig. 1A).

### Ferroptosis is involved in LPS-induced HUVEC inflammation

**Figure 2:**
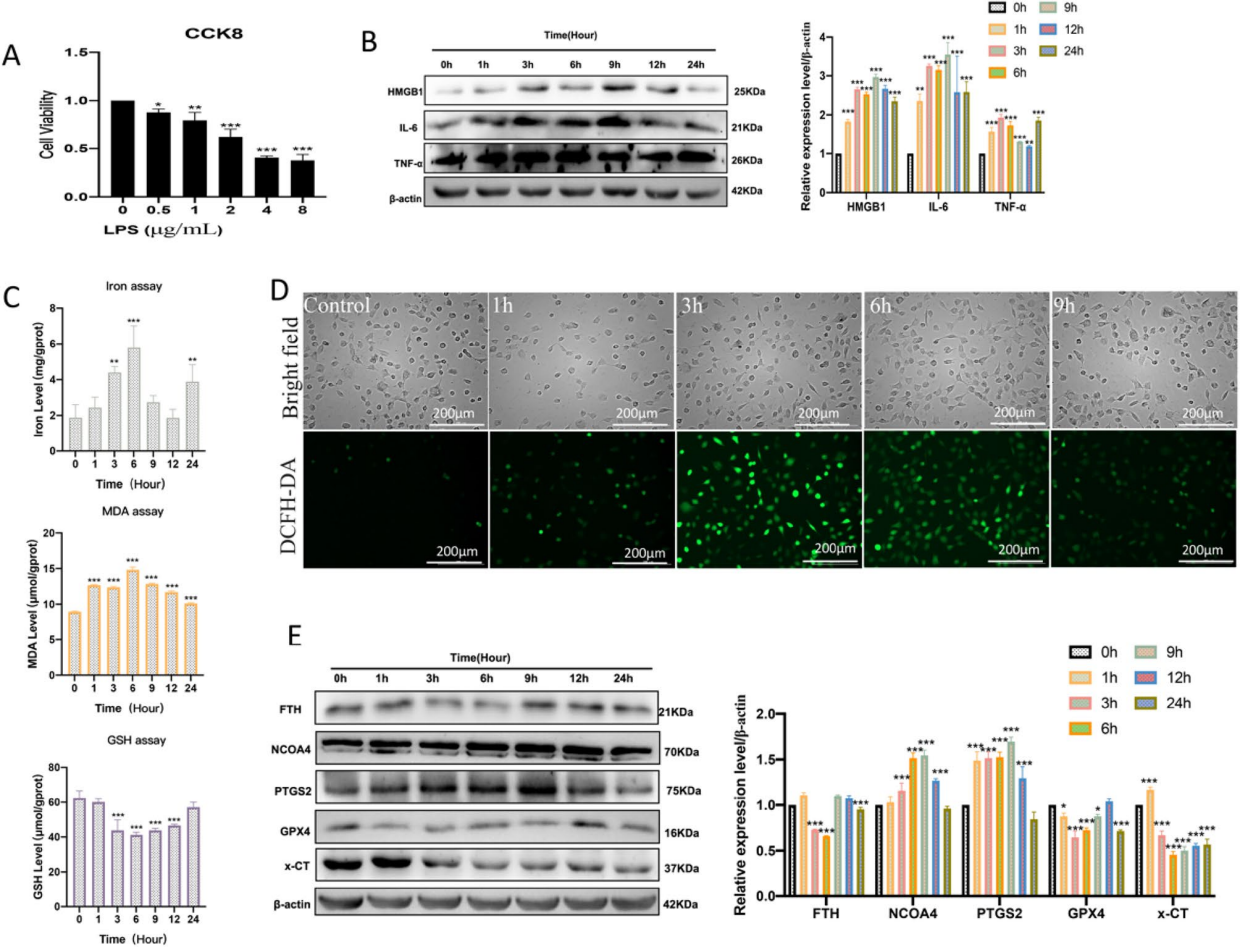
Ferroptosis is involved in LPS-induced HUVEC inflammation. (**A**) The cell viability 24 h after LPS (0, 0.5, 1, 2, 4, and 8 μg/mL) treatment was assayed by CCK-8; *P < 0.05, **P < 0.01, ***P < 0.001 versus 0 μg/mL group. (**B**) Western blot was used to detect the expression levels of inflammation-related proteins; **P < 0.01, ***P < 0.001 versus 0 h group. (**C**) The intracellular iron ion, GSH, and MDA levels were measured after 2 μg/mL LPS treatment at different times (0 h,1 h,3 h,6 h,9 h,12 h,24 h); **P < 0.01, ***P < 0.001 versus the 0 h group. (**D**) The DCFH-DA probes were performed for intracellular ROS levels (scale bar, 200 μm) after 2 μg/mL LPS treatment for different times (0 h,1 h,3 h,6 h,9 h). (**E**) Western blot was used to detect the expression levels of ferroptosis-related proteins after 2 μg/mL LPS treatment for different times (0 h,1 h,3 h,6 h,9 h,12 h,24 h); *P < 0.05, ***P < 0.001 versus 0 h group.

To further explore whether ferroptosis is involved in vascular endothelial inflammation. The LPS was used to induce vascular endothelial cell inflammation in vitro. The CCK8 assay showed that LPS treatment obviously inhibited the cell activity of HUVECs in a dose-dependent manner (Fig. 2A). HUVECs displayed a significant decrease in cell proliferation activity in a dose of 2 μg/mL LPS, so we chose 2 μg/mL LPS for subsequent cellular experiments. The results detected that inflammation-related protein expression levels of HMGB1, IL-6, and TNF-α were increased in a time-dependent manner in the presence of LPS (Fig. 2B). Next, we performed ferroptosis detection in LPS-induced HUVECs, the results indicated that the contents of intracellular iron ions, GSH depletion, and MDA levels were increased after LPS treatment (Fig. 2C), and fluorescent images showed that the ROS levels were aggravated following treatment with LPS (Fig. 2D). In addition, the western blot analysis results showed that LPS treatment decreased lipid peroxidation-related proteins GPX4 and x-CT levels, increased PTGS2 levels, it also indicated that LPS treatment increased ferritinophagy-ralated proteins NCOA4 levels, decreased FTH levels (Fig. 2E). Therefore, these findings suggested that ferroptosis was involved in LPS-induced HUVECs inflammation.

### LPS-induced ferroptosis and inflammation could be rescued by ferrostatin-1

**Figure 3:**
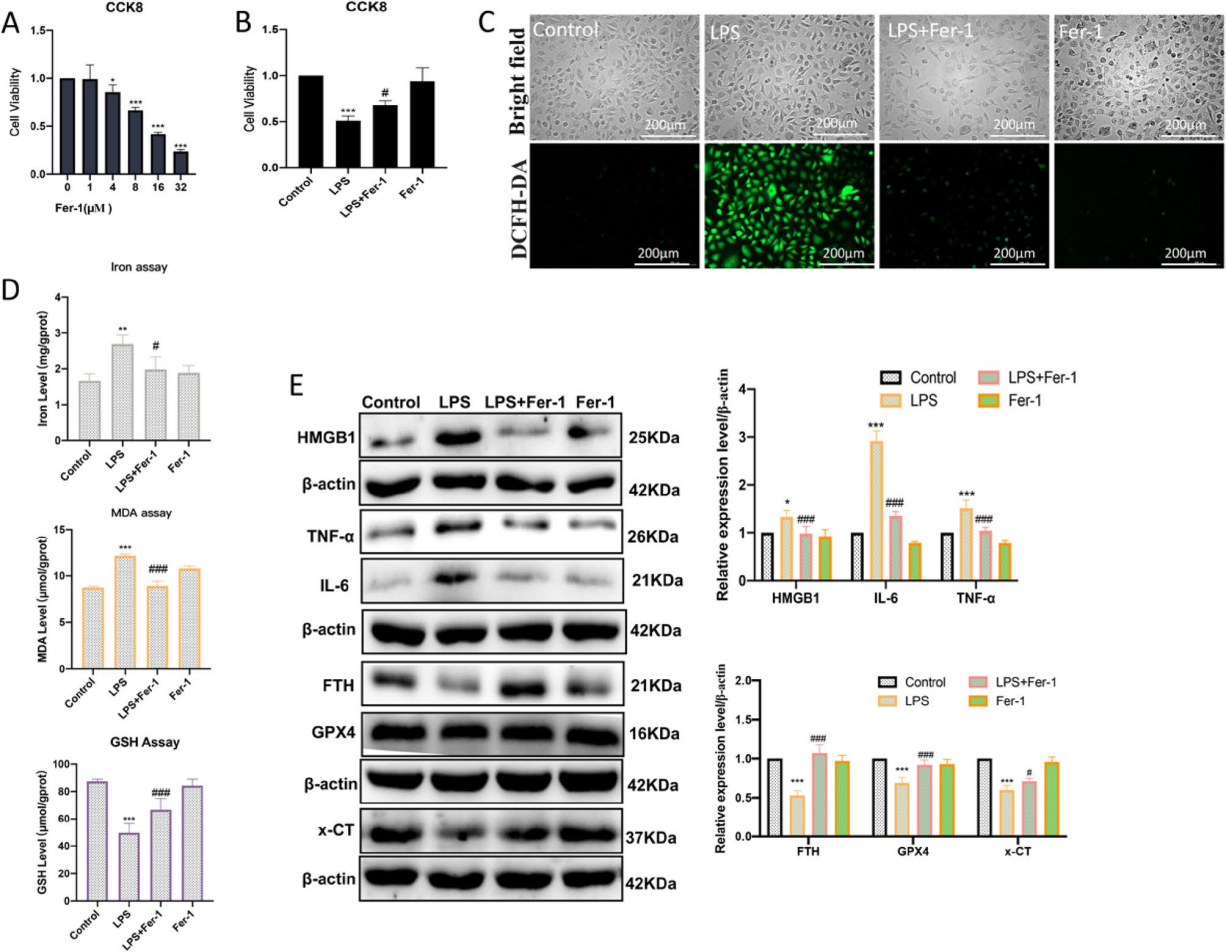
LPS-induced ferroptosis and inflammation could be rescued by ferrostatin-1. (**A**) The cell viability 24 h after Fer-1 (0, 1, 4, 8, 16, and 32 μM) treatment was assayed by CCK-8; *P < 0.05, ***P < 0.001 versus 0 μM group. (**B**) Fer-1(1 μM) pretreated for 3 h significantly ameliorated LPS (24 h)-induced death of HUVECs; ***P < 0.001 versus Control group; ^#^*p* < 0.05 versus LPS group. (**C**) The DCFH-DA probes were performed for LPS (3 h)-induced ROS levels (scale bar, 200 μm) after Fer-1 (1 μM) pretreated for 3 h. (**D**) The LPS (6 h)-induced iron ion, GSH, and MDA levels were measured after Fer-1 (1 μM) pretreated for 3 h; **P < 0.01, ***P < 0.001 versus Control group, ^#^*P* < 0.05, ^###^*P* < 0.001 versus LPS group. (**E**) The LPS (6 h)-induced ferroptosis and inflammation-related protein expression levels were measured by western blot after Fer-1(1 μM) pretreated for 3 h; *P < 0.05, ***P < 0.001 versus Control group, ^#^*P* < 0.05, ^###^*P* < 0.001 versus LPS group.

To further verify that LPS-induced inflammation in HUVECs is ferroptosis dependent, the HUVECs were pretreated with ferroptosis inhibitor Ferrostatin-1 (Fer-1). 1 μM Fer-1 did not affect cell viability, so we chose 1 μM Fer-1 for subsequent cellular experiments (Fig. 3A). The ferroptosis of HUVECs treated with LPS with or without Fer-1 cotreatment was detected. The CCK8 assay showed that LPS treatment obviously inhibited the proliferation activity of HUVECs, while Fer-1 pretreated significantly ameliorated the LPS-induced death of HUVECs (Fig. 3B). As expected, fluorescent images showed that the ROS levels were aggravated following treatment with LPS, while the change can be attenuated by Fer-1 (Fig. 3C). The contents of intracellular iron ions, MDA levels, and GSH depletion were increased after LPS treatment, while these elevations were remarkably curbed after Fer-1 pretreatment (Fig. 3D). In addition, we also measured the ferroptosis and inflammation-related protein expression levels. The western blot results showed that LPS treatment decreased the proteins GPX4, x-CT, and FTH, and increased PTGS2, NCOA4, HMGB1, IL-6, and TNF-α (Fig. 3E). However, Fer-1 pretreated alleviated the decreased expression of GPX4, x-CT, and FTH, and restored the expression of PTGS2, NCOA4, HMGB1, IL-6, and TNF-α (Fig. 3E). All these findings suggested that ferroptosis was an important form induced by LPS in endothelial cell inflammation.

### Nrf2 plays a protective role in LPS-induced HUVEC inflammation by inhibiting ferroptosis

**Figure 4:**
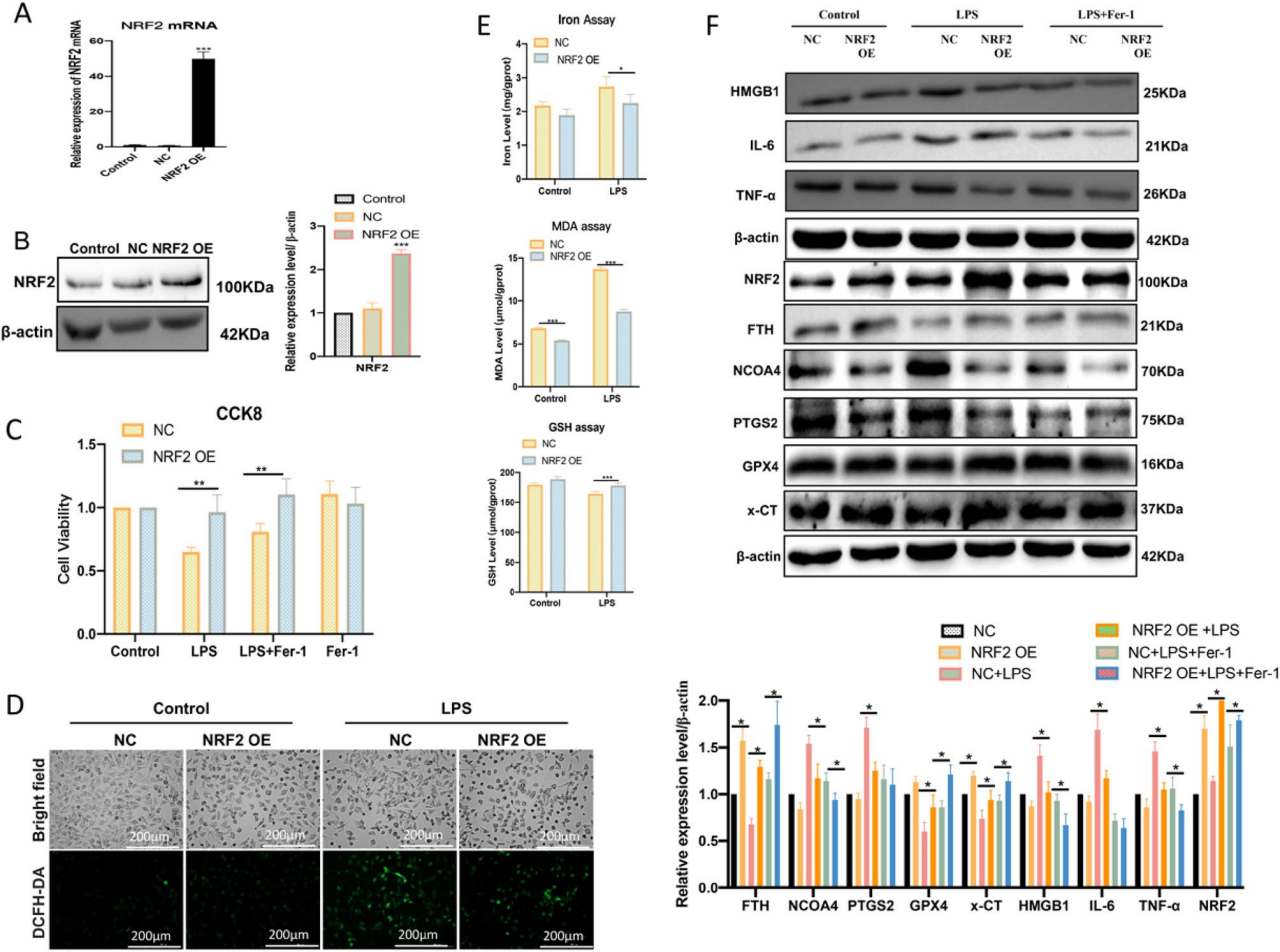
High expression Nrf2 improves LPS-induced HUVECs ferroptosis and inflammation. (**A**,**B**) RT-qPCR and western blot were used to verify the high expression of Nrf2 in endothelial cells by transfecting Nrf2 overexpression plasmid; ***P < 0.001 versus NC or Control group. (**C**) The cell viability 24 h after LPS treatment was assayed by CCK-8; **p < 0.01. (**D**) The DCFH-DA probes were performed for LPS (3 h)-induced ROS levels (scale bar, 200 μm). (**E**) The iron ion, GSH, and MDA levels were measured after LPS treatment for 6 h; *P < 0.05, ***p < 0.001. (**F**) The ferroptosis and inflammation-related protein expression levels were measured by western blot after LPS treatment for 6 h; *P < 0.05.

Recently, several findings have indicated that Nrf2 plays an important protective effect against ferroptosis^20–22^. Based on these the endothelial cell high-expression model of Nrf2 was established by the Nrf2 overexpression plasmid (Fig. 4A,B). Results showed that overexpression of Nrf2 significantly inhibited LPS-induced death of HUVECs (Fig. 4C). The fluorescent images showed that over-expression of Nrf2 significantly decreased LPS-induced ROS levels (Fig. 4D). It also found the contents of intracellular iron ions, GSH depletion, and MDA levels were decreased after overexpression of Nrf2 in LPS-induced HUVECs (Fig. 4E). Furthermore, Nrf2 high expression could increase the protein expression levels of GPX4, x-CT, and FTH, and decrease PTGS2 and NCOA4 levels (Fig. 4F). Detecting inflammation-related proteins also showed that Nrf2 high expression significantly decreased HMGB1, IL-6, and TNF-α expression levels (Fig. 4F).

**Figure 5:**
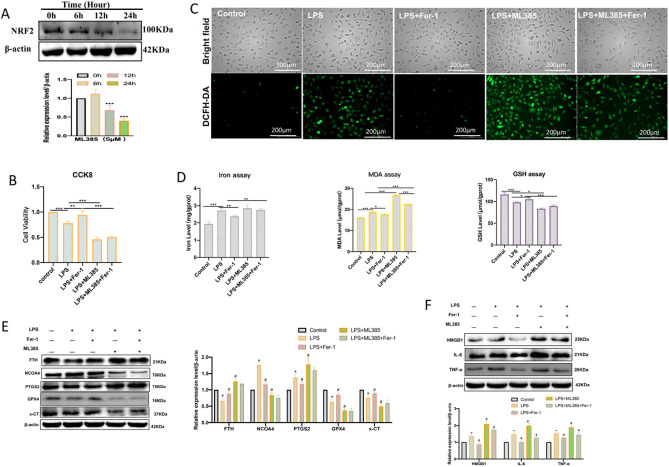
Inhibition of Nrf2 aggravates LPS-induced HUVECs ferroptosis and inflammation. (**A**) Western blot was used to verify the inhibition effect of Nrf2 in endothelial cells by ML385(5 μM) treatment at different times (0, 6, 12, and 24 h); ***P < 0.001 versus 0 h group. (**B**) CCK8 was used to detect cell viability after LPS treatment for 24 h; **P < 0.01, ***P < 0.001. (**C**) The DCFH-DA probes were performed for ROS levels (scale bar, 200 μm) after LPS treatment for 3 h. (**D)** The iron ion, GSH, and MDA levels were measured after LPS treatment for 6 h; *P < 0.05, **P < 0.01, ***P < 0.001. (**E**) The ferroptosis-related protein expression levels were measured by western blot after LPS treatment for 6 h; *P < 0.05 versus Control group, ^#^*P* < 0.05 versus LPS group. (**F**) The LPS (6 h)-induced inflammation-related proteins after ML385(5 μM) treatment were measured by western blot ; *P < 0.05 versus Control group, ^#^*P* < 0.05 versus LPS group, ^¥^*P* < 0.05 versus LPS + ML385 group.

ML385 is an effective inhibitor of Nrf2, and 5 μM can significantly inhibit NRF2 expression activation in HUVECs^23^. We used 5 μM ML385 treatment HUVECs did inhibit the protein expression levels of NRF2 (Fig. 5A). Nrf2 inhibitor ML385 treatment could also significantly aggravate LPS-induced cell death (Fig. 5B), and ROS levels (Fig. 5C). Inhibition of Nrf2 also increased the levels of intracellular iron ions, GSH depletion, and MDA levels (Fig. 5D). Moreover, ML385 treatment could further decrease LPS-induced GPX4, x-CT, and NCOA4 levels, and increase PTGS2 and FTH levels (Fig. 5E). The detection of inflammation-related proteins also showed that ML385 treatment increased LPS-induced protein expression levels of HMGB1, IL-6, and TNF-α (Fig. 5F).

### Nrf2-mediated redox balance plays a protective role in regulating the survival of HUVECs

**Figure 6:**
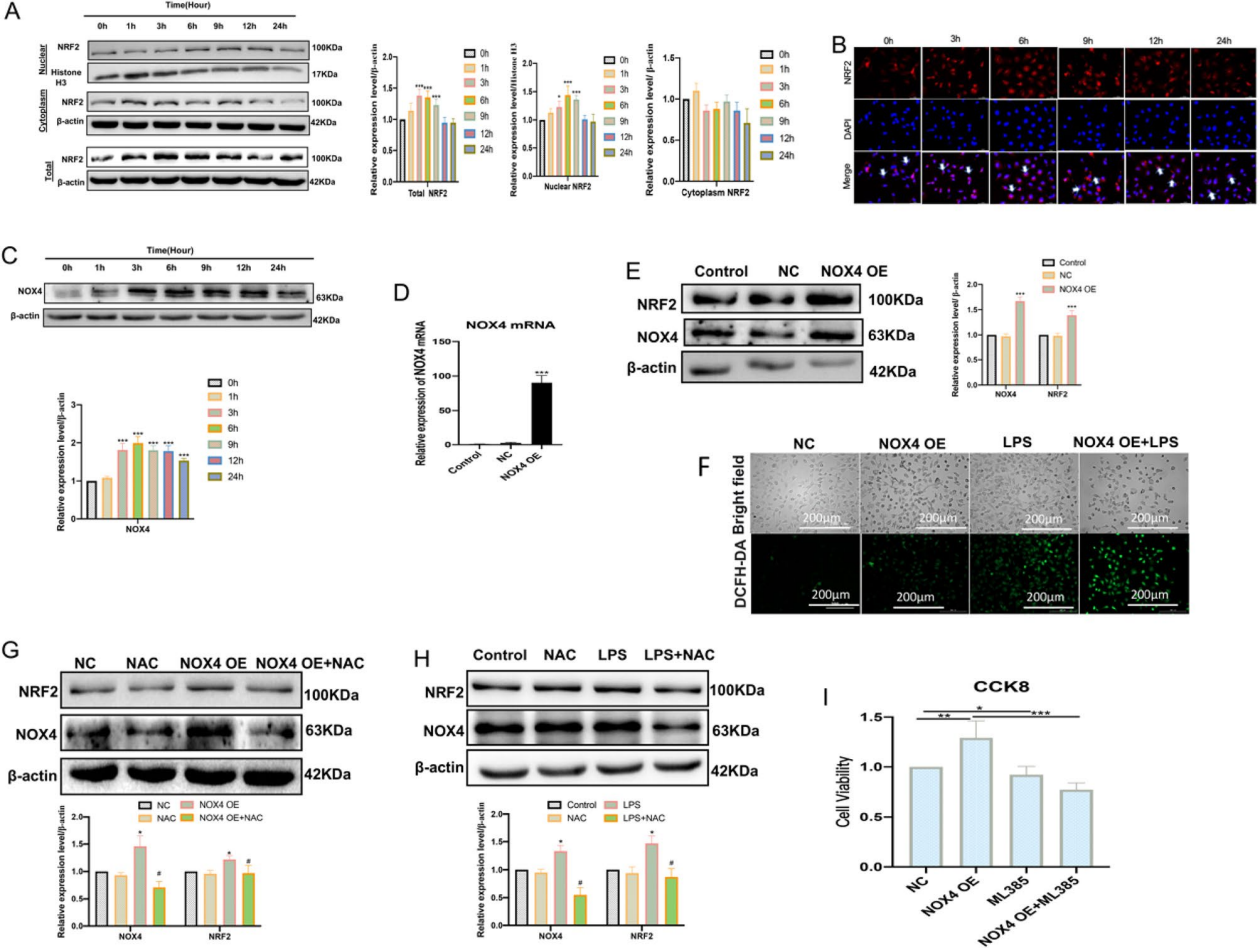
NOX4/Nrf2 redox balance plays a protective role in regulating survival in HUVECs. (**A**) The total, nuclear, and cytosolic NRF2 protein expression levels were measured by western blot after 2 μg/mL LPS treatment (0, 1, 3, 6, 9, 12, and 24 h); *P < 0.05, ***P < 0.001 versus 0 h group. (**B**) Representative immunofluorescence images of NRF2 protein expression in LPS-treated HUVCs (scale bar, 50 μm), Blue: DAPI, Red: NRF2. (**C**) The NOX4 protein expression levels were measured by western blot after 2 μg/mL LPS treatment (0, 1, 3, 6, 9, 12, and 24 h); ***P < 0.001 versus 0 h group. (**D**) RT-qPCR was used to verify the high expression of NOX4 in HUVECs by transfecting NOX4 overexpression plasmid; ***P < 0.001 versus NC or Control group. (**E**) western blot was used to measure the protein expression levels of NOX4 and Nrf2 in HUVECs by transfecting the NOX4 overexpression plasmid; ***P < 0.001 versus NC or Control group. (**F**) DCFH-DA probe was performed for ROS levels (scale bar, 200 μm). (**G**) Western blot was used to assay the expression levels of NRF2 and NOX4 proteins after NAC treatment in HUVECs treated with NOX4 overexpression plasmid; *P < 0.05 versus NC group, ^#^*P* < 0.05 versus NOX4 OE group. (**H**) Western blot was used to assay the expression levels of Nrf2 and NOX4 proteins after NAC treatment in endothelial cells treated with LPS; *P < 0.05 versus Control group, ^#^* P* < 0.05 versus LPS group. (**I**) CCK8 was used to detect cell viability; *P < 0.015, **P < 0.01, ***P < 0.001.

The study also found that LPS could increase the nuclear translocation and nuclear protein expression of Nrf2 in a time-dependent manner, especially in the early stage (after 3–9 h) of LPS treatment (Fig. 6A,B). These indicated that Nrf2 was activated after LPS treatment. Previous research has found that NADPH oxidase-4 (NOX4) is an important modulator of ROS production and redox signaling, which plays an important role in the regulation of Nrf2 activation^24,25^. It found that LPS could increase the expression levels of NOX4 in a time-dependent manner (especially after LPS treatment for 3–12 h) (Fig. 6C). By established HUVECs high-expression model of NOX4 (Fig. 6D,E), the DCFH-DA fluorescent images showed that the over-expression of NOX4 significantly increased LPS-induced ROS levels(Fig. 6F), and over-expression of NOX4 significantly increased the protein expression levels of NRF2 (Fig. 6E). ROS scavenger NAC (5 mM) treatment effectively reduced the protein expression levels of NOX4 and Nrf2 in endothelial cells treated with NOX4 overexpression (Fig. 6G). It also detected that NAC (5 mM) treatment effectively reduced the NOX4 and NRF2 protein expression levels in LPS-stimulated HUVECs (Fig. 6H). Moreover, when HUVECs overexpressing NOX4 were treated with ML385, the increase in cell viability induced by NOX4 overexpression was disrupted. This suggests that Nrf2 plays a protective role in LPS-induced NOX4-mediated survival of HUVECs (Fig. 6I). Together, these suggest that Nrf2-mediated redox balance plays an important role in regulating survival in HUVECs.

## Discussion

Ferroptosis, a new regulatory death mode, is related to the iron-dependent accumulation of lipid peroxidation^7^. Iron overload is related to lipid metabolism disorders, atherosclerotic plaque growth, and instability^19^. Yu et al.^26^ found that ferroptosis was involved in the development of atherosclerotic plaque in a high-level of uric acid-treated ApoE−/− mice. Iron chelation can significantly reduce the aortic inflammatory response and improve endothelial function in mice^27^. Arteriosclerosis is the initial stage of atherosclerosis, and vascular endothelial cell injury is the initial link of arteriosclerosis. To explore the impact of iron ion levels on atherosclerosis, we analyzed the correlation between serum iron ion levels, serum inflammation markers, and blood lipid levels in patients with atherosclerosis. The results indicated that there was a significant positive correlation between serum iron levels and N ratio, N/L, LDL level, and LDL/HDL. This indicates that higher serum iron level is closely related to inflammation and lipid metabolism disorders in arteriosclerosis patients. Ferroptosis may be involved in the pathogenesis of endothelial inflammation injury in arteriosclerosis.

The vascular endothelium is an important regulatory barrier for vascular homeostasis, all kinds of oxidative stress factors can induce endothelial cell dysfunction by increasing reactive oxygen species (ROS) production^28^. In the meantime, increased ROS plays an important role in the pathogenesis of vascular endothelial inflammation^29,30^. Ferroptosis is a new form of non-apoptotic cell death characterized by oxidative toxicity induced by excessive iron ions through Fenton chemical reactions, which is involved in the pathogenesis of many diseases^31^. However, the underlying molecular mechanism of ferroptosis in endothelial inflammation is not fully understood yet. System Xc^−^-glutathione-GPX4 axis inhibition and nuclear receptor coactivator 4 (NCOA4)-mediated ferritinophagy activation are the two important ferroptosis regulator ways^32,33^. In order to verify ferroptosis was involved in the pathological process of vascular endothelial cell inflammation, and to further explore its regulatory mechanism. We used lipopolysaccharide (LPS) to induce inflammation of HUVECs. In our present study, we found significant ferroptosis changes in the LPS-induced HUVECs model, including the increase of oxidative damage markers, total ROS, MDA, and PTGS2, the decrease of antioxidants GSH and GPX4, an increase of iron ion levels and NCOA4, as well as decrease of FTH. However, pretreated with Fer-1, a specific inhibitor of ferroptosis, can partially counteract LPS-induced cell damage and inhibit the changes related to ferroptosis. On these bases, we confirmed that ferroptosis was involved in the pathogenesis of vascular endothelial cell inflammation.

As a key stress-inducible transcription factor, Nrf2 has been reported to play a critical role in modulating ferroptosis by regulating iron/heme homeostasis, glutathione metabolism, oxidative stress, and lipid peroxidation^15^. Overexpression of Nrf2 can protect macrophages against inflammation and ferroptosis^34^. It also detected that inhibition of Nrf2 increased ferroptosis sensitivity in tumor cells and enhanced the antitumor effect of chemotherapy drugs^35^. However, the intrinsic role of Nrf2 on ferroptosis in endothelial injury remains to be investigated. In our present study, we found that overexpression of Nrf2 exerted an anti-ferroptosis effect in LPS-induced HUVECs, such as increased expression levels of GSH, GPX4, x-CT, and FTH, while decreased the expression levels of ROS, MDA, iron ion levels, NCOA4 and PTGS2. Interestingly, inhibition of Nrf2 decreased the expression of NCOA4 and increased the expression of FTH. This may be related to the effect that excess free iron feedback inhibits ferritinophagy to reduce ferroptosis in endothelial cells^36^.

Damage-associated molecular patterns (DAMPs) are endogenous molecules released upon cell death that triggers inflammation and are associated with the pathogenesis of many diseases^37^. High mobility group box 1 (HMGB1) is a DAMP released by ferroptotic cells, which also regulates the production of inflammatory proteins TNF-α and IL-6^38,39^. In our research, LPS induced higher levels of HMGB1, TNF-α, and IL-6, while Fer-1 can partially counteract this damaging effect. Studies have also found that Nrf2 plays a key role in endothelial cell inflammation, which is related to the Nrf2-mediated antioxidant defense mechanism^36^. Our results also found that overexpression of NRF2 could reduce HMGB1, TNF-α, and IL-6 inflammation protein expression levels in LPS-induced HUVECs. Thus, these results suggested that ferroptosis was involved in the inflammation of vascular endothelial cells, and Nrf2 plays an important protective role in ameliorating ferroptosis-mediated inflammation in endothelial cells.

Redox balance is an important mechanism for cells to resist oxidative damage, which can effectively improve the damage of oxidative stressors and restore resistance to chronic diseases^40^. Nrf2 is a key transcription factor that regulates cellular redox status^41^. In our study, we found that NRF2 was activated during the early stages in LPS-treated HUVECs. Studies have indicated that ROS produced by NOX4 is a key mediator in inducing NRF2 activation^42^. In our results, we also found high protein expression levels of NOX4 and NRF2 in the early stage of LPS-stimulated HUVECs. Overexpressed NOX4 significantly increases the ROS and protein expression level of NRF2 in HUVECs. However, these activation effects could be eliminated by the ROS inhibitor NAC. In addition, we also detected that overexpressed NOX4 is beneficial to the survival of HUVECs, and this survival effect depends on the presence of NRF2. These suggested that NOX4/Nrf2 redox balance may play an important role in regulating survival in HUVECs. Therefore, our study suggested that maintaining NOX4/Nrf2 redox balance in endothelial cells would be a promising therapeutic strategy to improve endothelial function.

**Figure 7:**
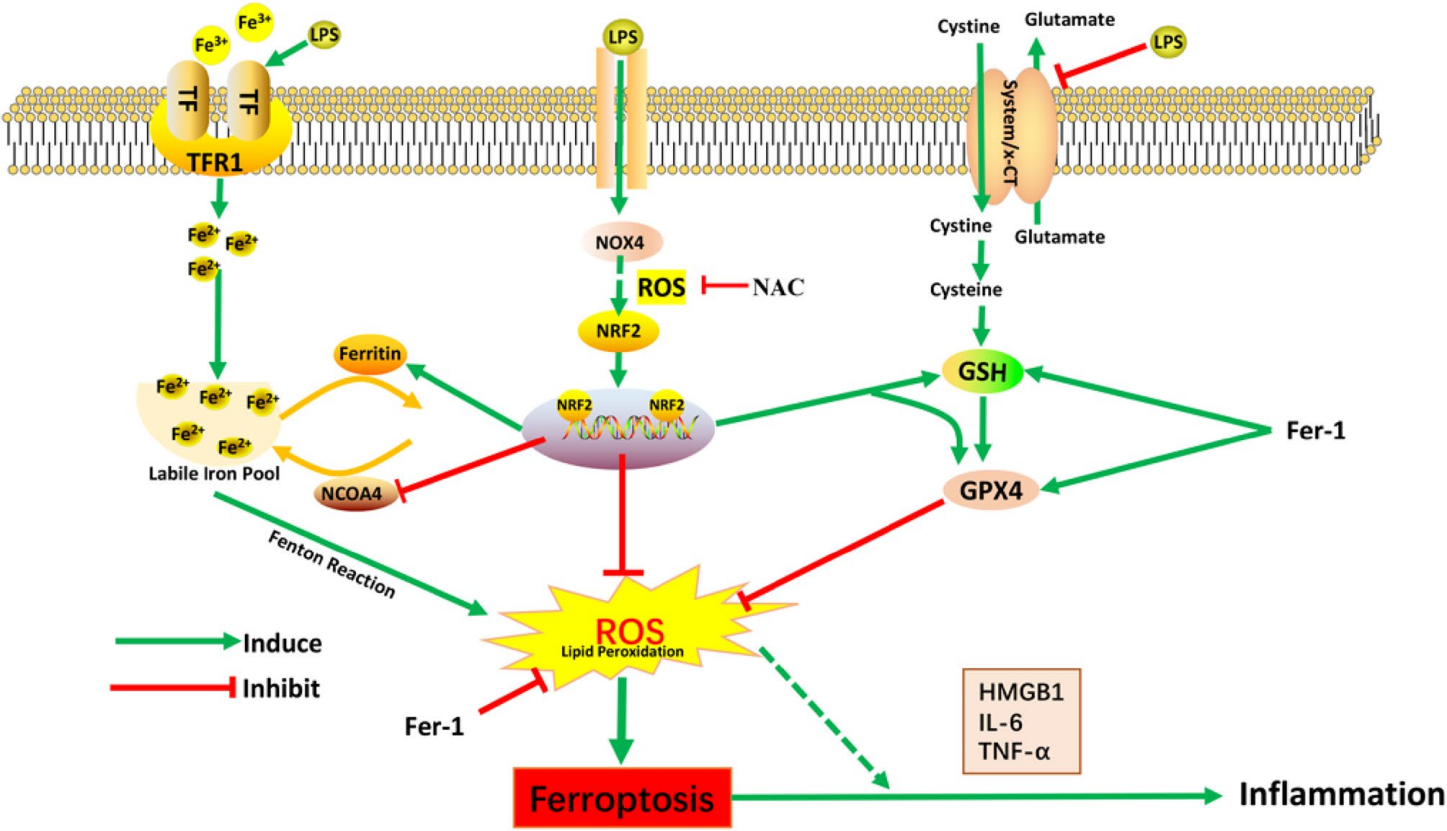
The mechanisms of the Nrf2-mediated redox balance regulate HUVECs ferroptosis and inflammation. Under the condition of lipopolysaccharide (LPS) treatment, the expression of GPX4 and x-CT decreased, and ferritinophagy increased. Ferroptosis inhibitor Fer-1 improved these damaging effects and ameliorated inflammation of HUVECs. Antioxidant factor Nrf2 negatively regulated ferroptosis in HUVECs. The NOX4/Nrf2 redox balance plays an important role in regulating endothelial cell redox homeostasis and cell survival.

In conclusion, our present study confirmed that ferroptosis contributed to the pathogenesis of vascular endothelial cell damage by mediating endothelial cell inflammation. Ferroptosis inhibitor Fer-1 can effectively alleviate LPS-induced vascular damage. In this process, overexpression of Nrf2 can improve LPS-induced vascular endothelial cell inflammation by inhibiting ferroptosis. It was also revealed that maintaining NOX4/NRF2 redox balance plays an important protective role in regulating endothelial cell redox homeostasis and cell survival (Fig. 7). Therefore, this study demonstrates that the treatment targeting ferroptosis and NOX4/NRF2 redox balance will provide a new basis for improving the prognosis of vascular injury-related diseases.

### Supplementary Information

Supplementary Information.

## Data Availability

The data set used and/or analyzed in the current research can be obtained from the corresponding author upon reasonable request.
