# Supplementary material for: Nrf2-mediated redox balance alleviates LPS-induced vascular endothelial cell inflammation by inhibiting endothelial cell ferroptosis
Source: Sci Rep. 2024 Feb 9;14:3335. doi: 10.1038/s41598-024-53976-3 (PMC10858270; doi:10.1038/s41598-024-53976-3)
Supplement: Supplementary file 1 — Supplementary Information. [file 41598_2024_53976_MOESM1_ESM.pdf]

Dear editor

Thank you very much for your interest in our research work. Regarding our original blots, we have rechecked the data and displayed the results of multiple exposure images. We have also outlined the membrane edges with black box lines. Although we made efforts to supplement the imprinting results, there are still some imprints where the marker is not clearly visible due to the exposure process, despite not using a multiple exposure protocol.. However, we want to emphasize that we have not edited or spliced any protein images, and all the images are authentic.

To eliminate any ambiguity, we have taken measures to better present our protein data. We have provided detailed experimental methods and procedures, explaining the antibody incubation conditions and protein staining process (in the experimental methods section of the manuscript).

Furthermore, the molecular weight of the antibodies described in the manual differs from the actual molecular weight of the markers corresponding to our experimental results. This discrepancy may be related to cell type or measurement errors of the markers.

We understand the rigor and integrity principles of scientific research, and in our study, we have always adhered to the principles of honesty, transparency, and reproducibility.

Thank you again for your interest and support in our research work. We look forward to sharing the results of this important study with you.

Sincerely,
Huimin Hou

Fig 2B

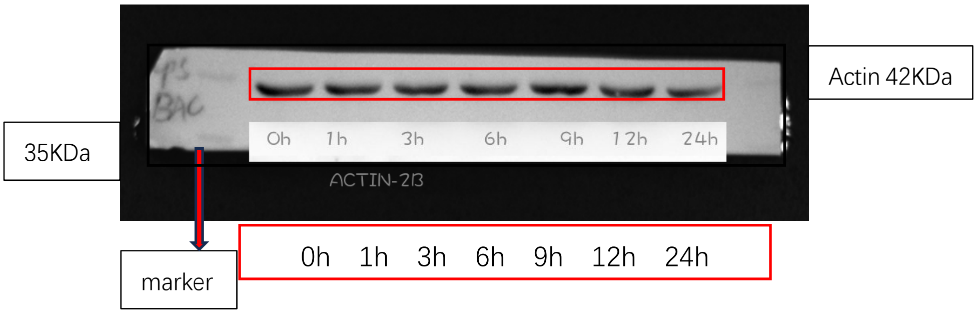

multiple exposure images of IL–6

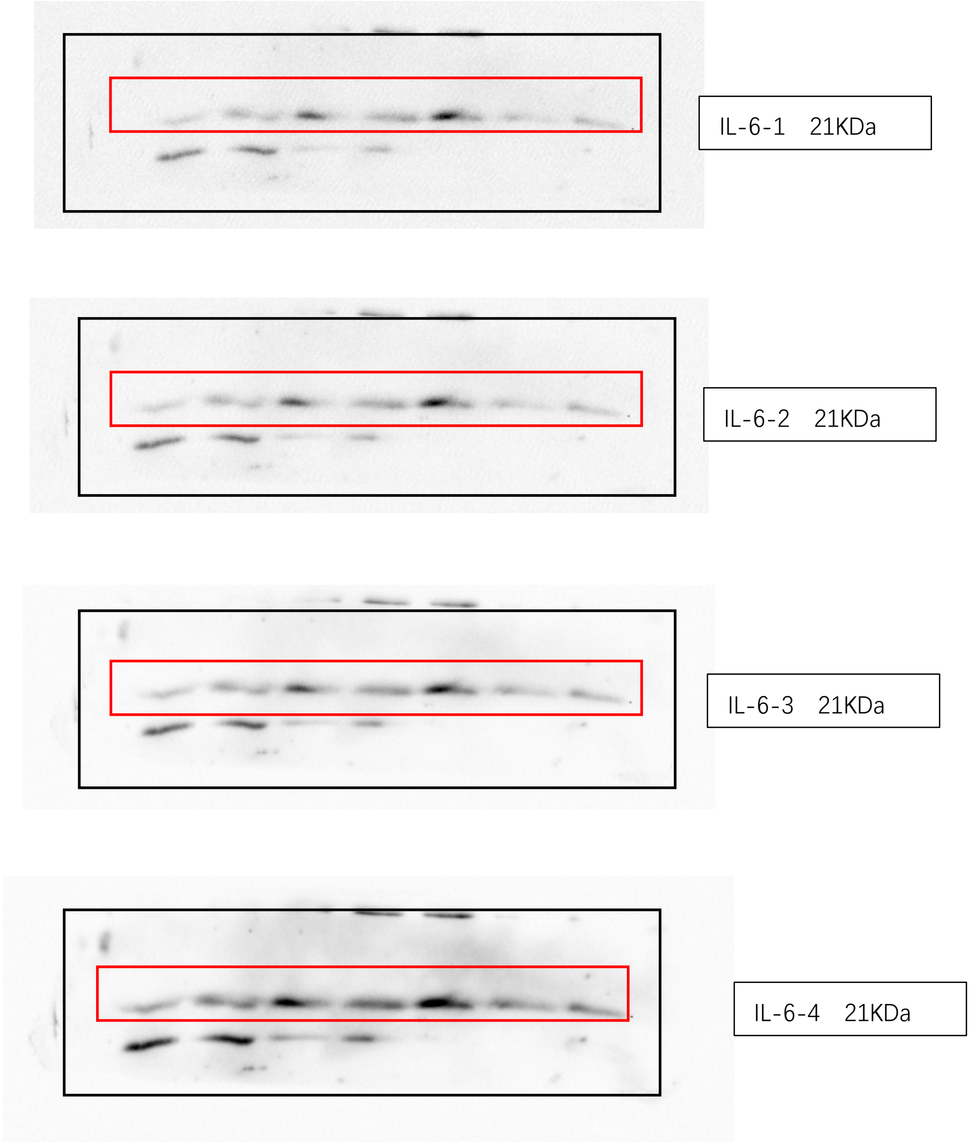

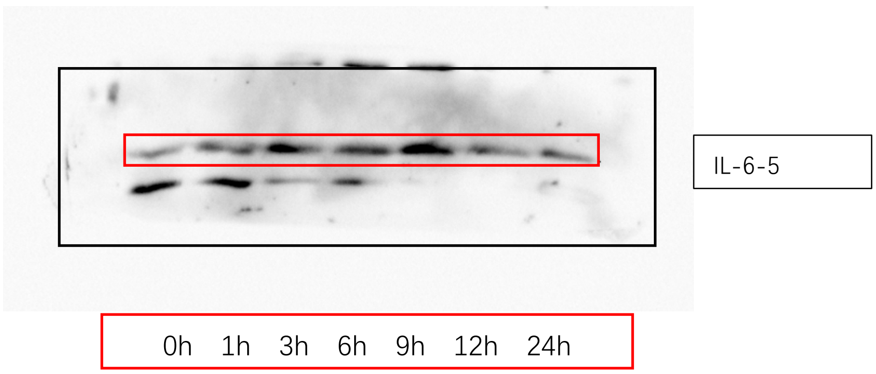

multiple exposure images of TNF-a

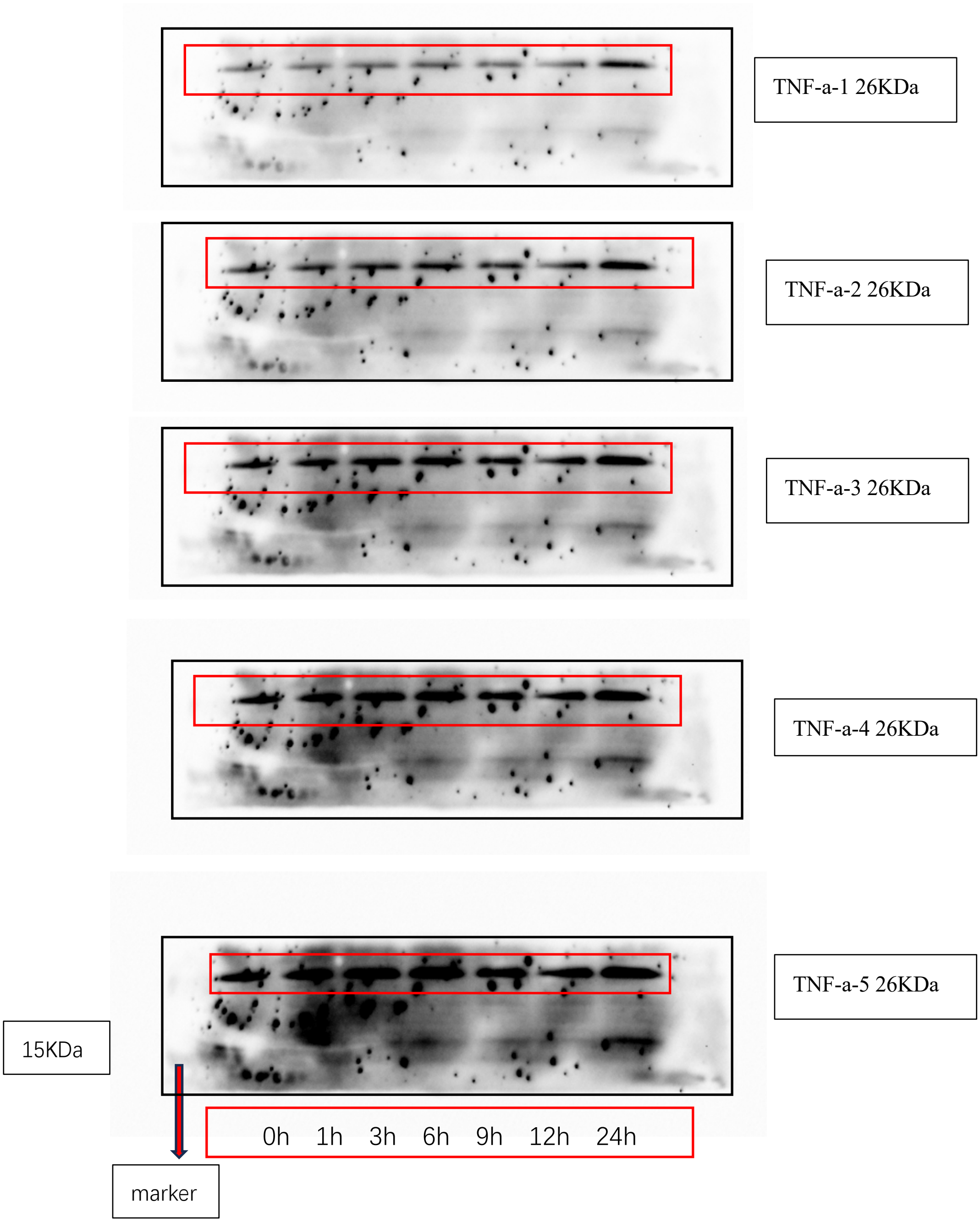

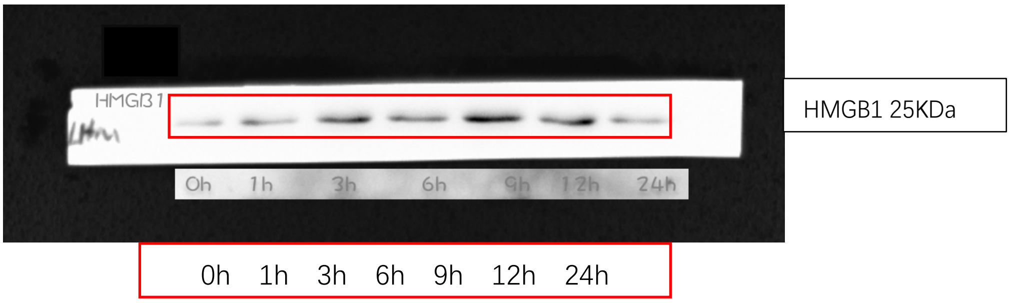
HMGB1
0h 1h 3h 6h 9h 12h 24h
HMGB1 25KDa
0h 1h 3h 6h 9h 12h 24h

Fig 2E

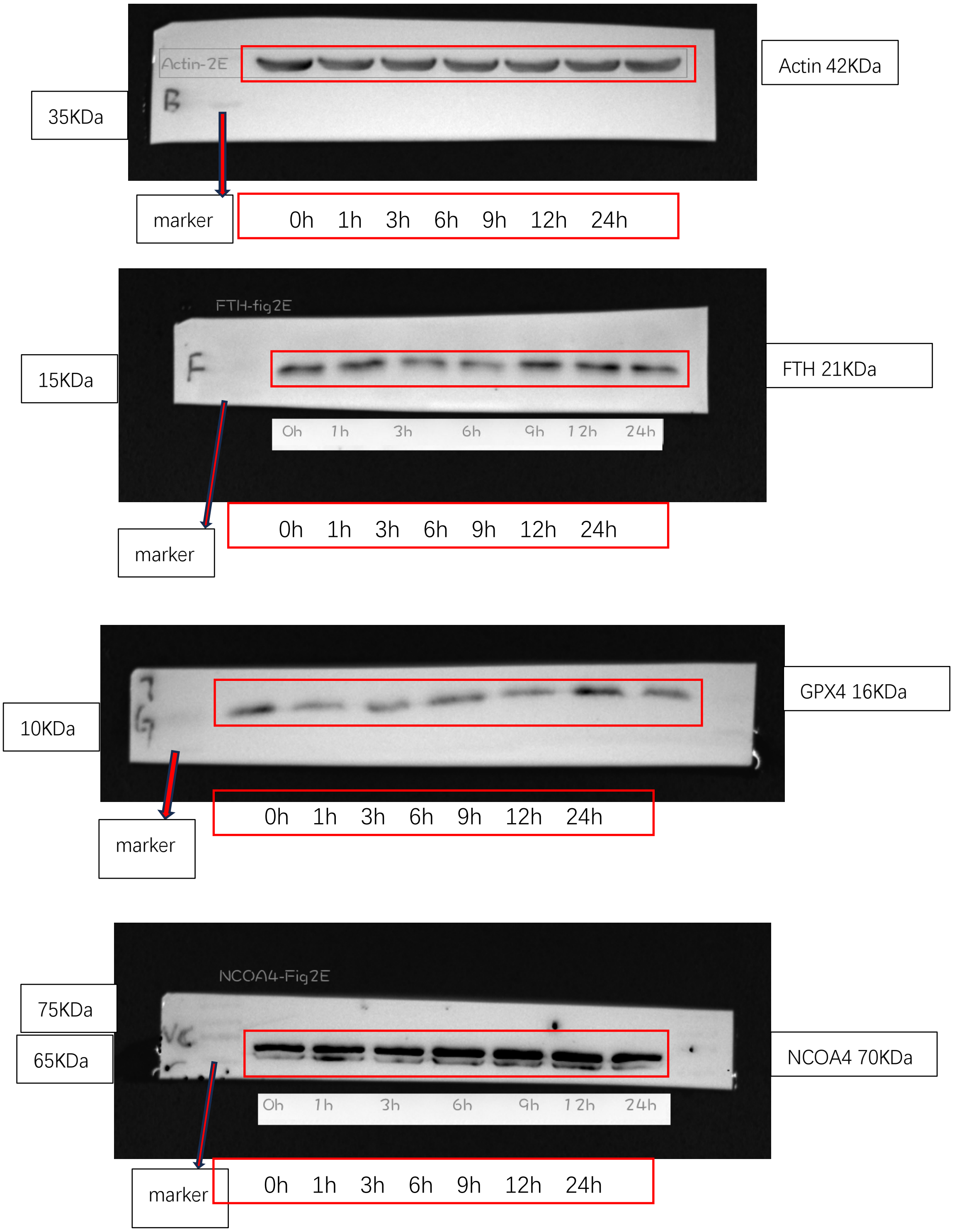
Actin-2E
Actin 42KDa
35KDa
marker
0h 1h 3h 6h 9h 12h 24h
FTH-fig2E
FTH 21KDa
15KDa
0h 1h 3h 6h 9h 12h 24h
marker
0h 1h 3h 6h 9h 12h 24h
GPX4 16KDa
10KDa
marker
0h 1h 3h 6h 9h 12h 24h
NCOA4-Fig2E
75KDa
65KDa
NCOA4 70KDa
0h 1h 3h 6h 9h 12h 24h
marker
0h 1h 3h 6h 9h 12h 24h

multiple exposure images of PTGS2

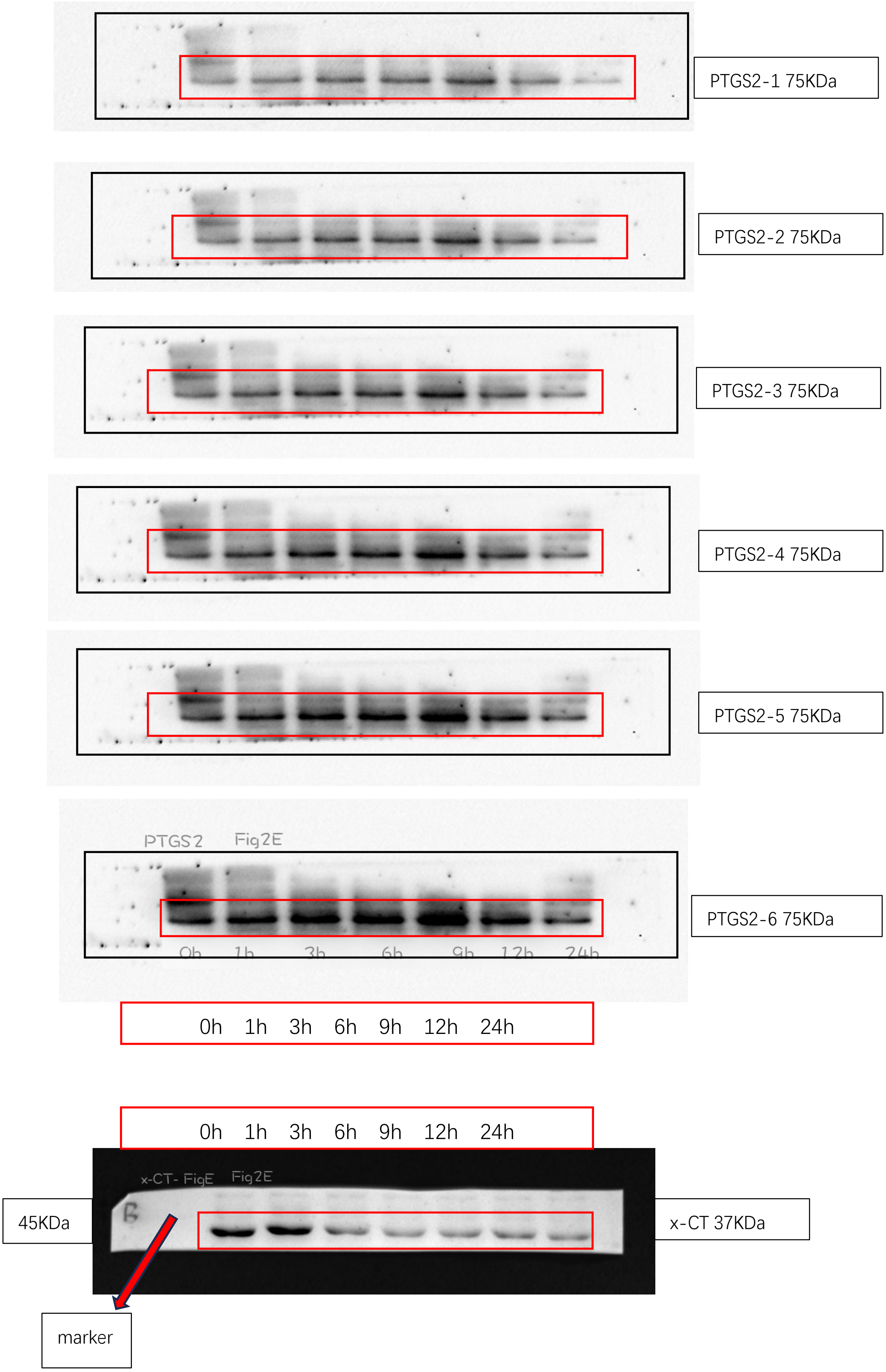

Fig 3E

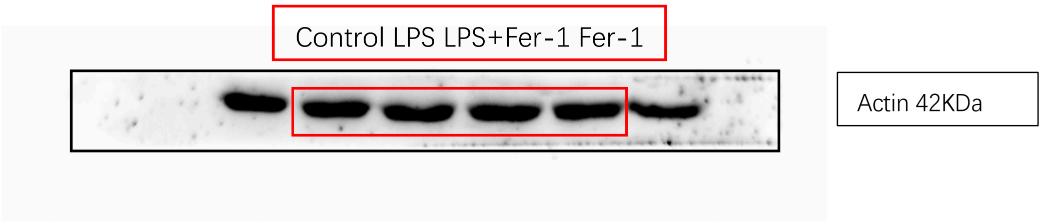

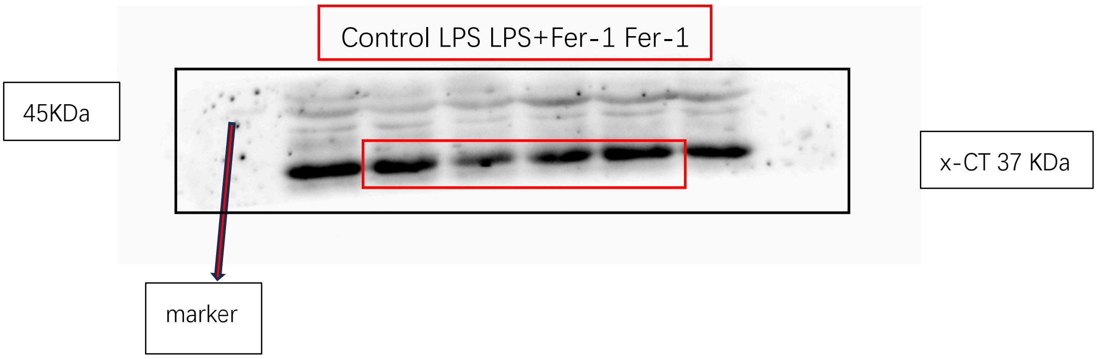

multiple exposure images of Actin

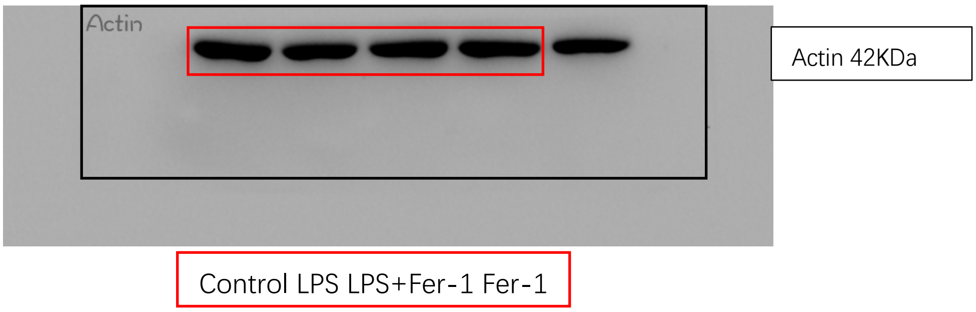

multiple exposure images of IL-6

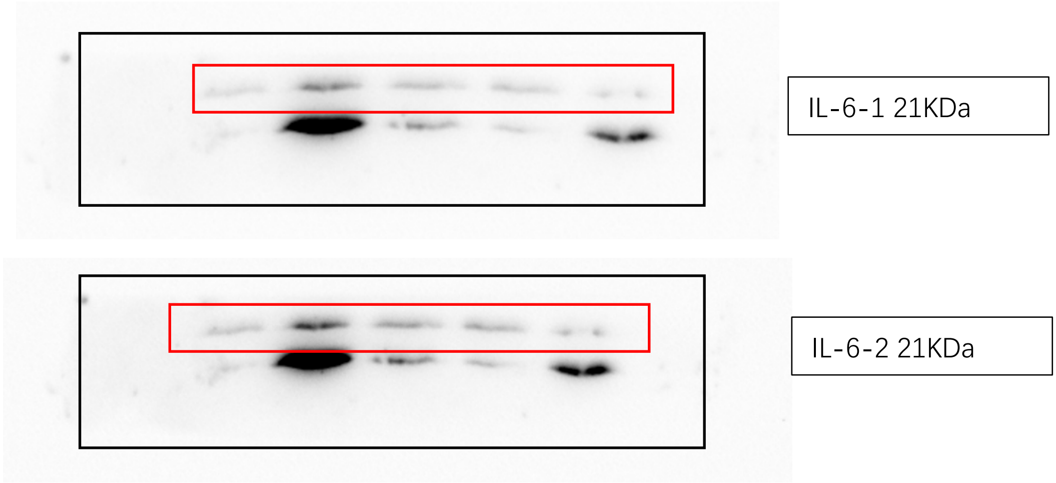

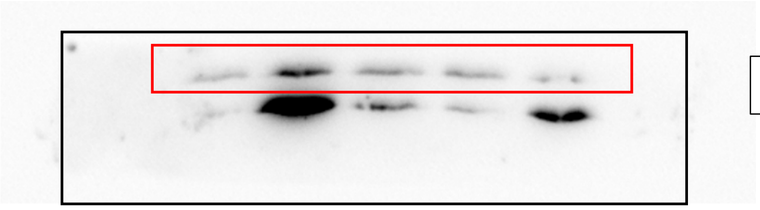

IL-6-3 21KDa

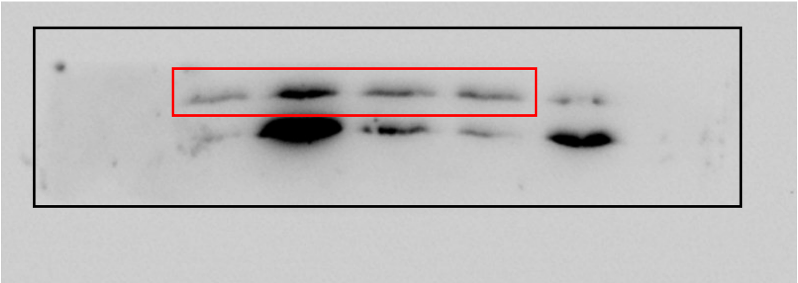

IL-6-4 21KDa

multiple exposure images of TNF-a

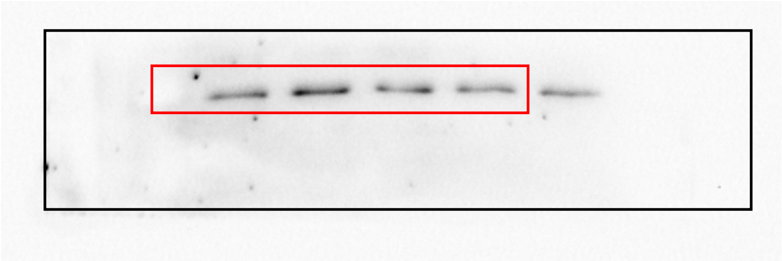

TNF-a-1 26KDa

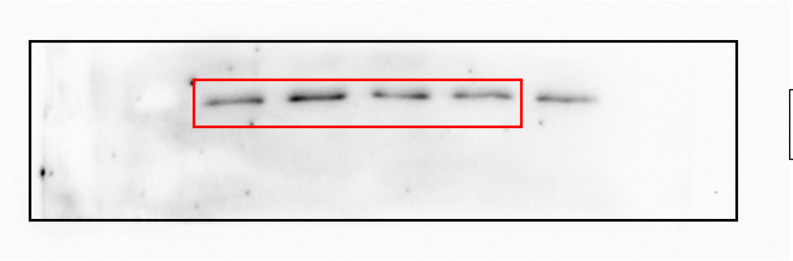

TNF-a-2 26KDa

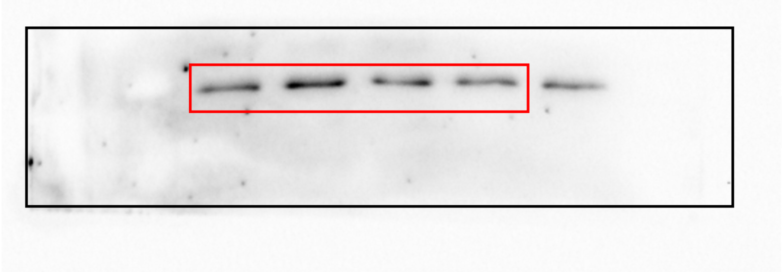

TNF-a-3 26KDa

Control LPS LPS+Fer-1 Fer-1

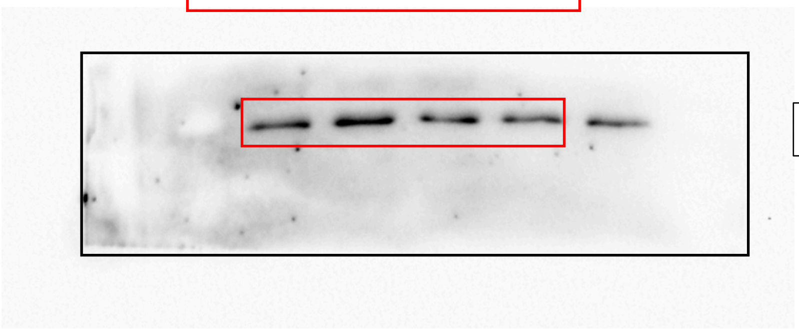

TNF-a-4 26KDa

Control LPS LPS+Fer-1 Fer-1

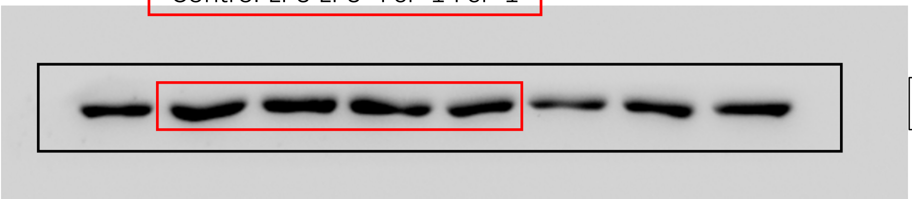

Actin 42KDa

Control LPS LPS+Fer-1 Fer-1

multiple exposure images of HMGB1

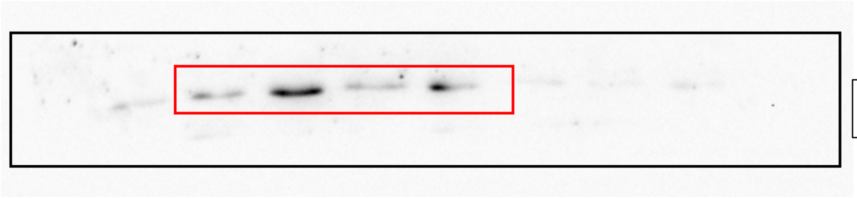

HMGB1-1 25KDa

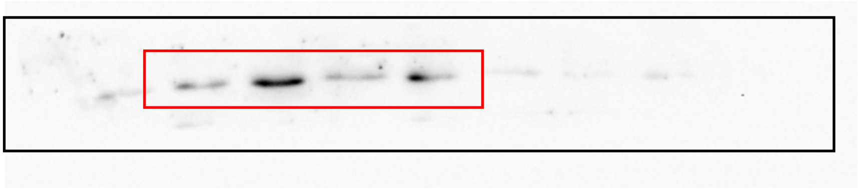

HMGB1-2 25KDa

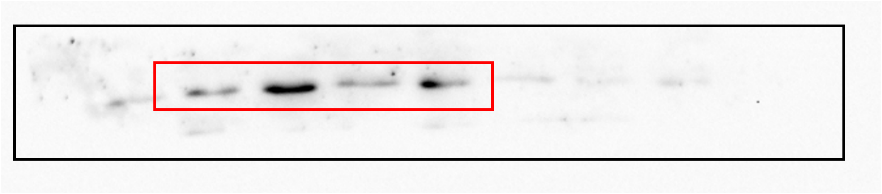

HMGB1-3 25KDa

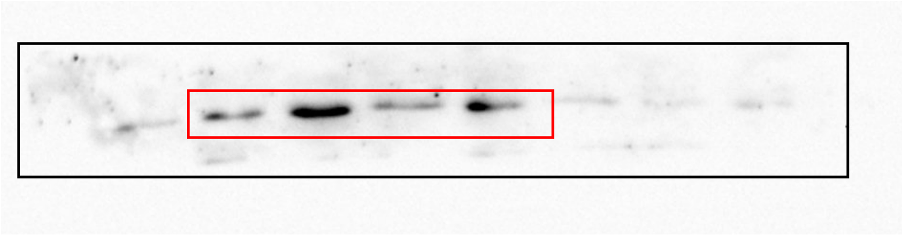

HMGB1-4 25KDa

Control LPS LPS+Fer-1 Fer-1

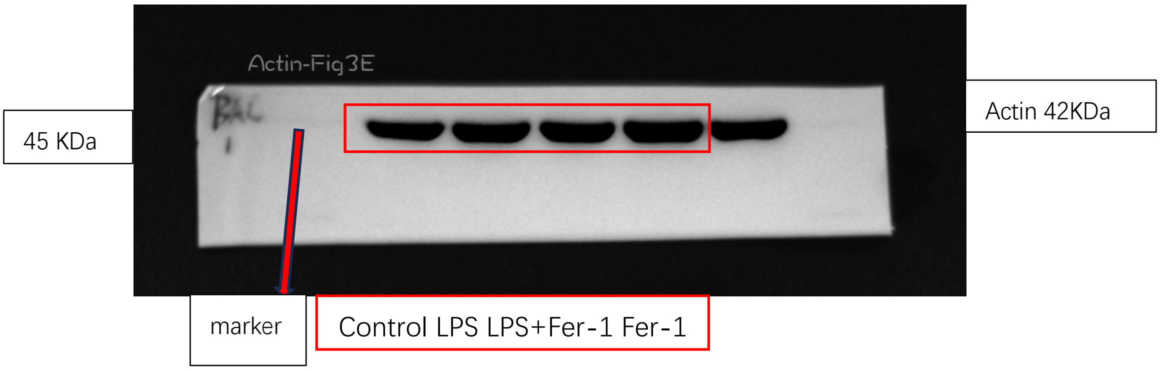

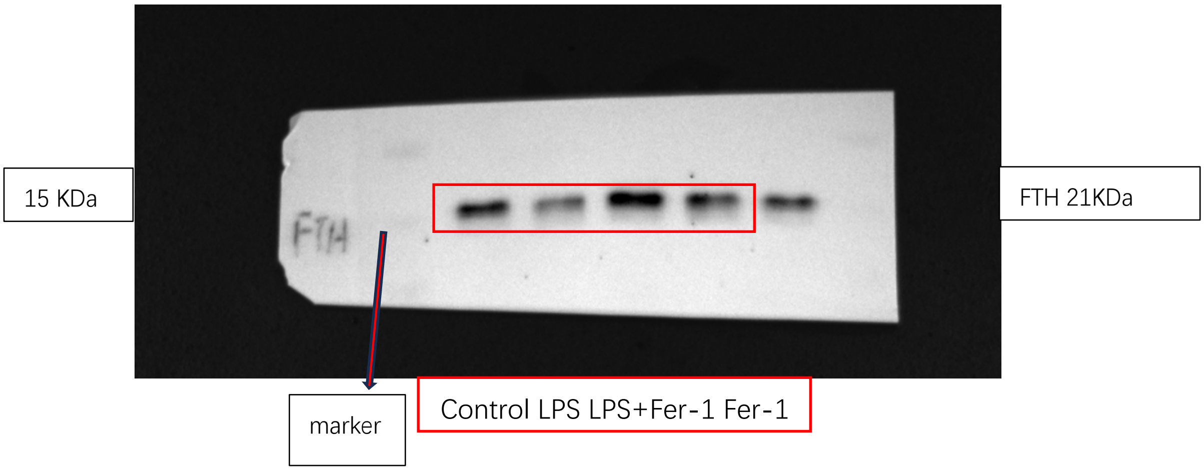

multiple exposure images of GPX4

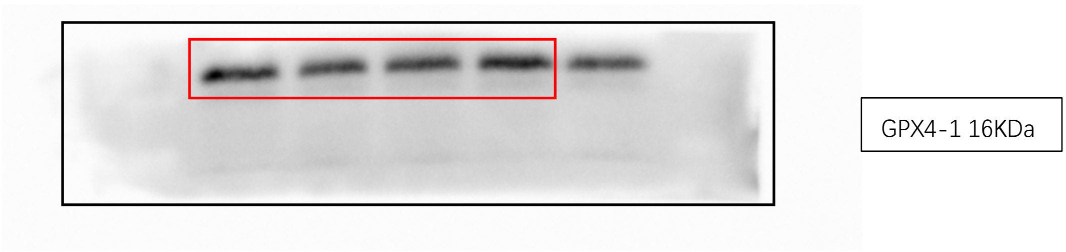

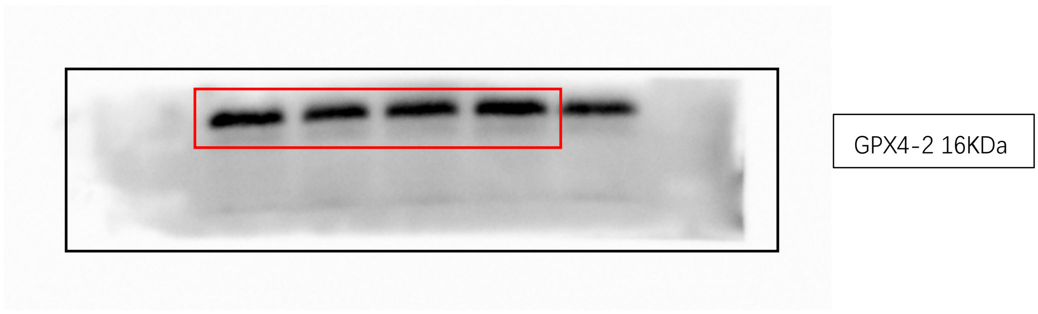

10 KDa

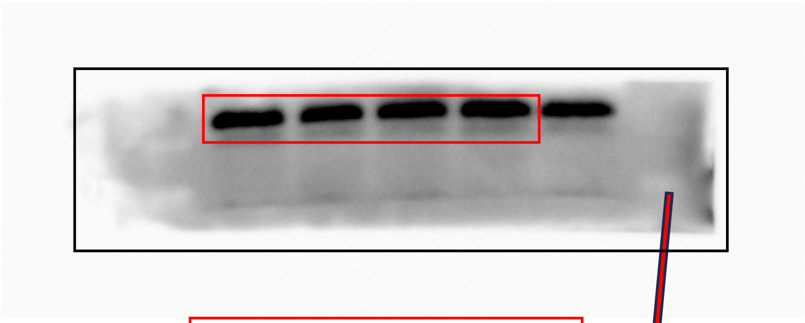

GPX4-3 16KDa

Control LPS LPS+Fer-1 Fer-1

marker

marker

Fig4 B

45 KDa

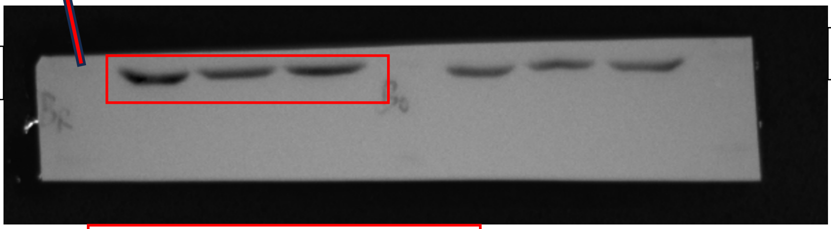

Actin 42KDa

Control NC NRF2 OE

multiple exposure images of NRF2

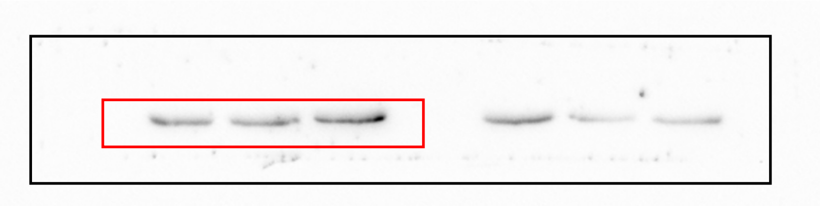

NRF2-1 100KDa

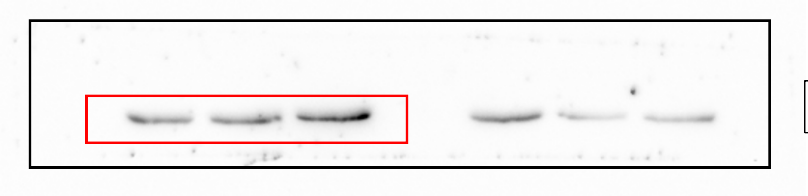

NRF2-2 100KDa

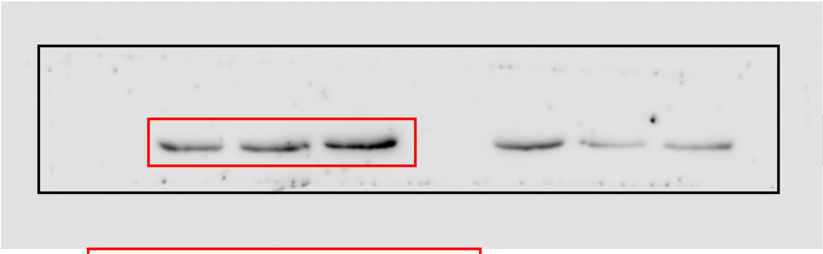

NRF2-3 100KDa

Control NC NRF2 OE

Fig 4F

multiple exposure images of Actin

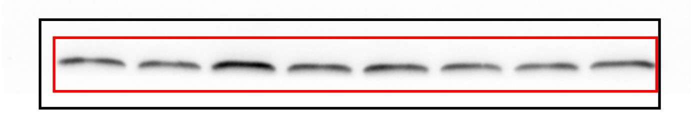

Actin-1 42KDa

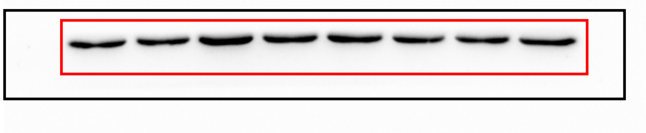

Actin-2 42KDa

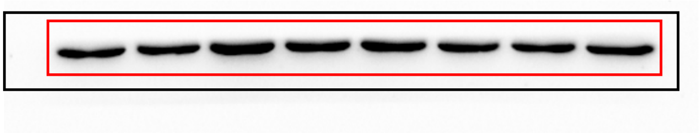

Actin-3 42KDa

Control		LPS		LPS+Fer-1	
NC	NRF2 OE	NC	NRF2 OE	NC	NRF2 OE

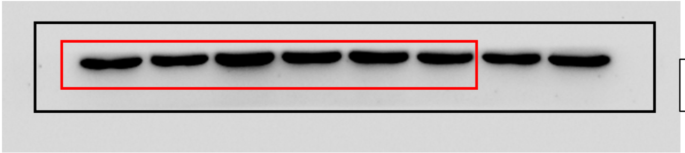

Actin 42KDa

multiple exposure images of x–CT

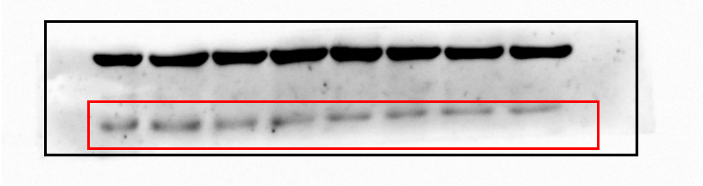

x-CT-1 37KDa

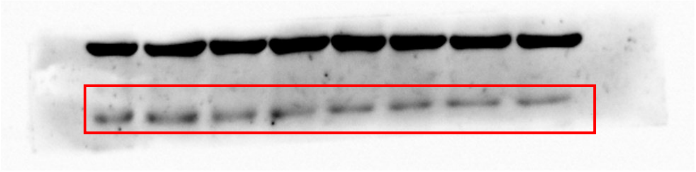

x-CT-2 37KDa

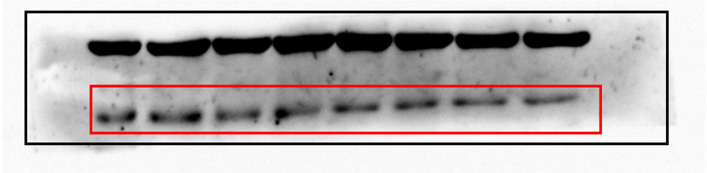

x-CT-3 37KDa

Control		LPS		LPS+Fer-1	
NC	NRF2 OE	NC	NRF2 OE	NC	NRF2 OE

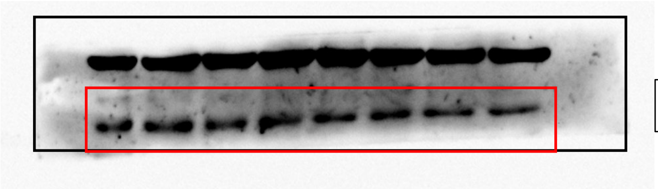

x-CT-4 37KDa

multiple exposure images of GPX4

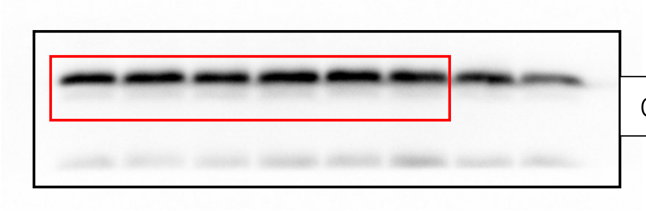

GPX4-1 16KDa

Control: NC, NRF2 OE | LPS: NC, NRF2 OE | LPS+Fer-1: NC, NRF2 OE

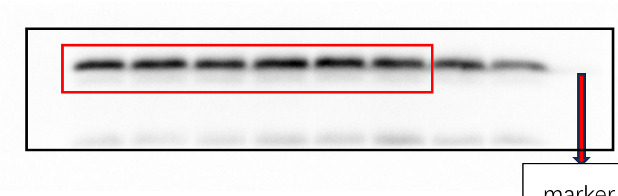

15 KDa

GPX4-2 16KDa

marker

Control: NC, NRF2 OE | LPS: NC, NRF2 OE | LPS+Fer-1: NC, NRF2 OE

25 KDa

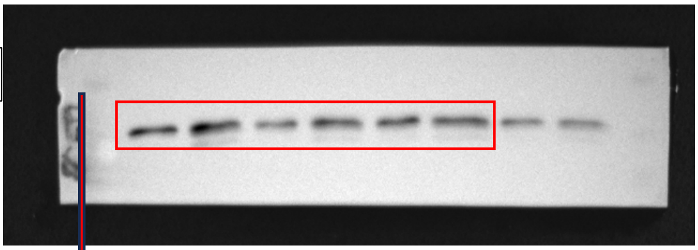

FTH 21KDa

marker

multiple exposure images of NCOA4

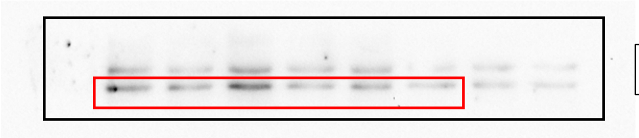

NCOA4-1 70KDa

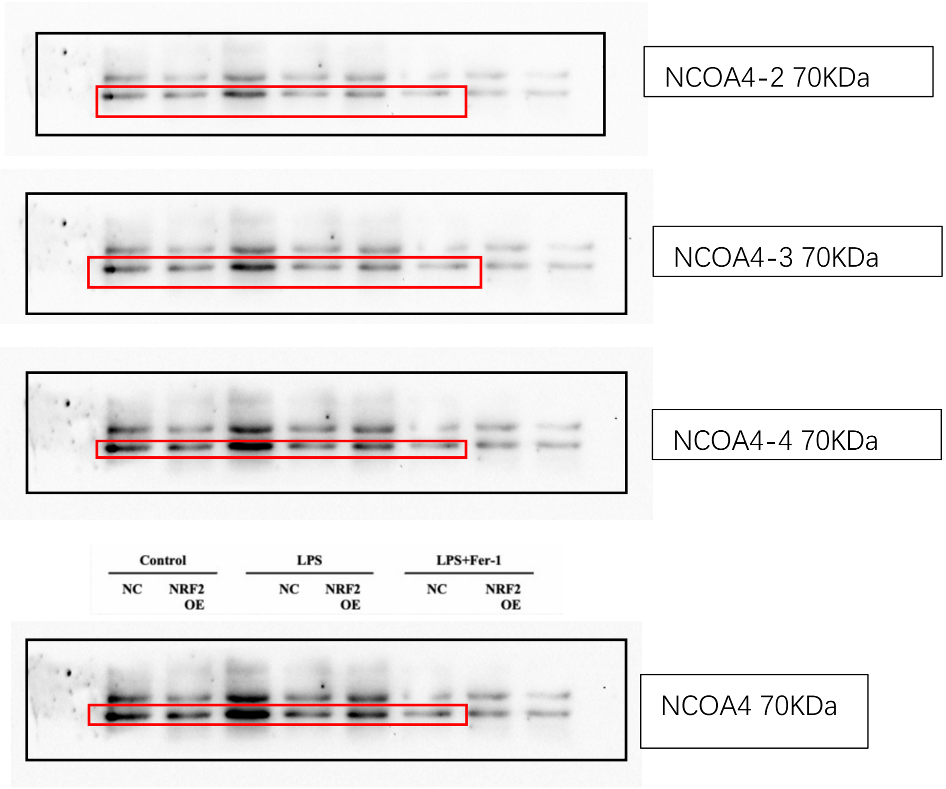
NCOA4-2 70KDa
NCOA4-3 70KDa
NCOA4-4 70KDa
Control
LPS
LPS+Fer-1
NC
NRF2 OE
NC
NRF2 OE
NC
NRF2 OE
NCOA4 70KDa

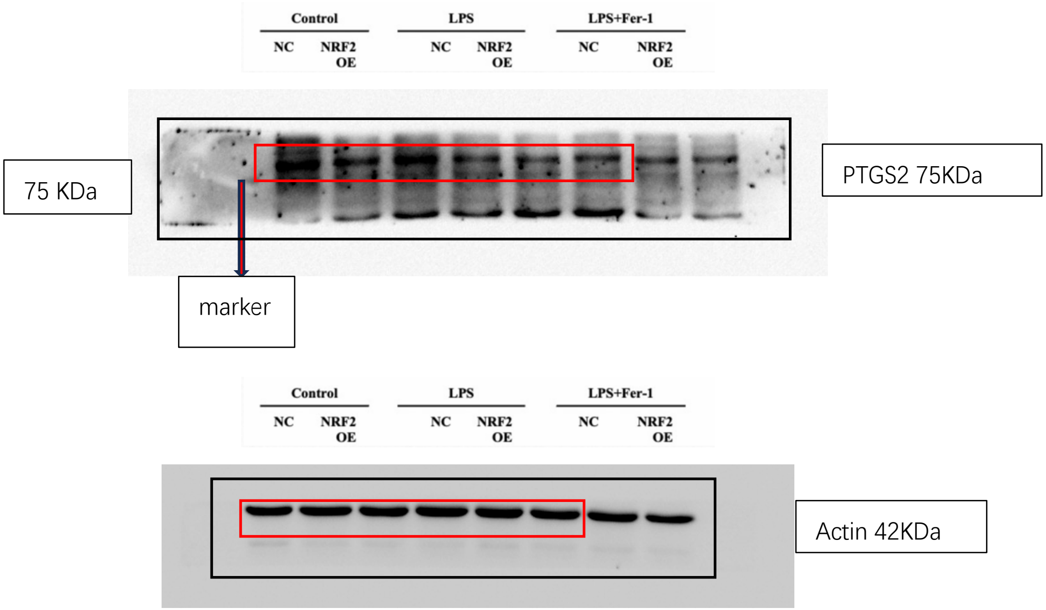
Control
LPS
LPS+Fer-1
NC
NRF2 OE
NC
NRF2 OE
NC
NRF2 OE
75 KDa
PTGS2 75KDa
marker
Control
LPS
LPS+Fer-1
NC
NRF2 OE
NC
NRF2 OE
NC
NRF2 OE
Actin 42KDa

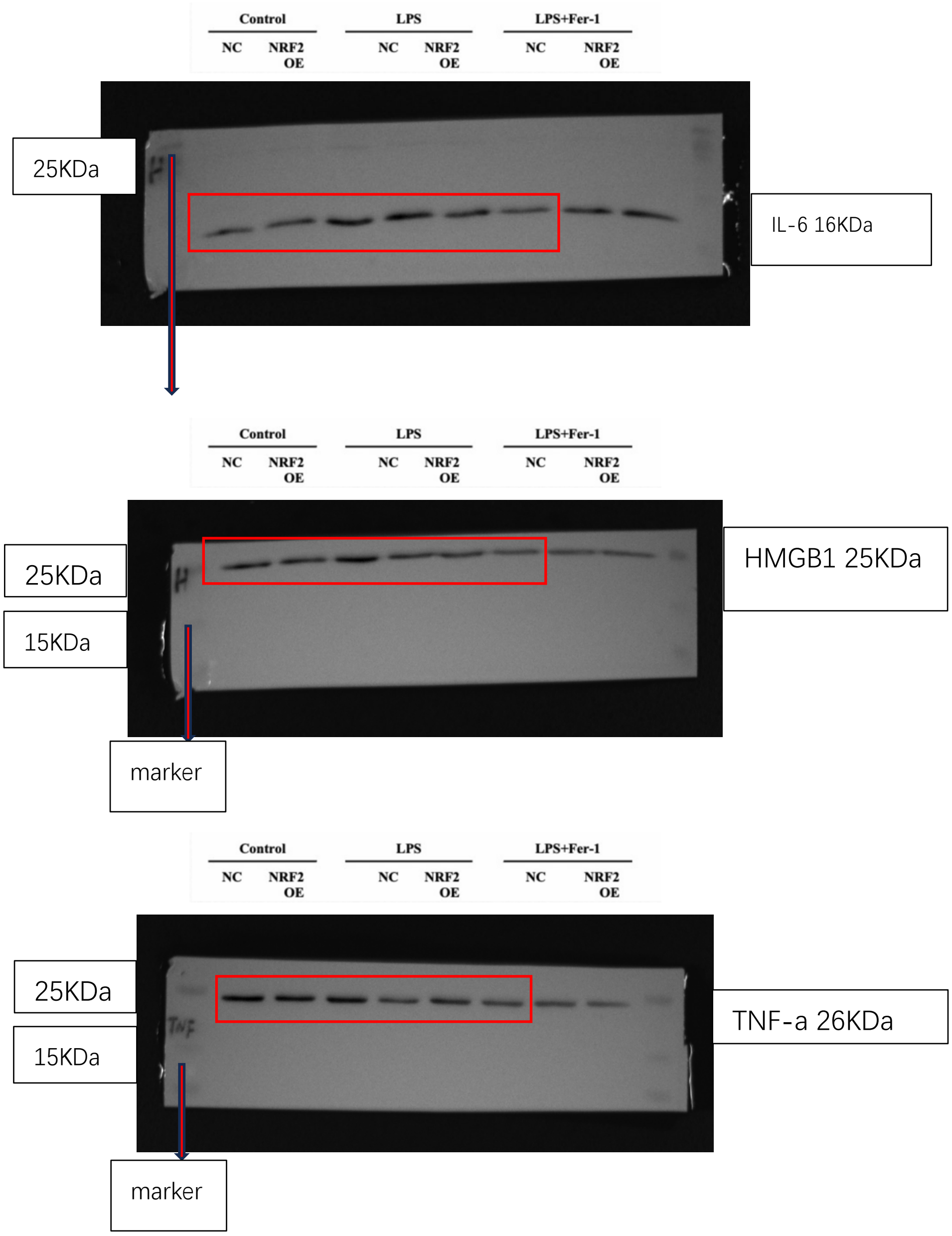
Control
LPS
LPS+Fer-1
NC
NRF2 OE
NC
NRF2 OE
NC
NRF2 OE
25KDa
IL-6 16KDa
Control
LPS
LPS+Fer-1
NC
NRF2 OE
NC
NRF2 OE
NC
NRF2 OE
25KDa
15KDa
HMGB1 25KDa
marker
Control
LPS
LPS+Fer-1
NC
NRF2 OE
NC
NRF2 OE
NC
NRF2 OE
25KDa
15KDa
TNF-a 26KDa
marker

Fig 5A

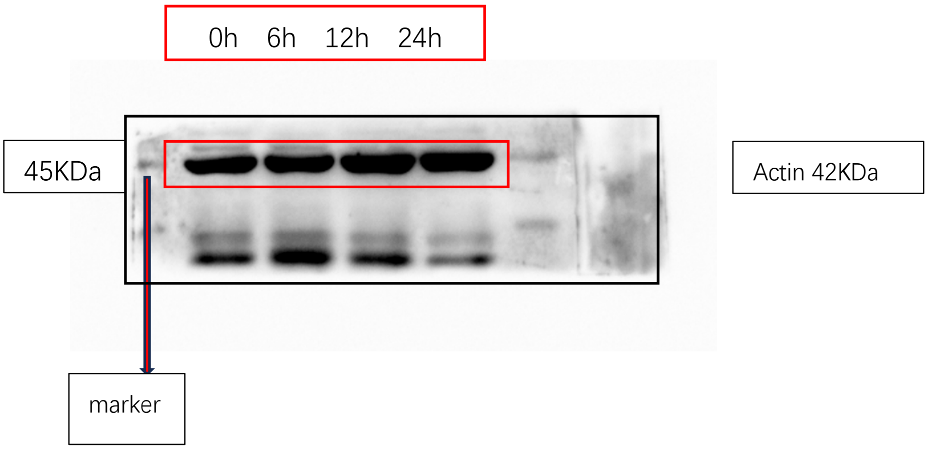
0h 6h 12h 24h
45KDa
Actin 42KDa
marker

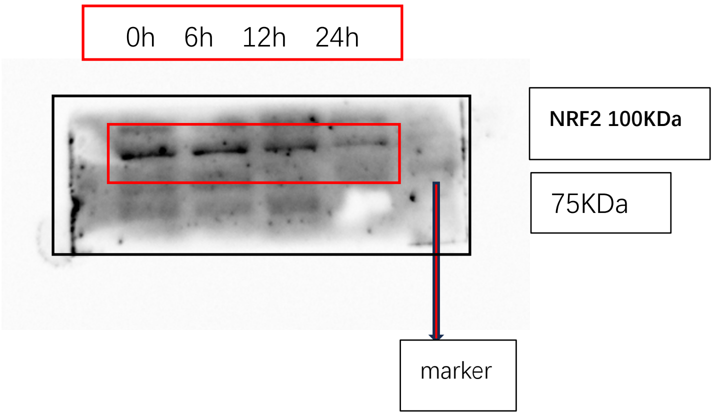

Fig 5E

multiple exposure images of Actin

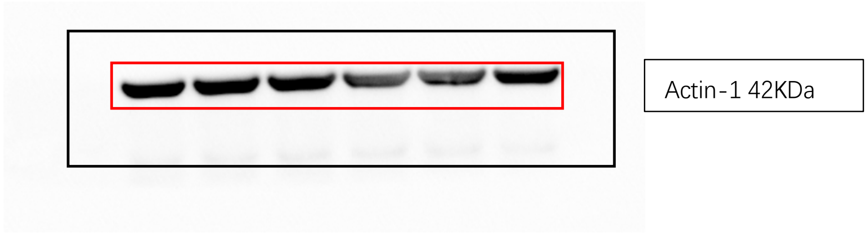

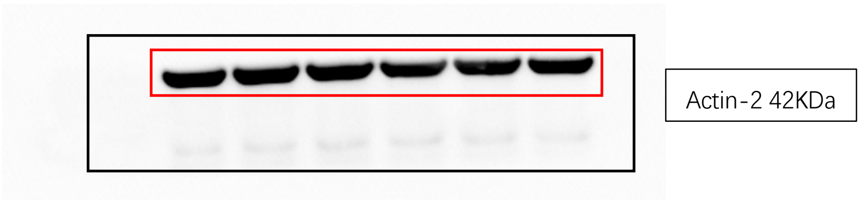

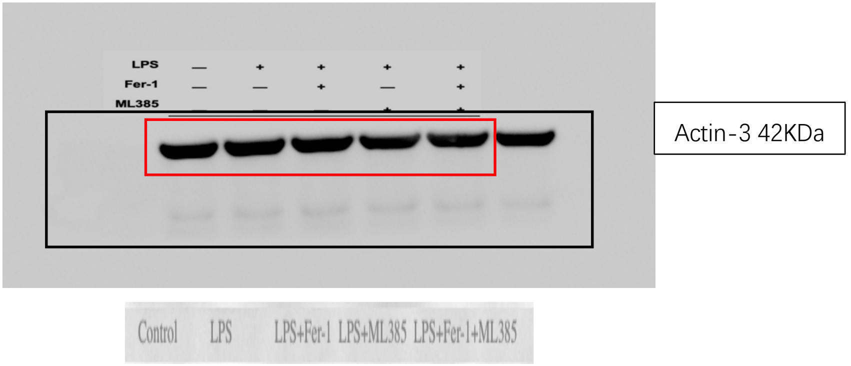

multiple exposure images of FTH

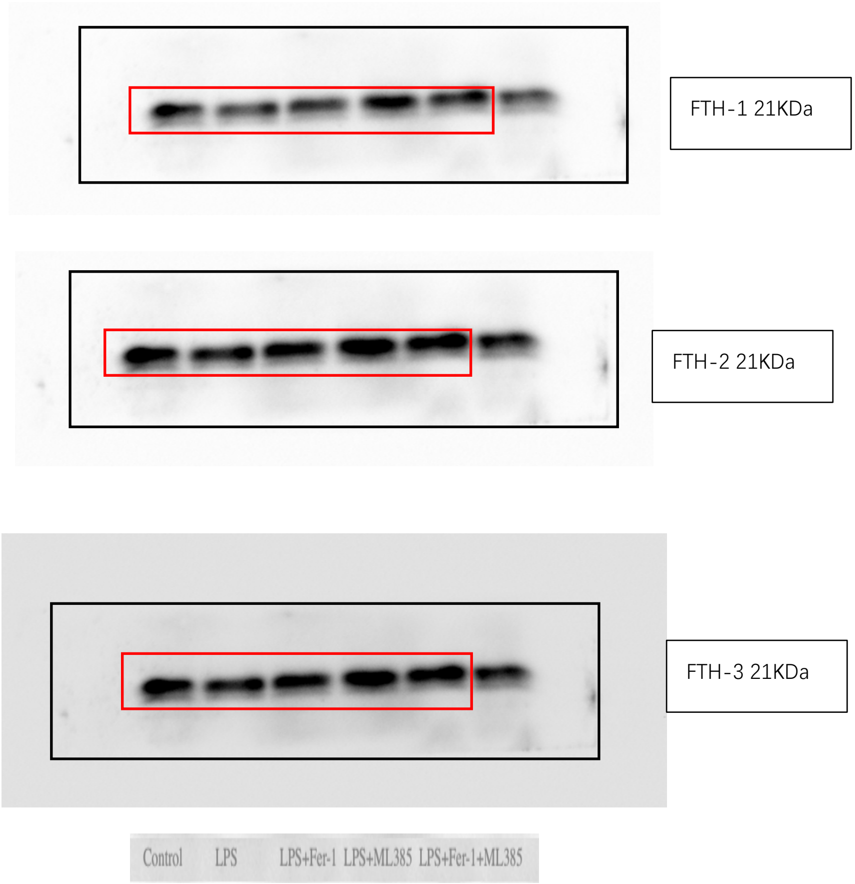

multiple exposure images of NCOA4

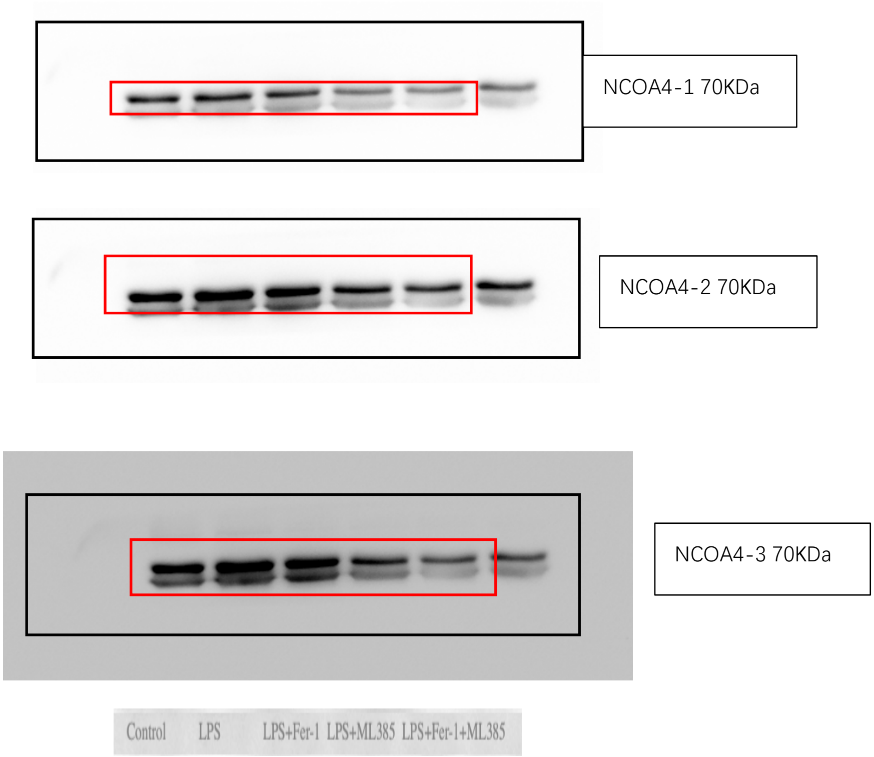

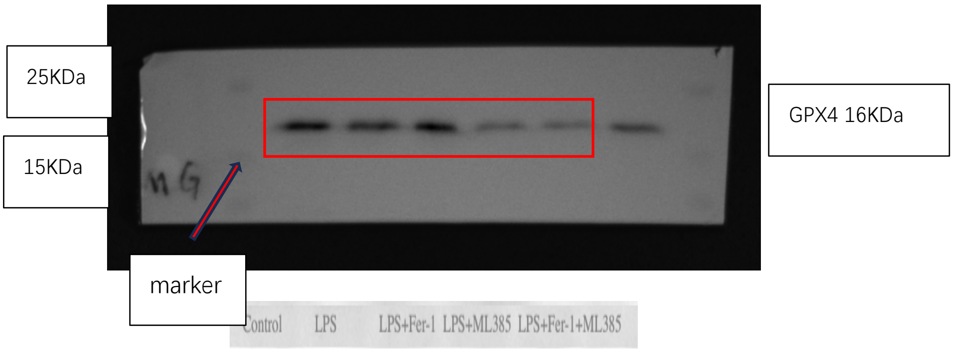

multiple exposure images of x–CT

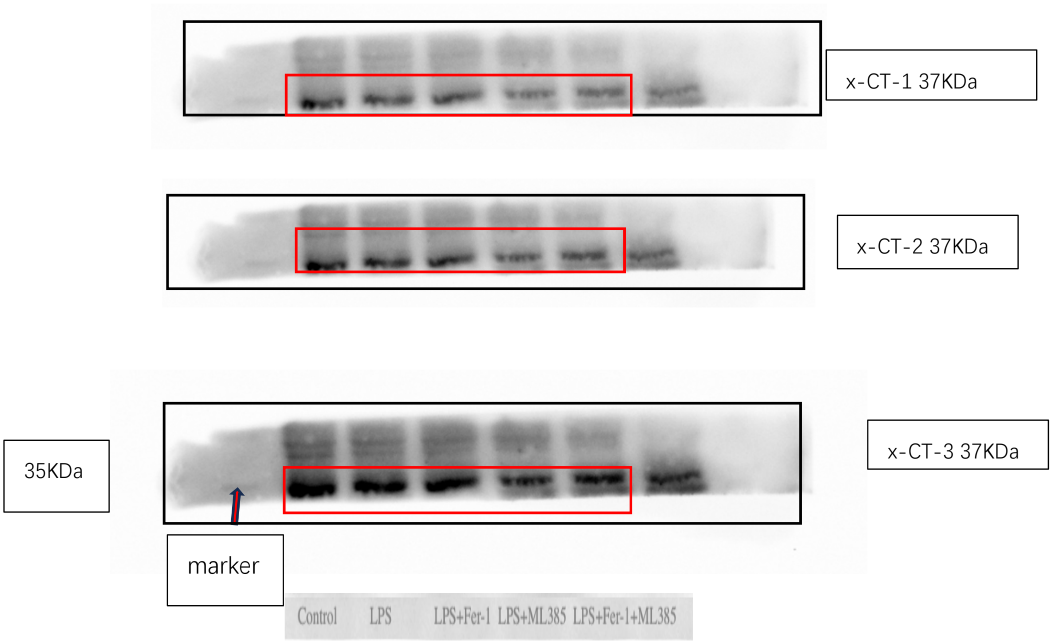

multiple exposure images of PTGS2

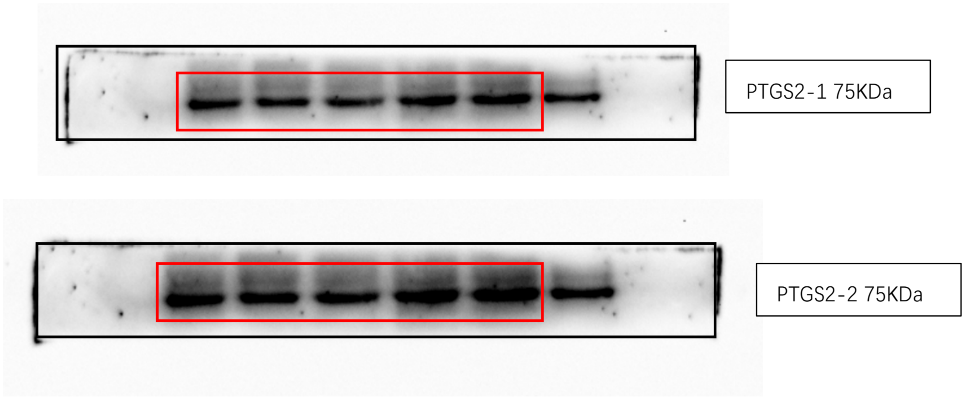

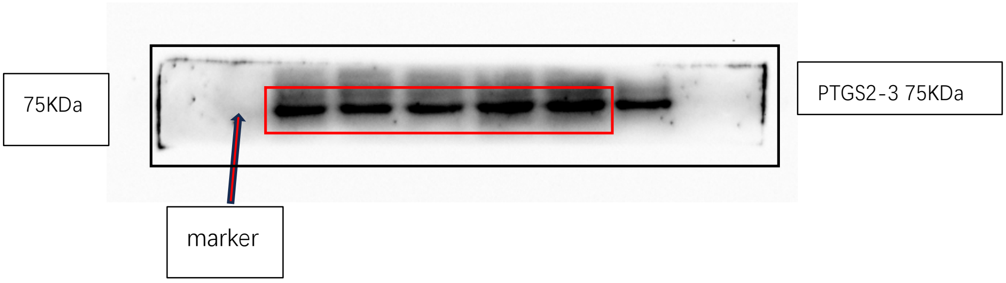

Fig 5F

multiple exposure images of Actin

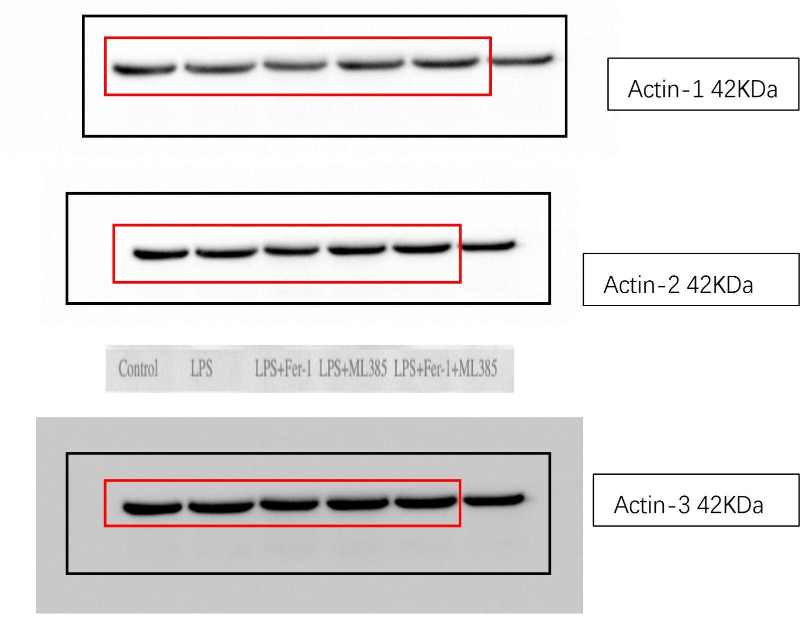

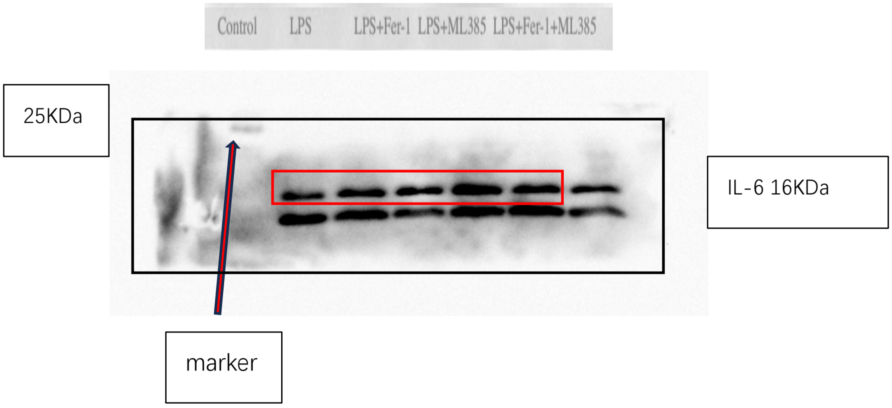

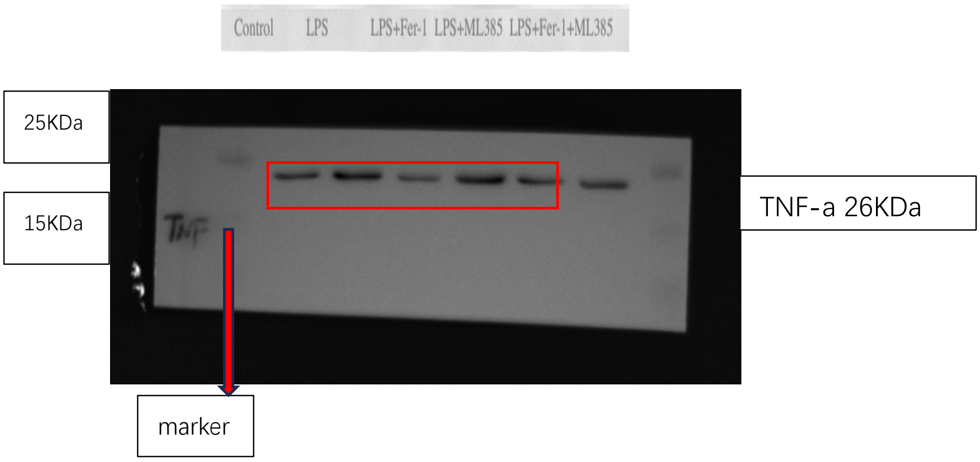

multiple exposure images of HMGB1

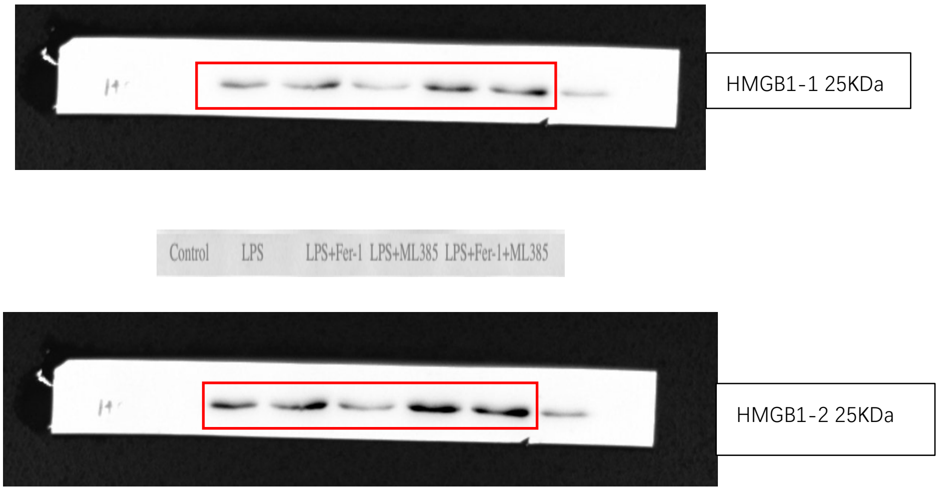

Fig 6A

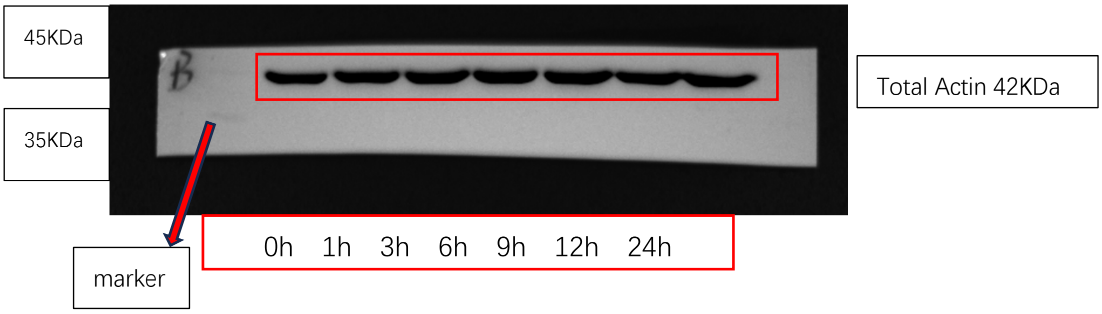

multiple exposure images of Total NRF2

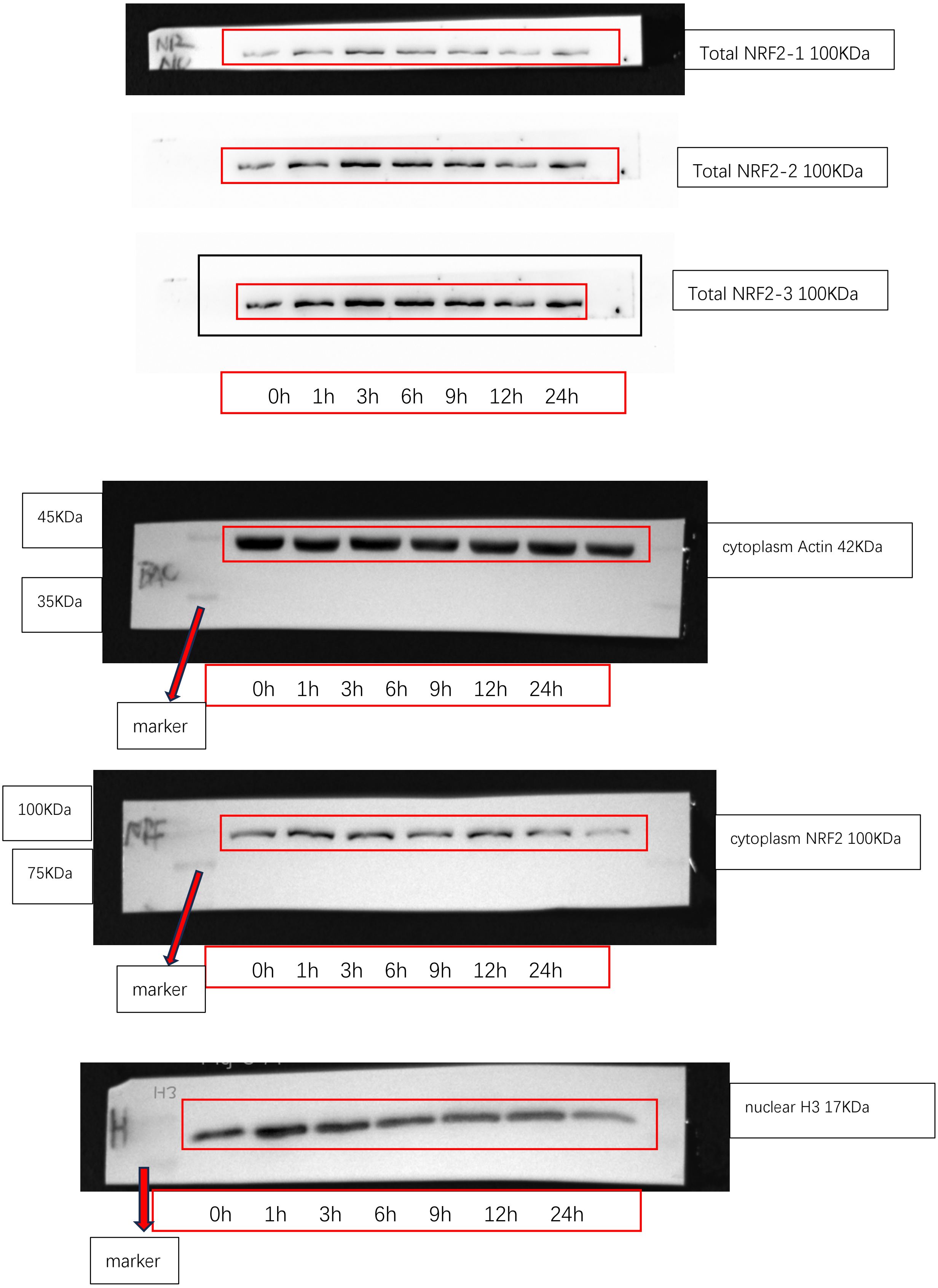

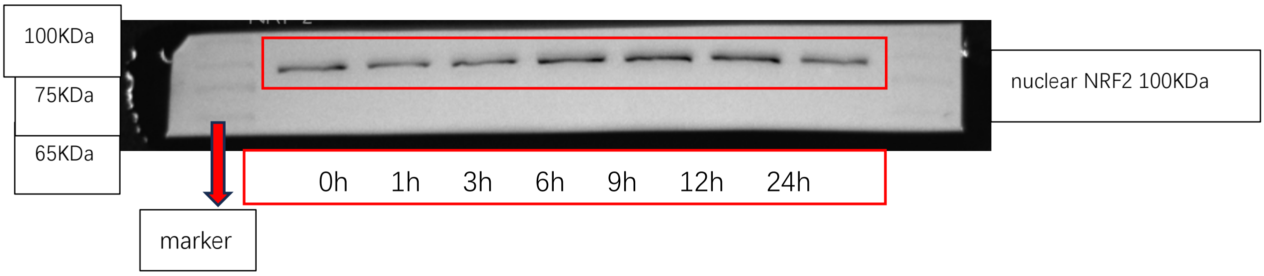

Fig 6C

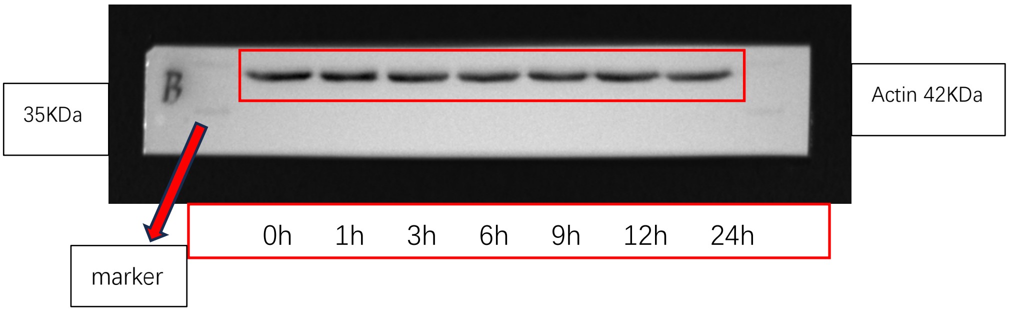

multiple exposure images of Total NOX4

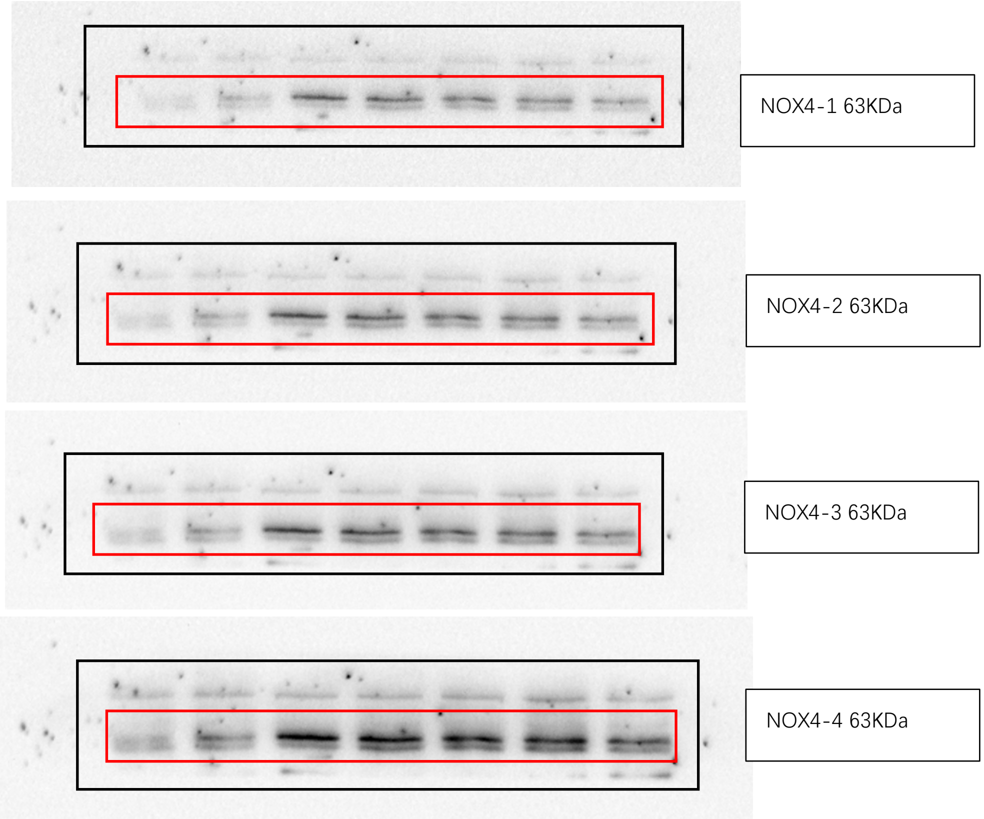

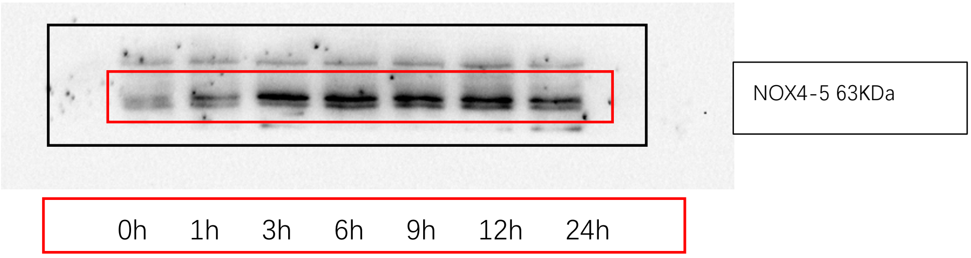

Fig 6E

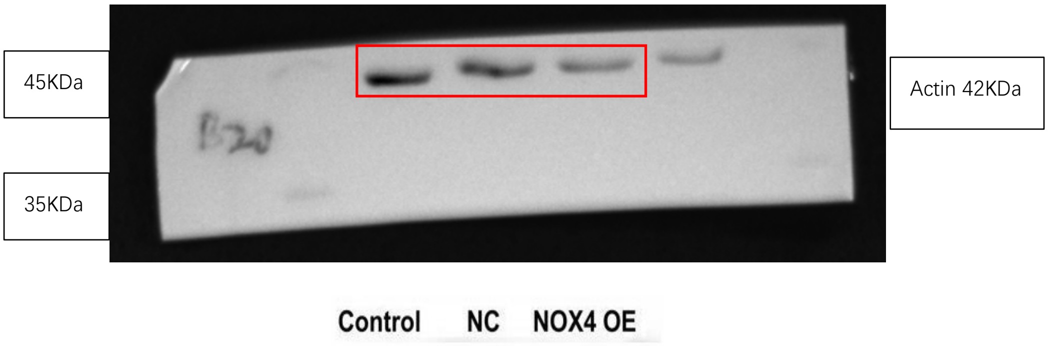

multiple exposure images of NRF2

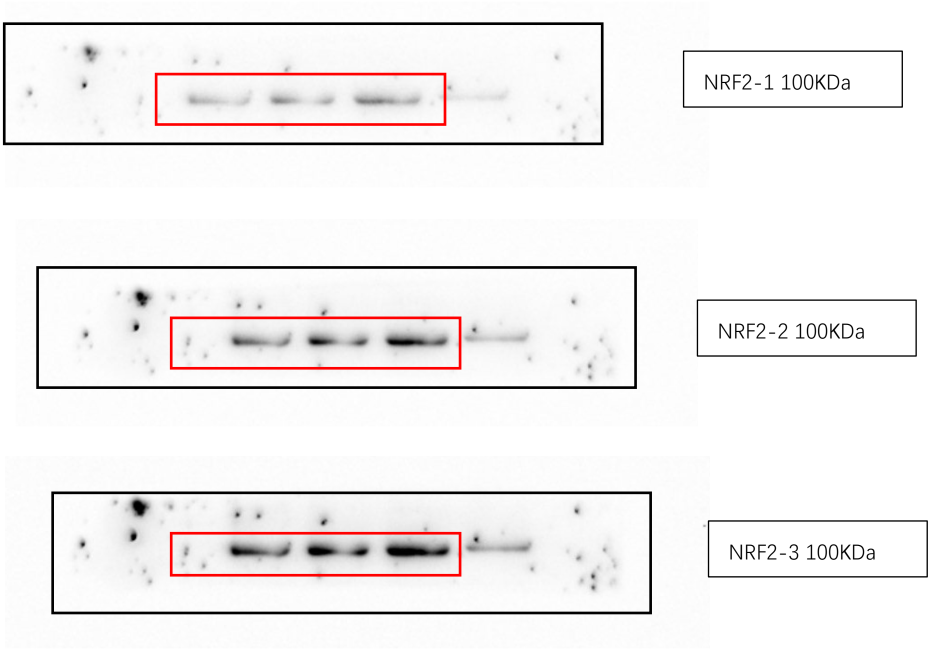

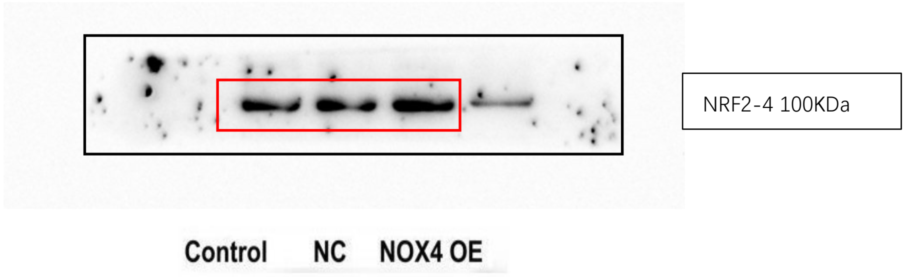

multiple exposure images of NOX4

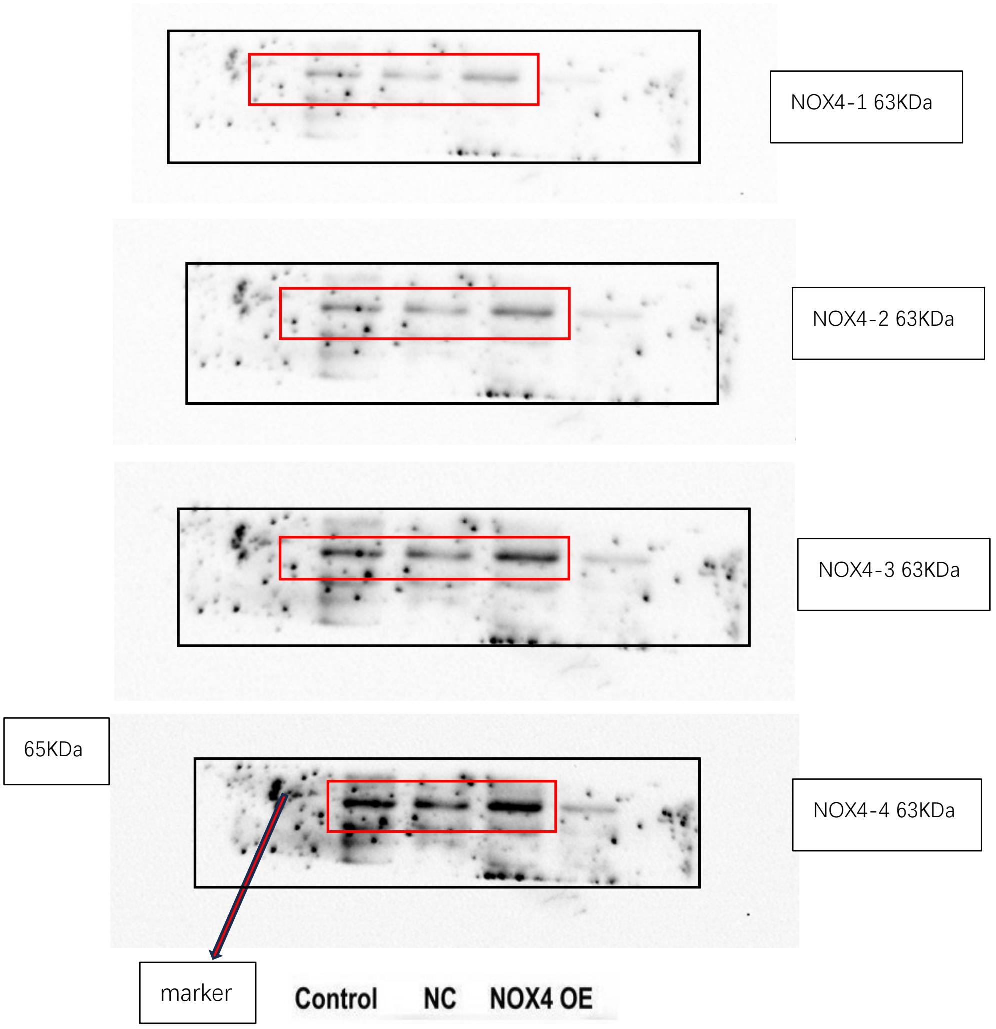

Fig 6G

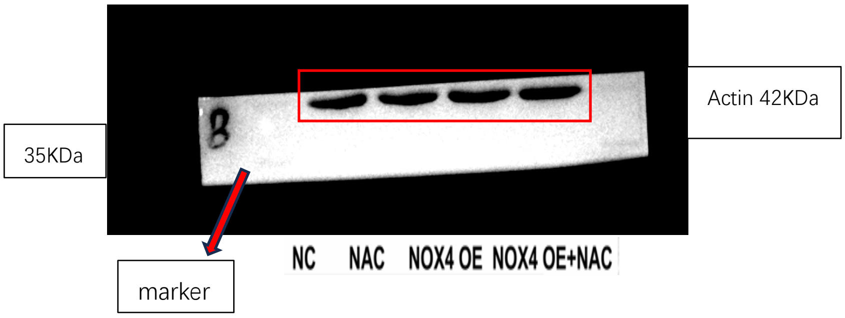

multiple exposure images of NOX4

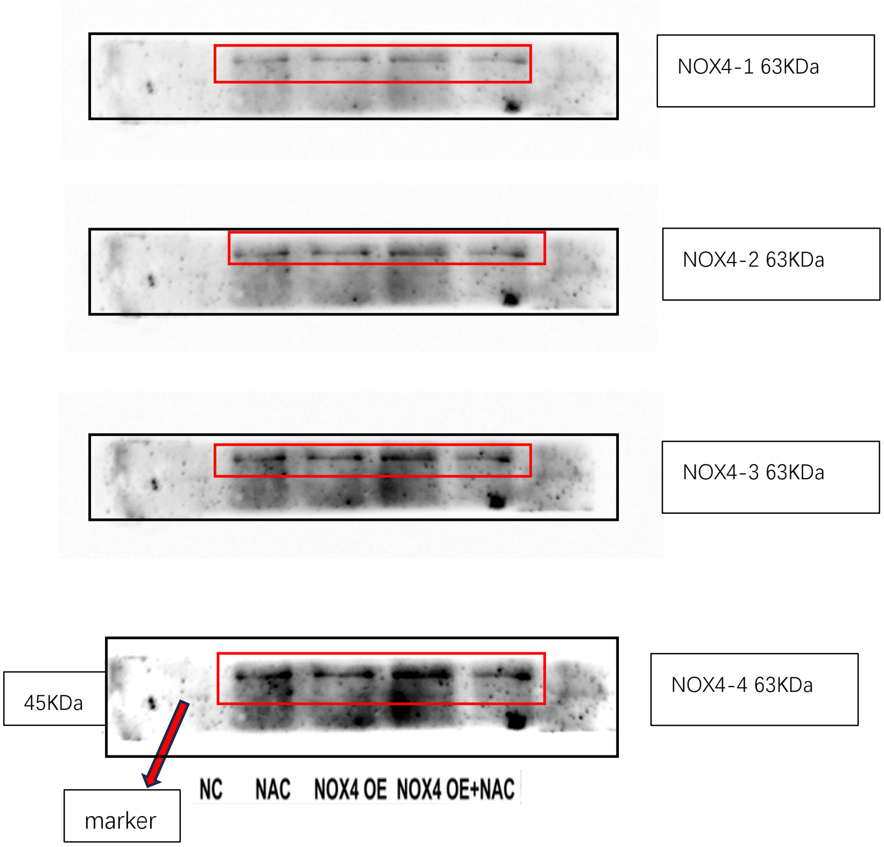

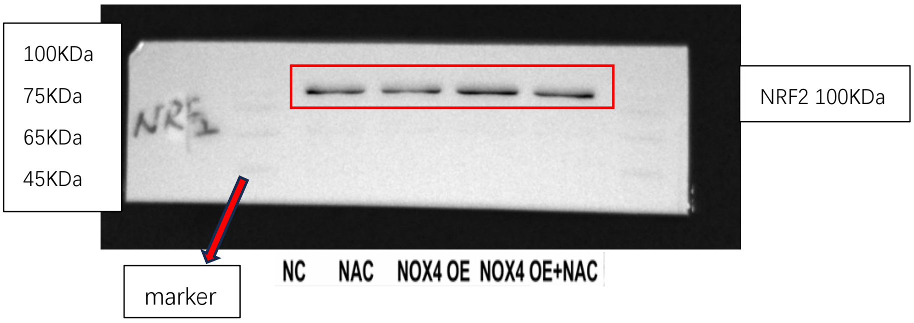

Fig 6H

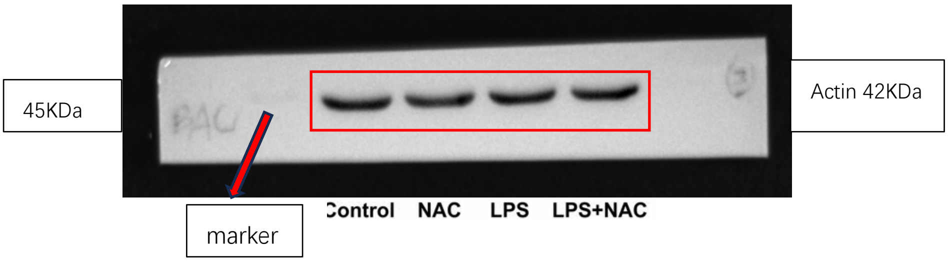

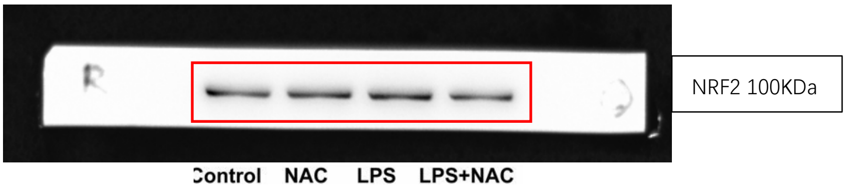

multiple exposure images of NOX4

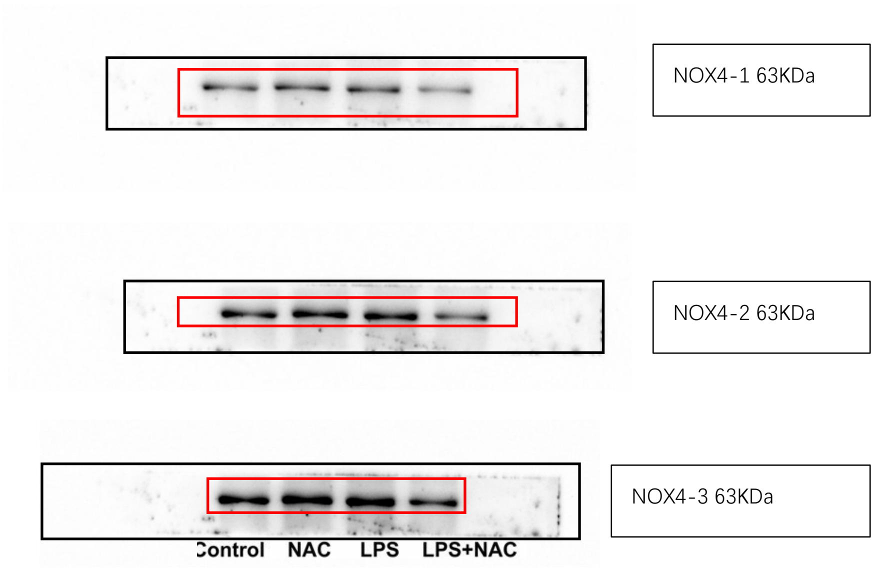

Replicate blots

Fig 2E

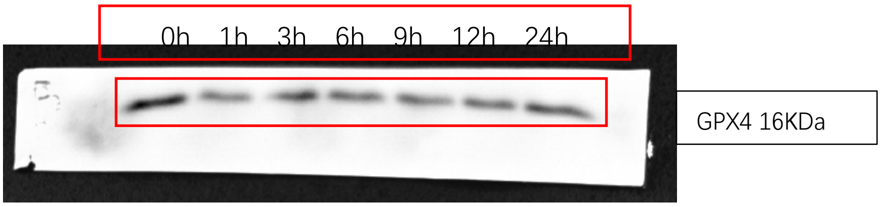

multiple exposure images of GPX4

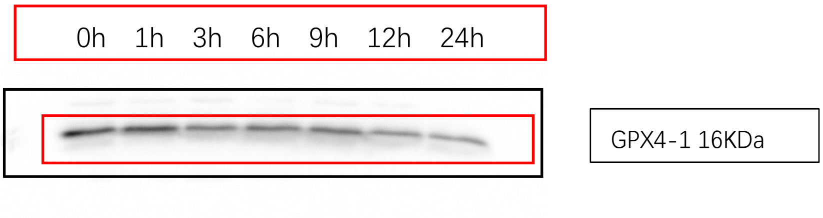

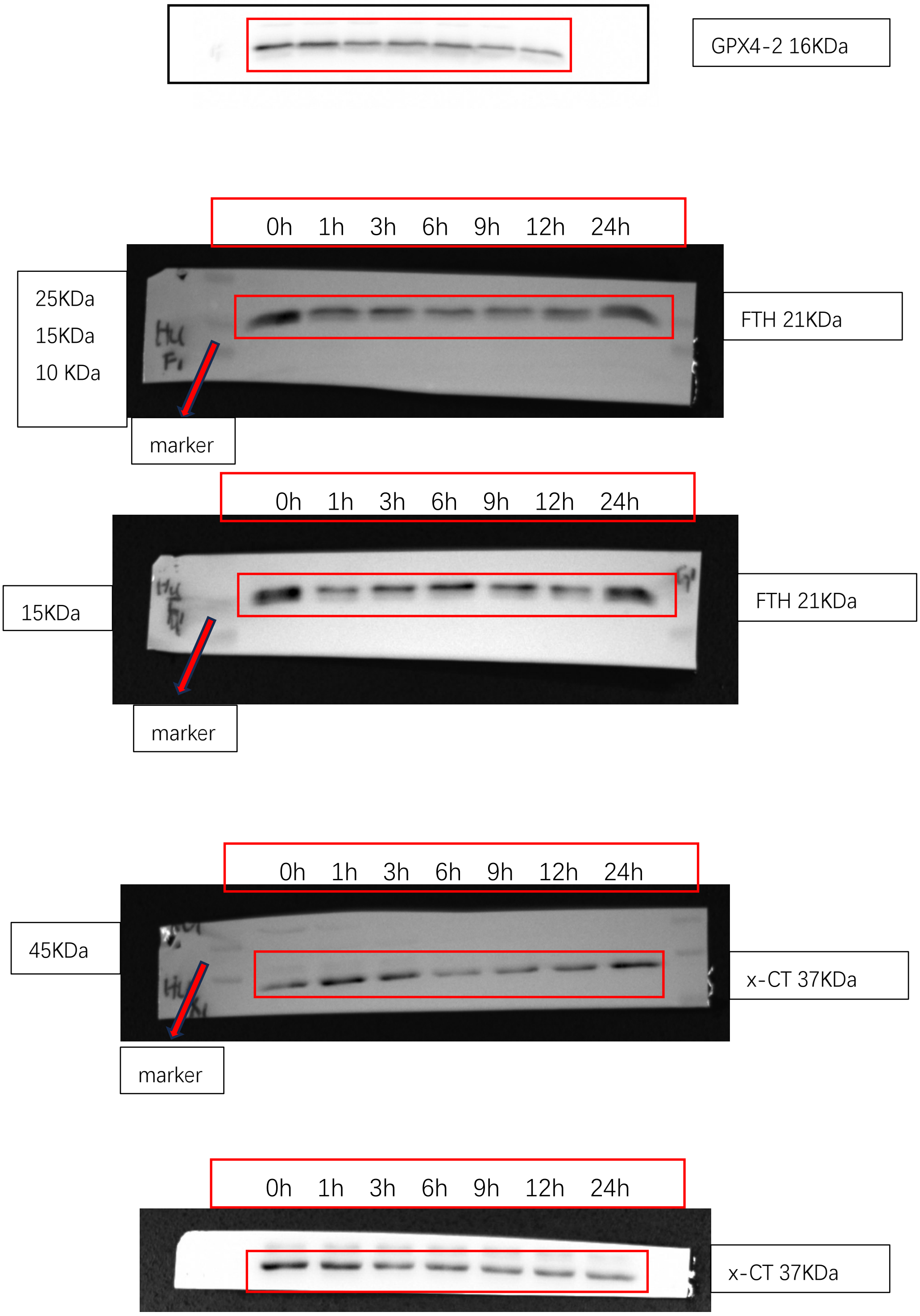
GPX4-2 16KDa
0h 1h 3h 6h 9h 12h 24h
25KDa
15KDa
10 KDa
FTH 21KDa
marker
0h 1h 3h 6h 9h 12h 24h
15KDa
FTH 21KDa
marker
0h 1h 3h 6h 9h 12h 24h
45KDa
x-CT 37KDa
marker
0h 1h 3h 6h 9h 12h 24h
x-CT 37KDa

multiple exposure images of NCOA4

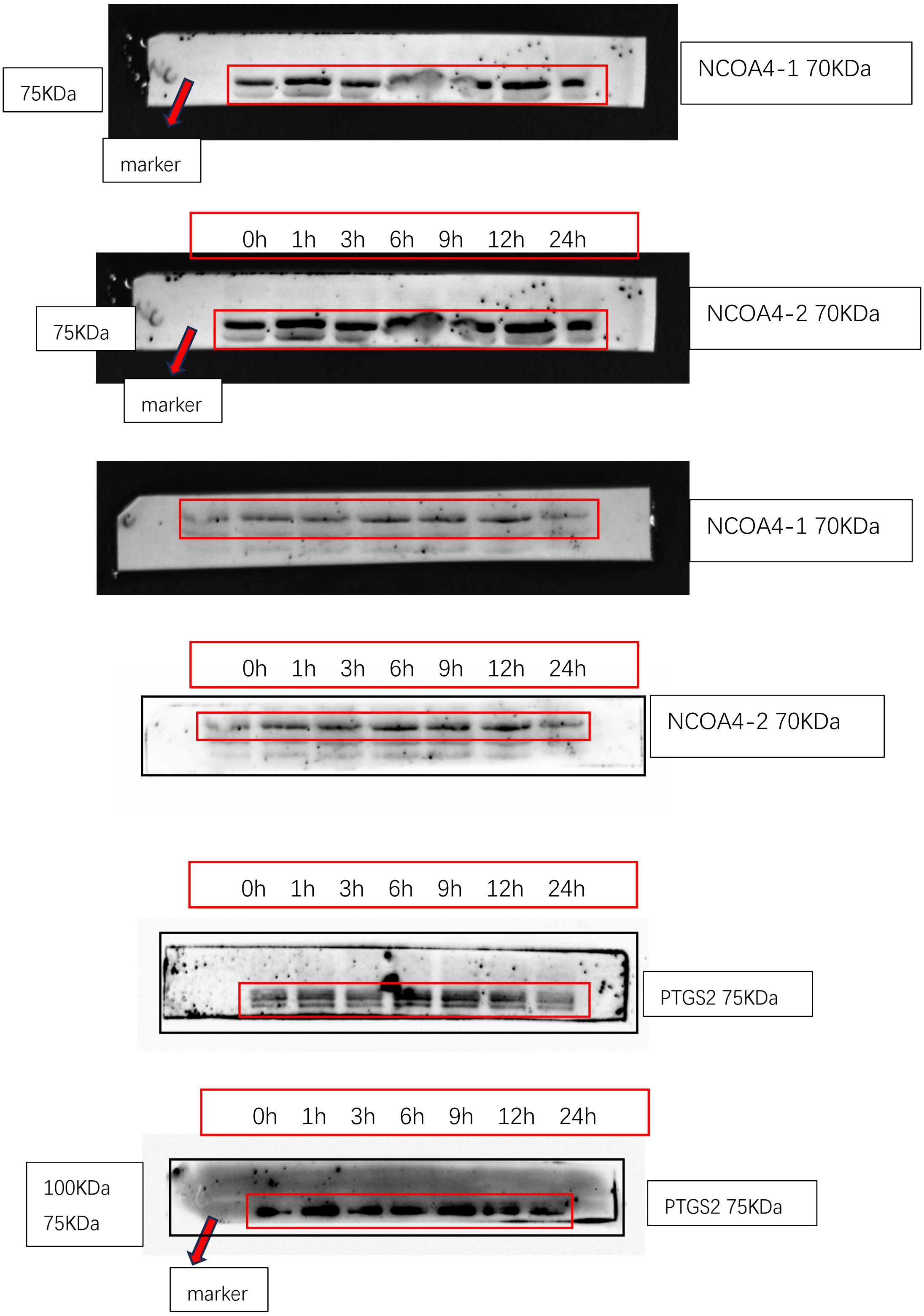

Fig 2B

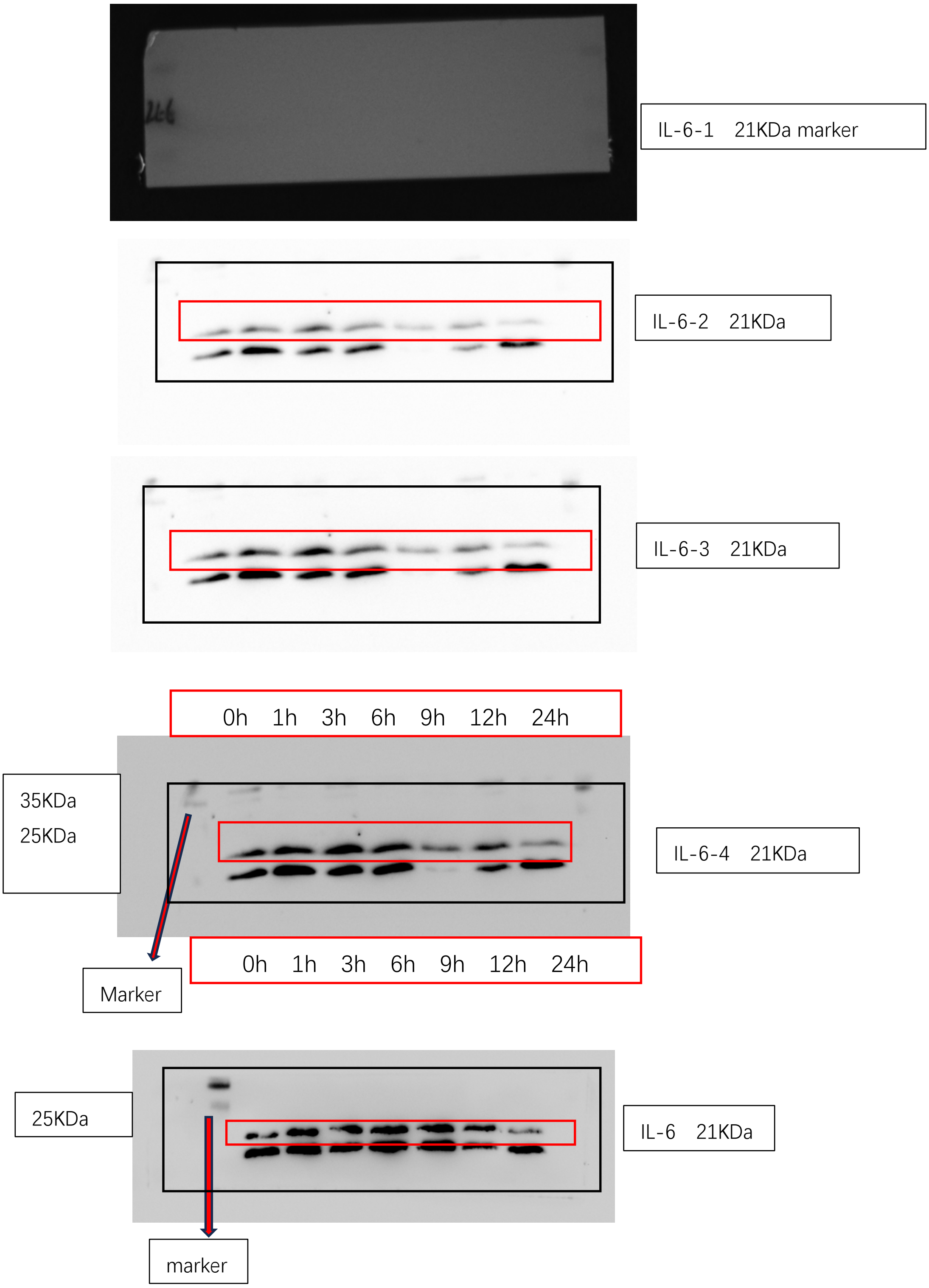
IL-6-1 21KDa marker
IL-6-2 21KDa
IL-6-3 21KDa
0h 1h 3h 6h 9h 12h 24h
35KDa
25KDa
IL-6-4 21KDa
0h 1h 3h 6h 9h 12h 24h
Marker
25KDa
IL-6 21KDa
marker

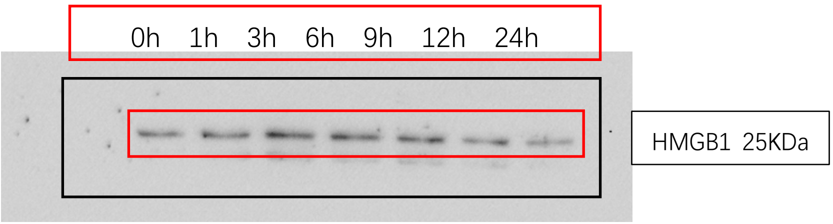

multiple exposure images of HMGB1

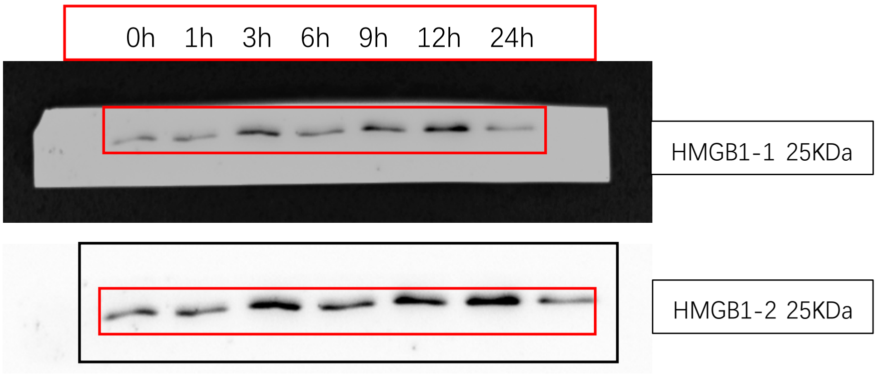

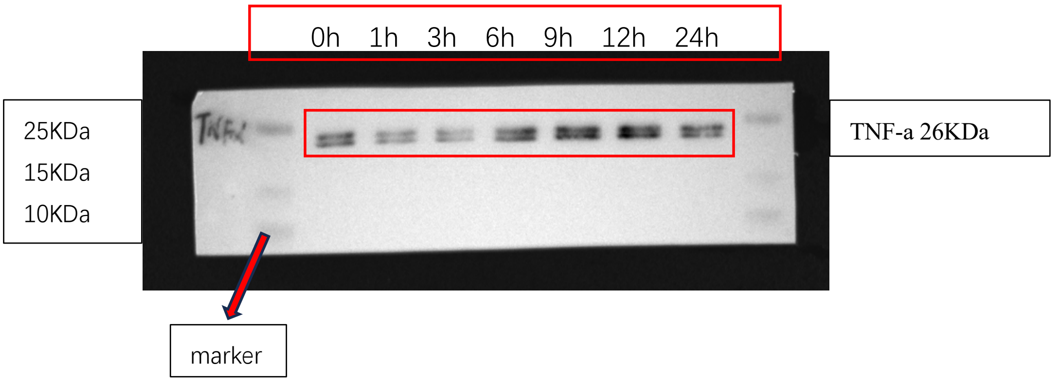

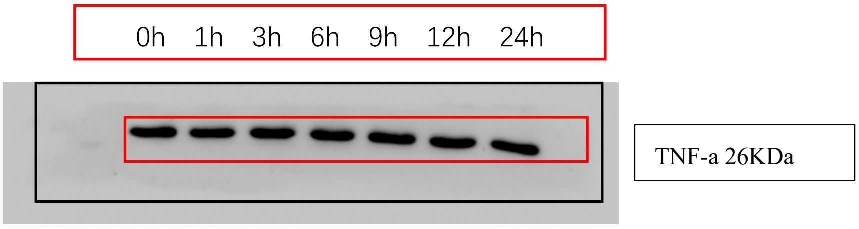

Fig 3E

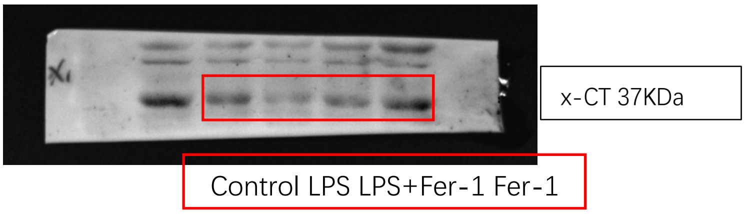

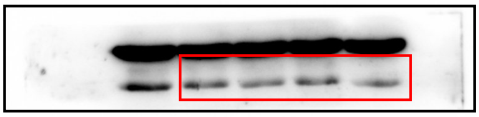

x-CT 37KDa

Control LPS LPS+Fer-1 Fer-1

10KDa

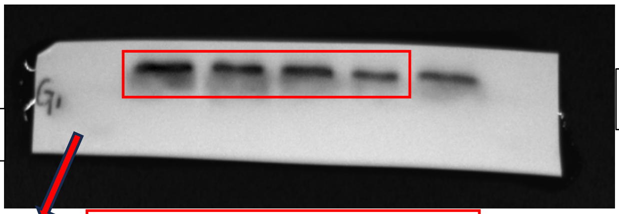

GPX4 16KDa

marker

Control LPS LPS+Fer-1 Fer-1

Control LPS LPS+Fer-1 Fer-1

10KDa

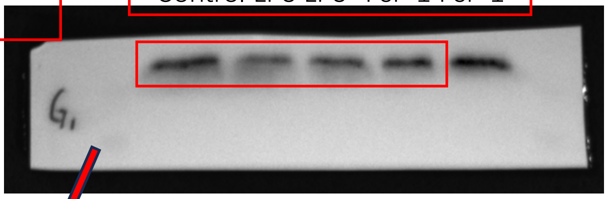

GPX4 16KDa

marker

Control LPS LPS+Fer-1 Fer-1

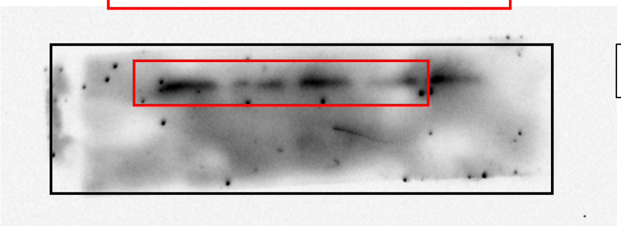

FTH 21KDa

Control LPS LPS+Fer-1 Fer-1

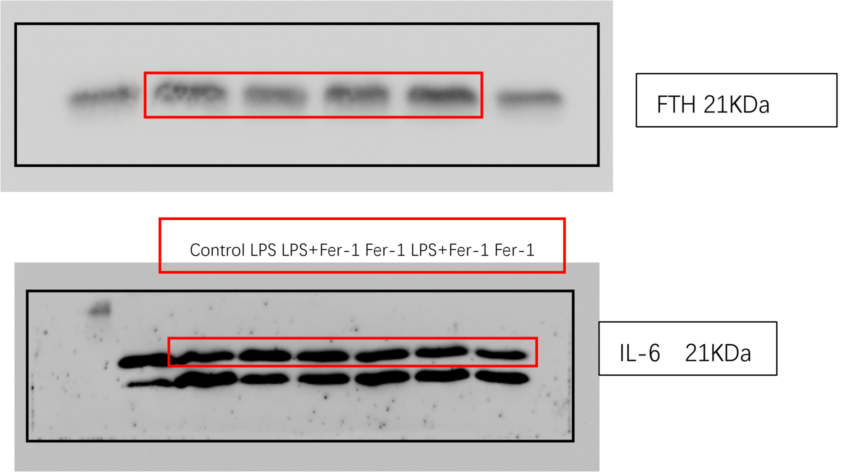

multiple exposure images of HMGB

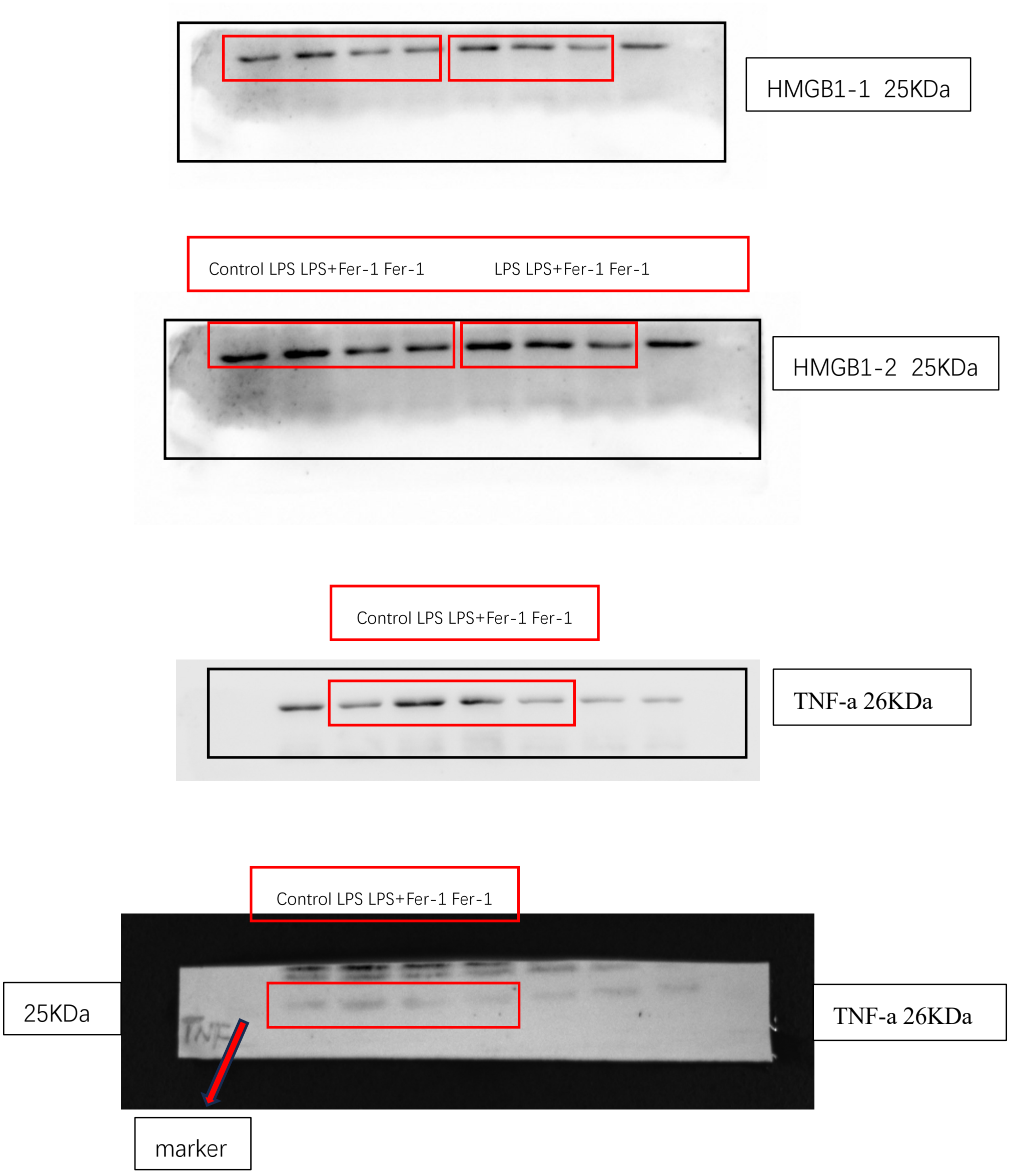

Fig 4B

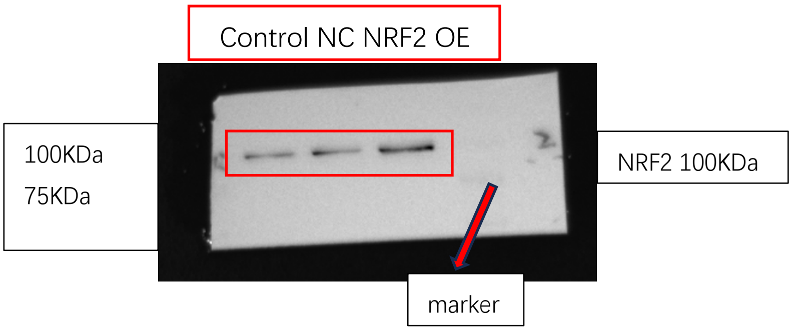

multiple exposure images of NRF2

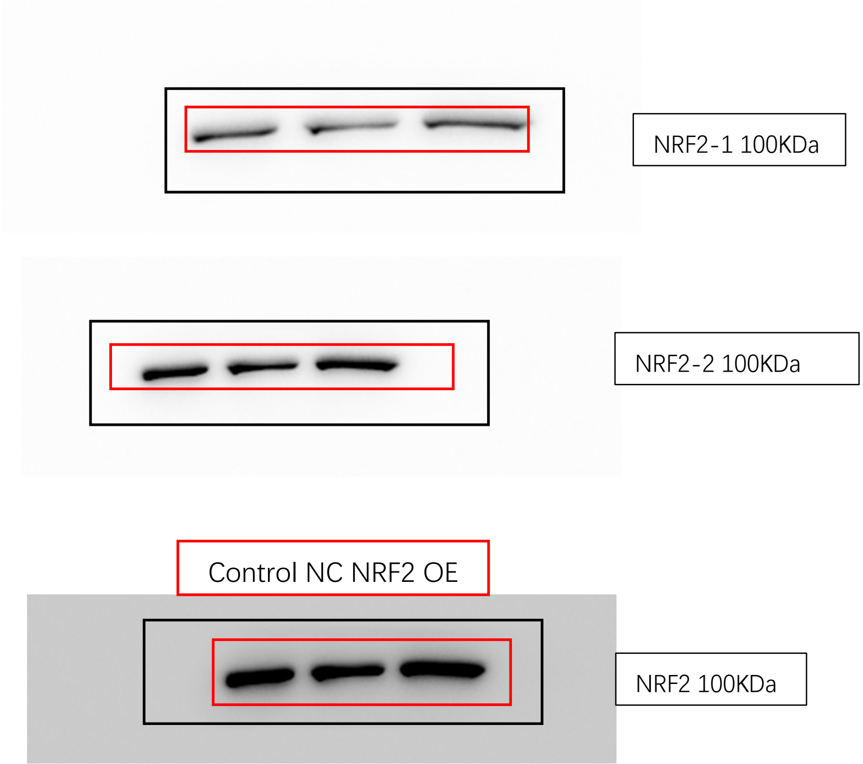

Fig 4F

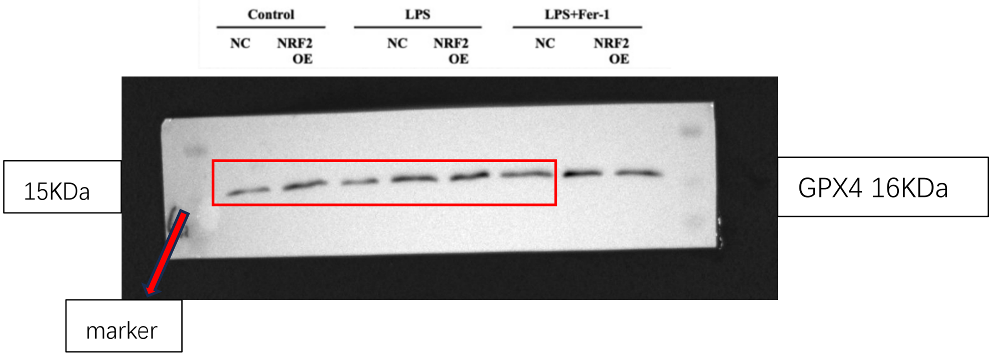

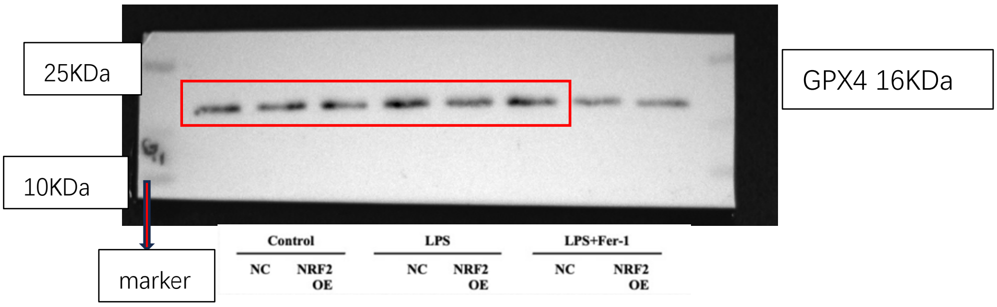
25KDa
10KDa
marker
GPX4 16KDa
Control
LPS
LPS+Fer-1
NC
NRF2 OE
NC
NRF2 OE
NC
NRF2 OE

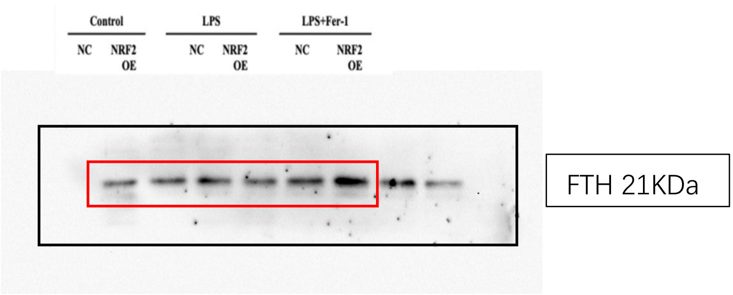
Control
LPS
LPS+Fer-1
NC
NRF2 OE
NC
NRF2 OE
NC
NRF2 OE
FTH 21KDa

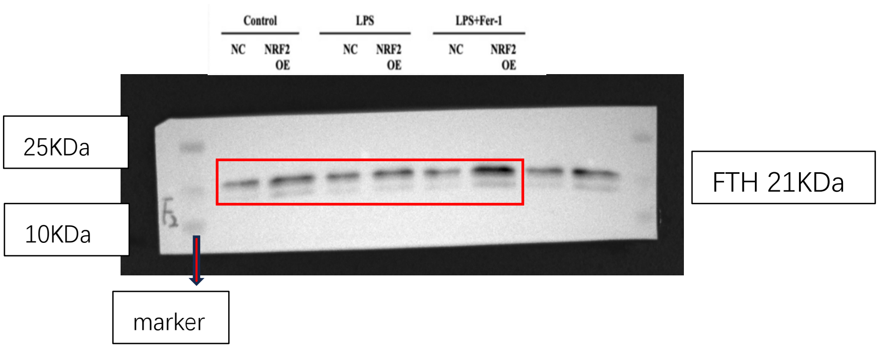
Control
LPS
LPS+Fer-1
NC
NRF2 OE
NC
NRF2 OE
NC
NRF2 OE
25KDa
10KDa
marker
FTH 21KDa

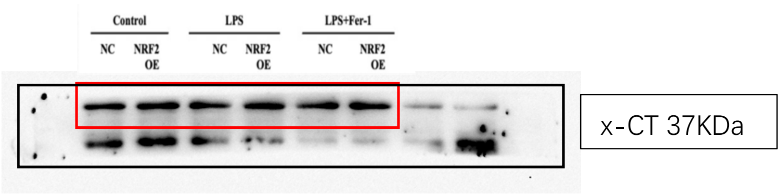
Control
LPS
LPS+Fer-1
NC
NRF2 OE
NC
NRF2 OE
NC
NRF2 OE
x-CT 37KDa

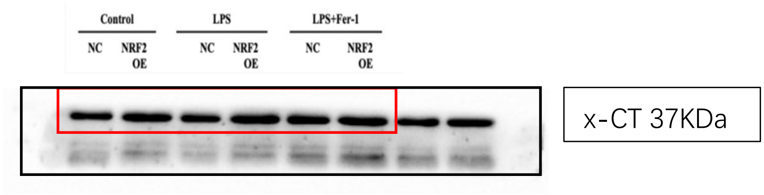
Control
LPS
LPS+Fer-1
NC
NRF2 OE
NC
NRF2 OE
NC
NRF2 OE
x-CT 37KDa

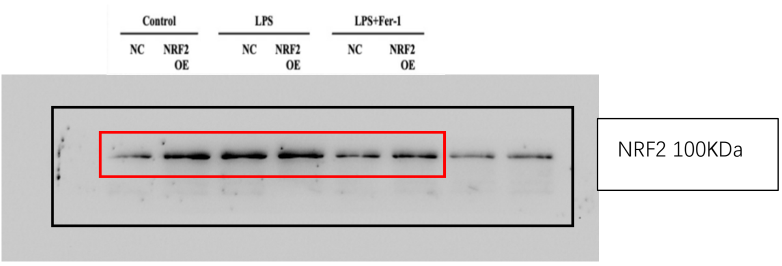
Control
LPS
LPS+Fer-1
NC
NRF2 OE
NC
NRF2 OE
NC
NRF2 OE
NRF2 100KDa

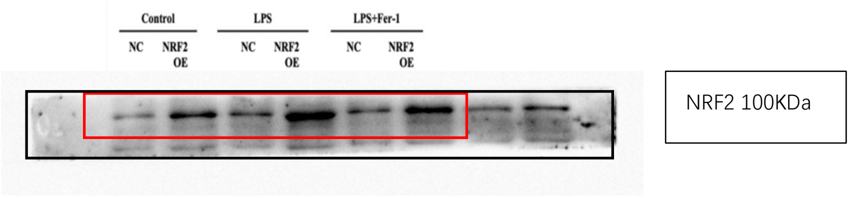

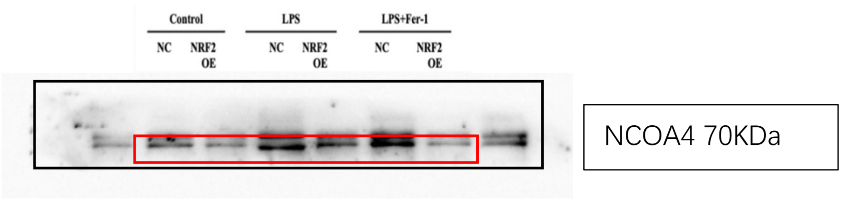

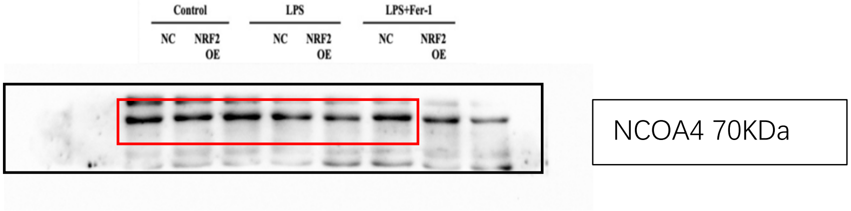

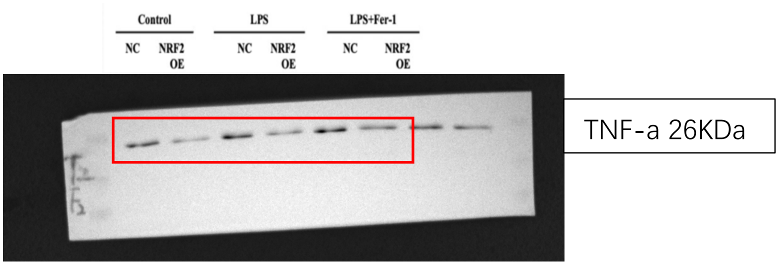

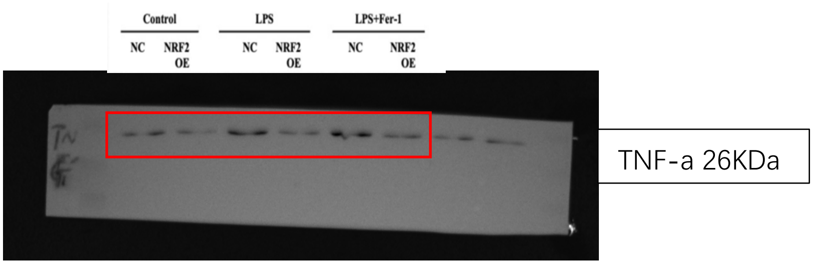

multiple exposure images of PTGS2

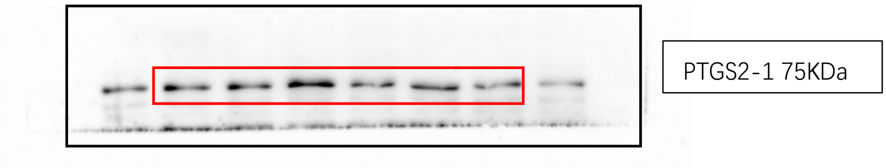

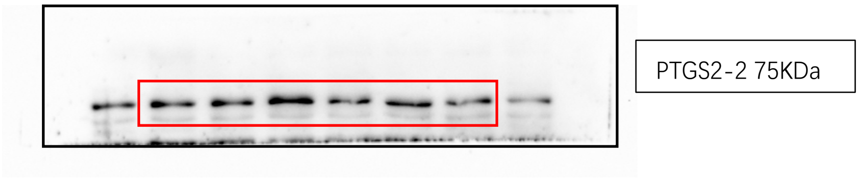

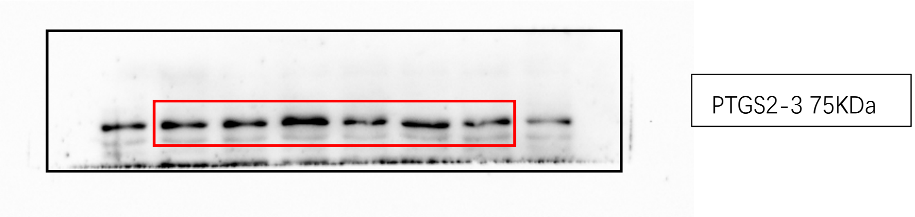

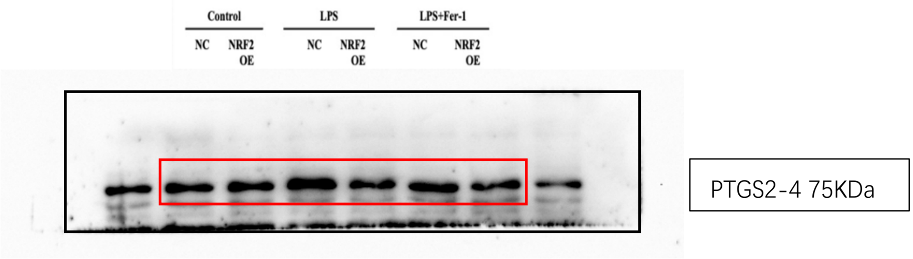

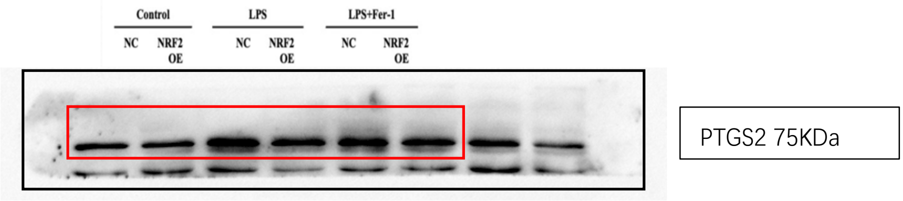

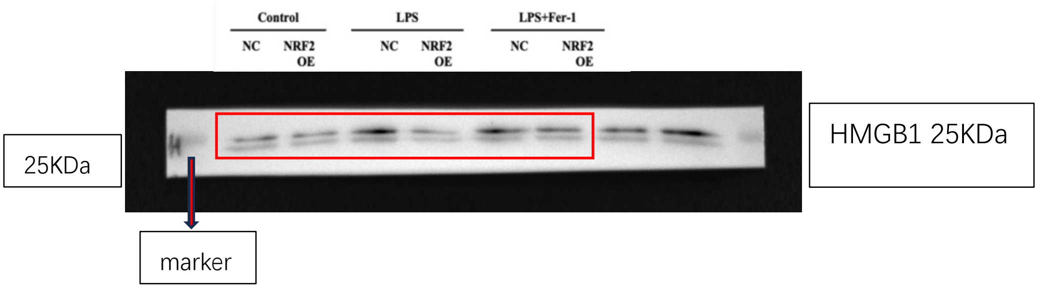

multiple exposure images of HMGB1

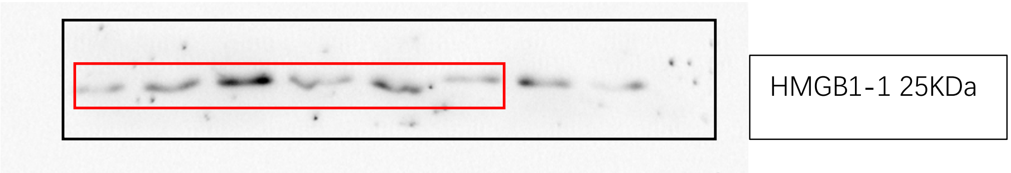

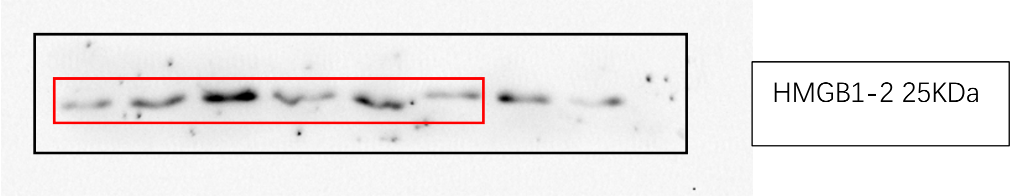

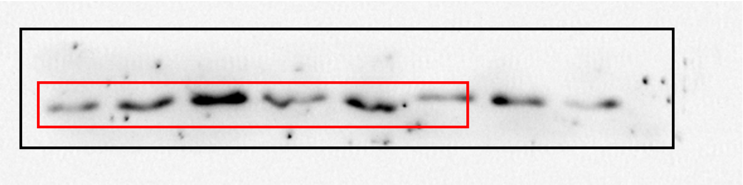

HMGB1-3 25KDa

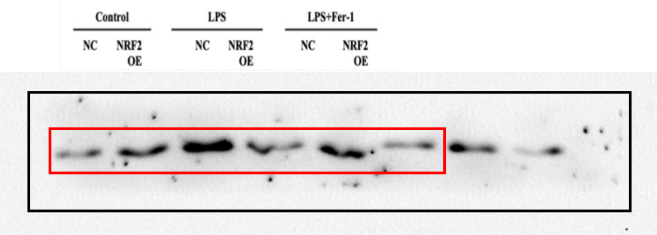

HMGB1-4 25KDa

multiple exposure images of HMGB1

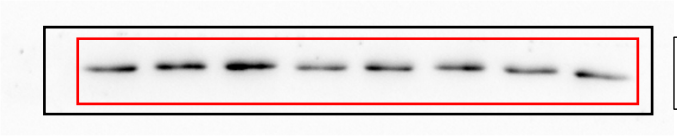

IL-6-1 16KDa

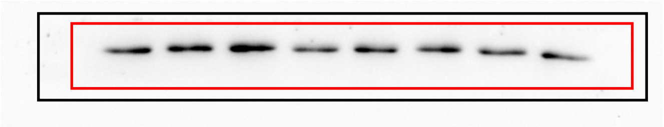

IL-6-2 16KDa

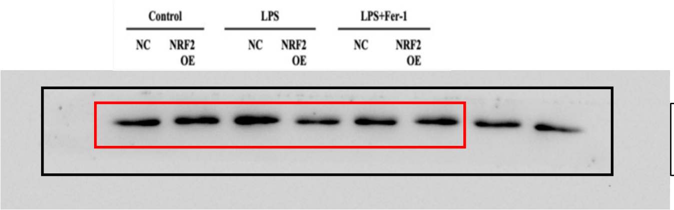

IL-6-3 16KDa

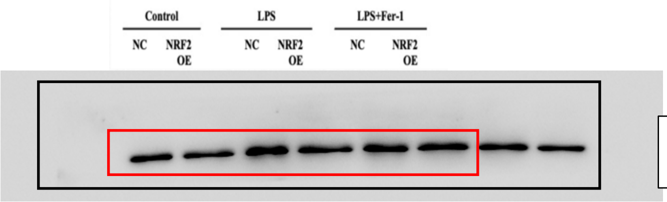

IL-6 16KDa

Fig 5A

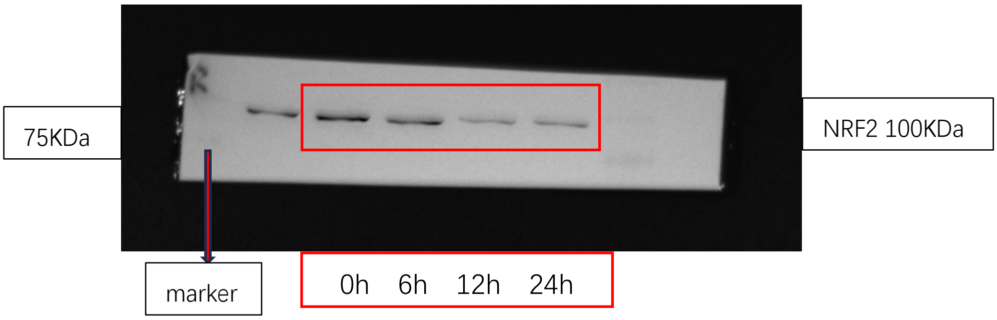

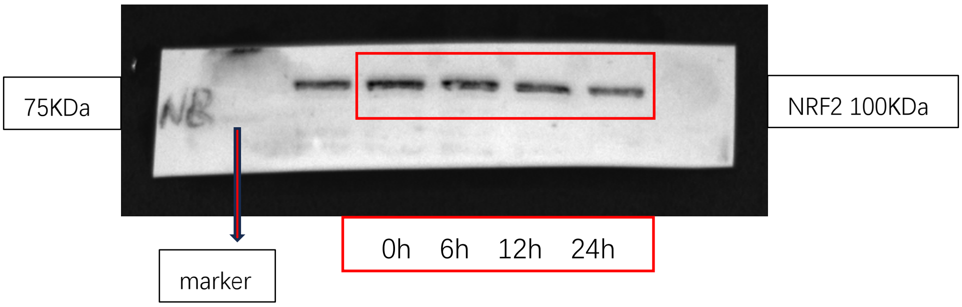

Fig 5E

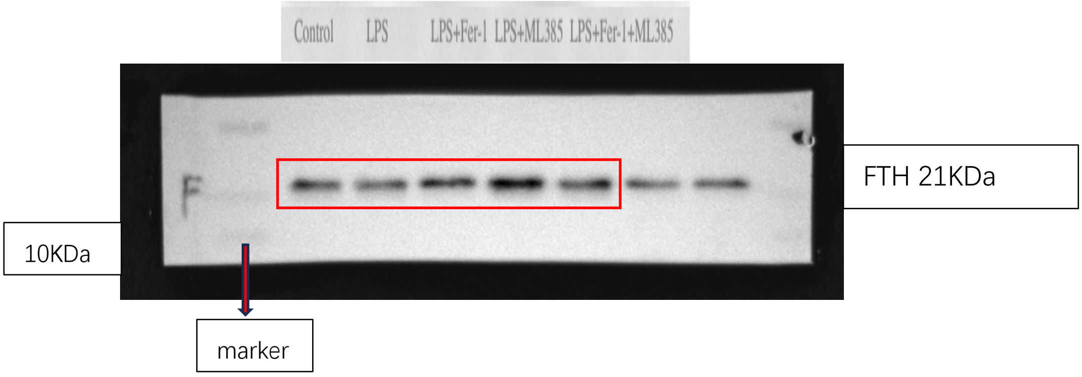

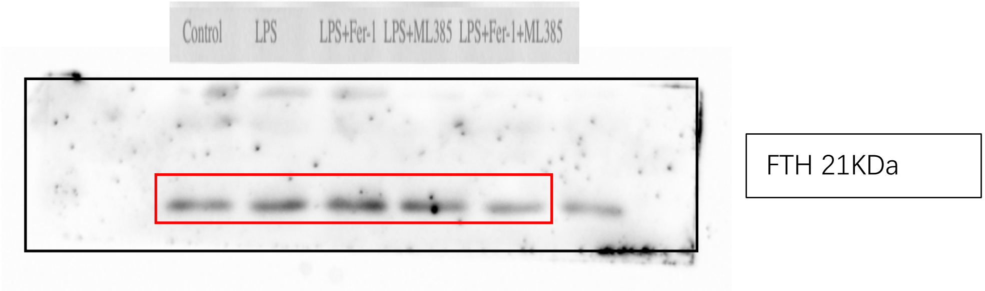

multiple exposure images of x–CT

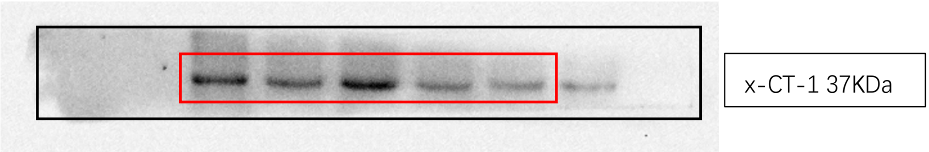

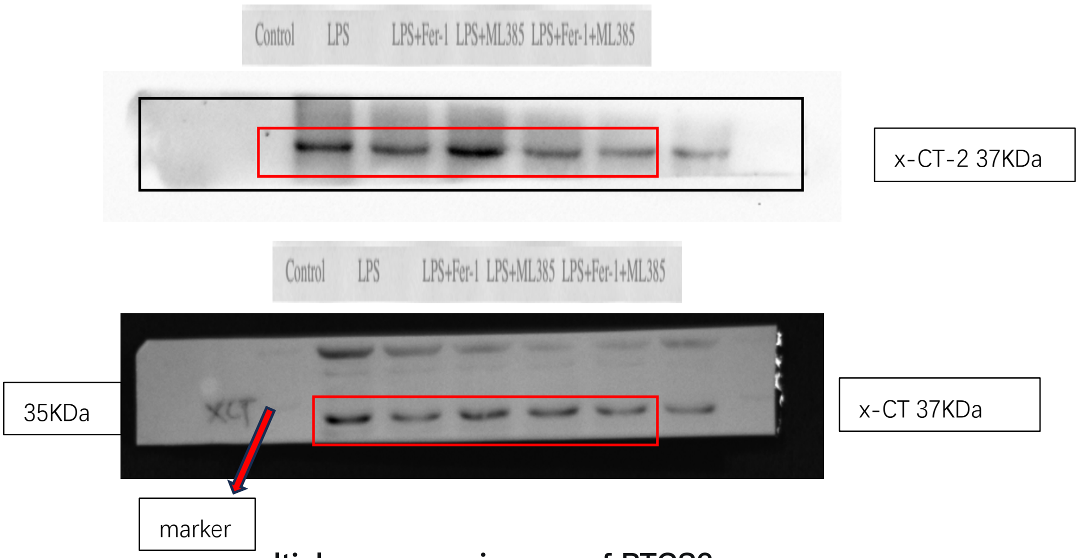

multiple exposure images of PTGS2

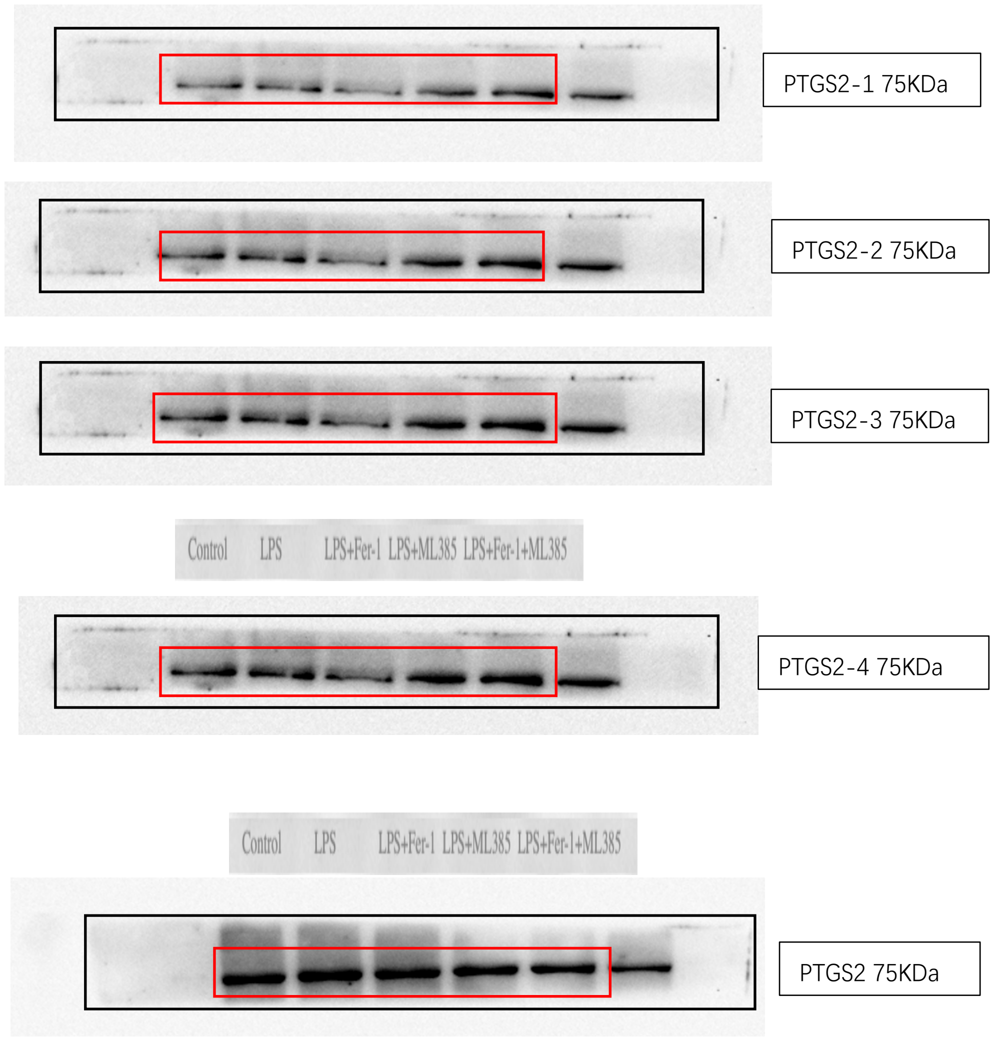

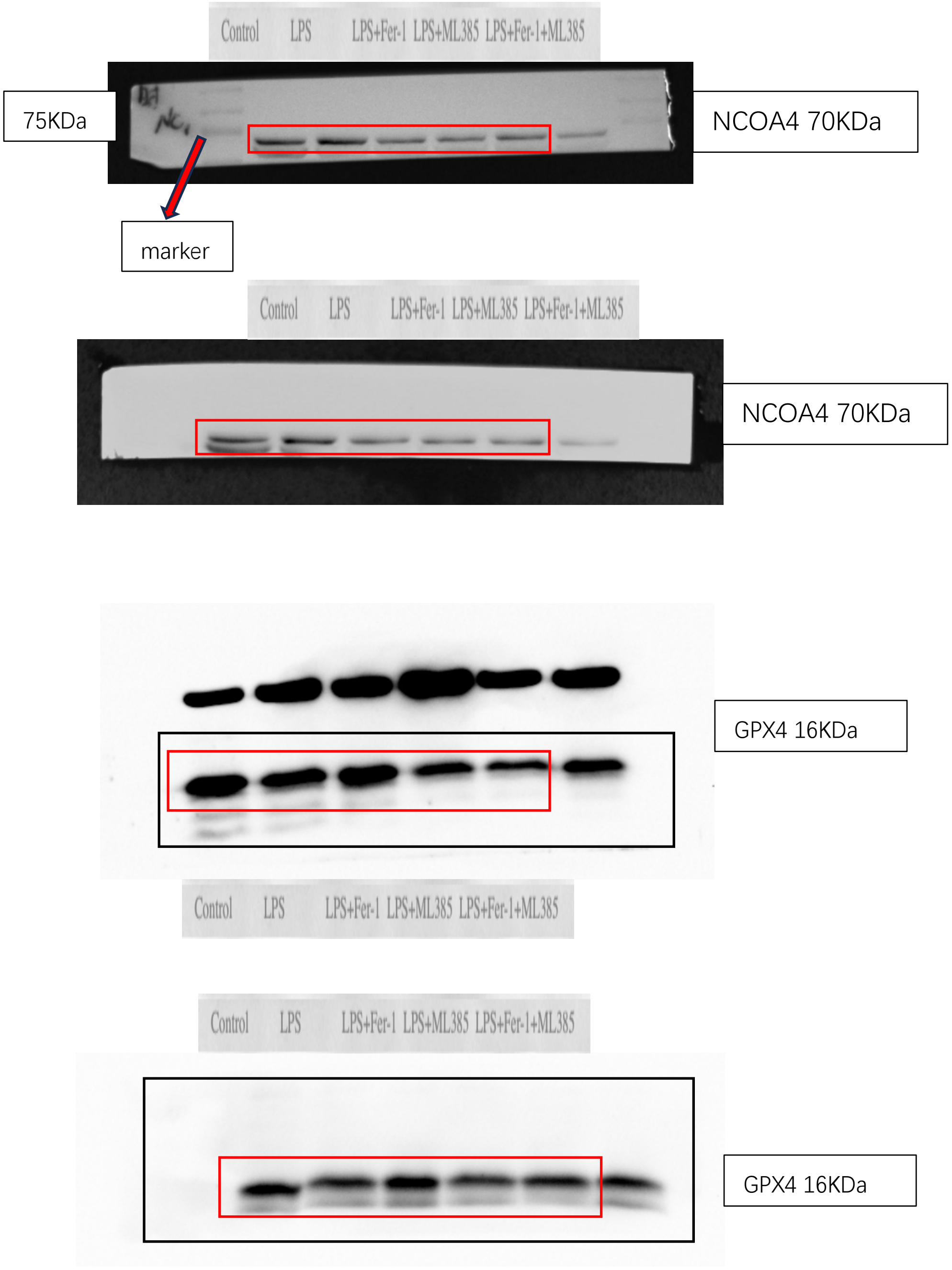
Control LPS LPS+Fer-1 LPS+ML385 LPS+Fer-1+ML385
75KDa
NCOA4 70KDa
marker
Control LPS LPS+Fer-1 LPS+ML385 LPS+Fer-1+ML385
NCOA4 70KDa
GPX4 16KDa
Control LPS LPS+Fer-1 LPS+ML385 LPS+Fer-1+ML385
Control LPS LPS+Fer-1 LPS+ML385 LPS+Fer-1+ML385
GPX4 16KDa

Fig 5F

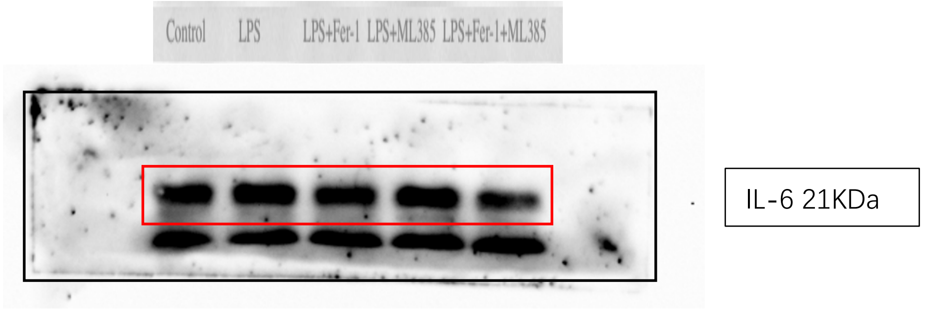
Control LPS LPS+Fer-1 LPS+ML385 LPS+Fer-1+ML385
IL-6 21KDa

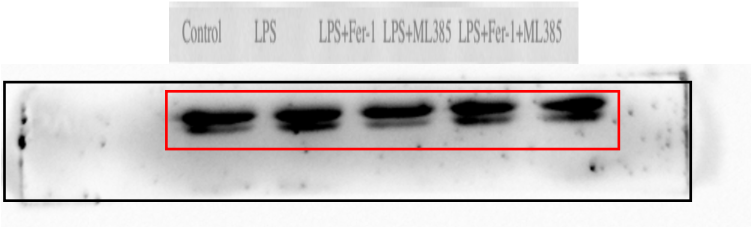

IL-6 21KDa

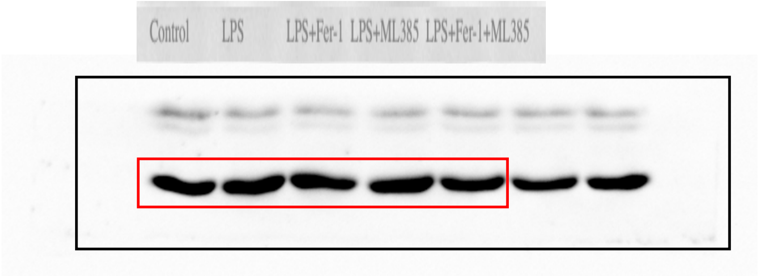

TNF-a 26KDa

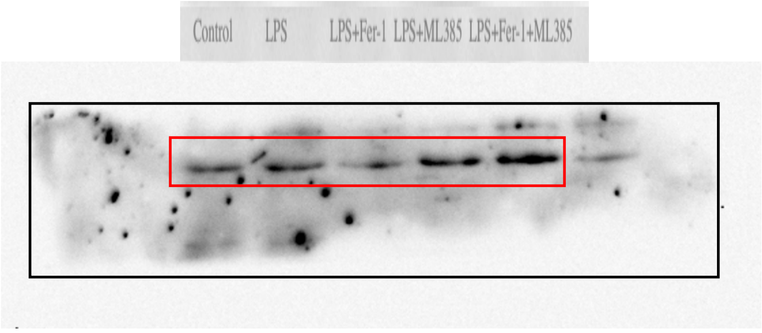

TNF-a 26KDa

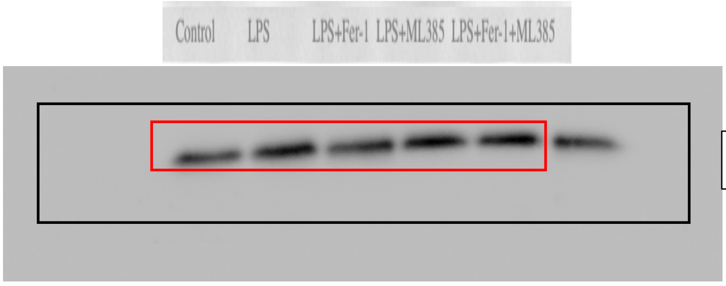

HMGB1 25KDa

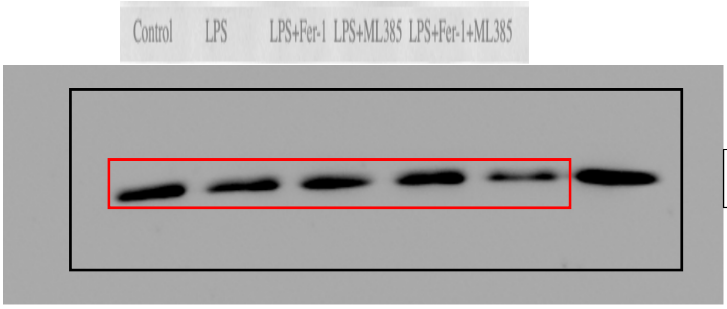

HMGB1 25KDa

Fig 6A

multiple exposure images of PTGS2

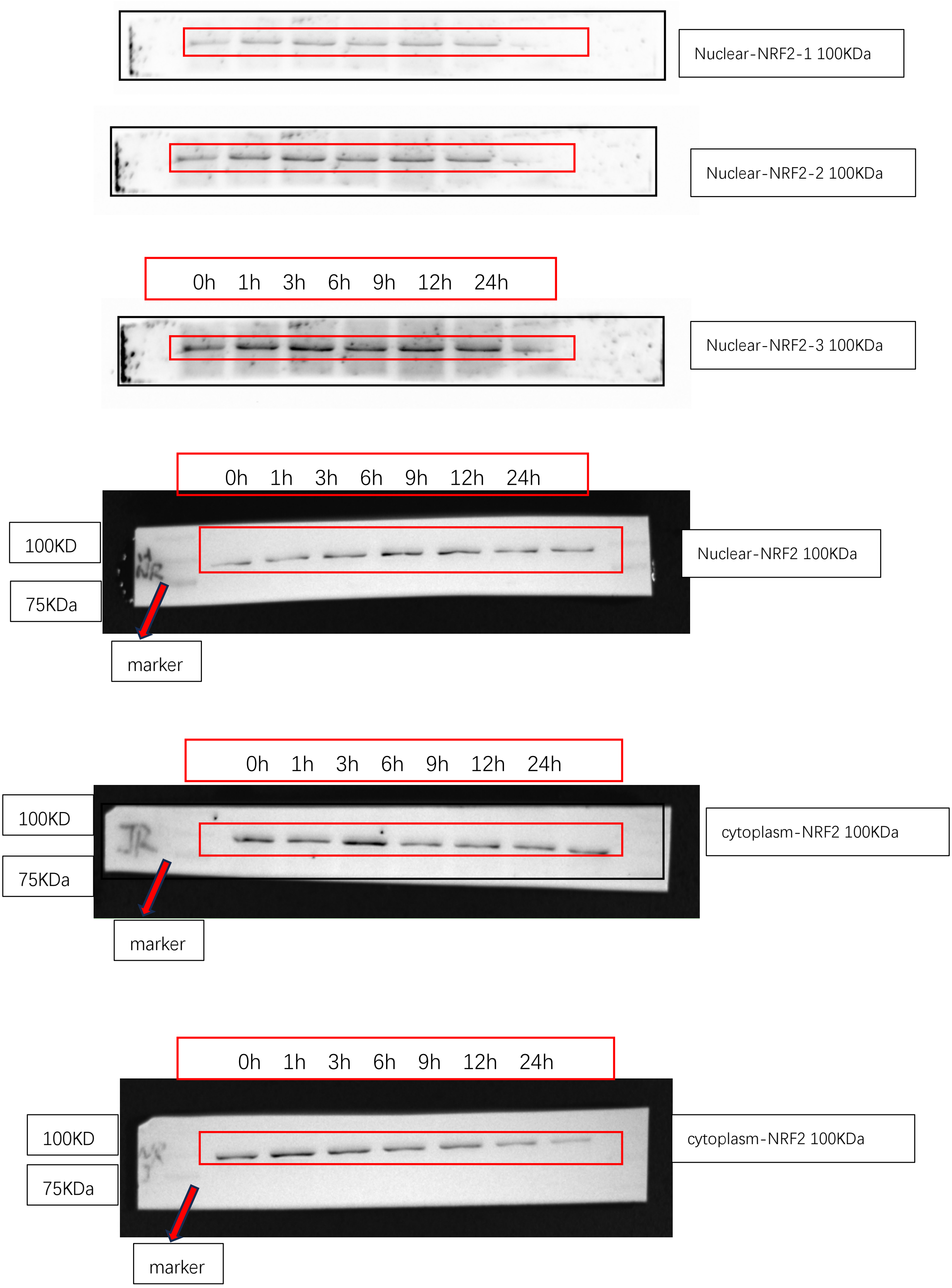

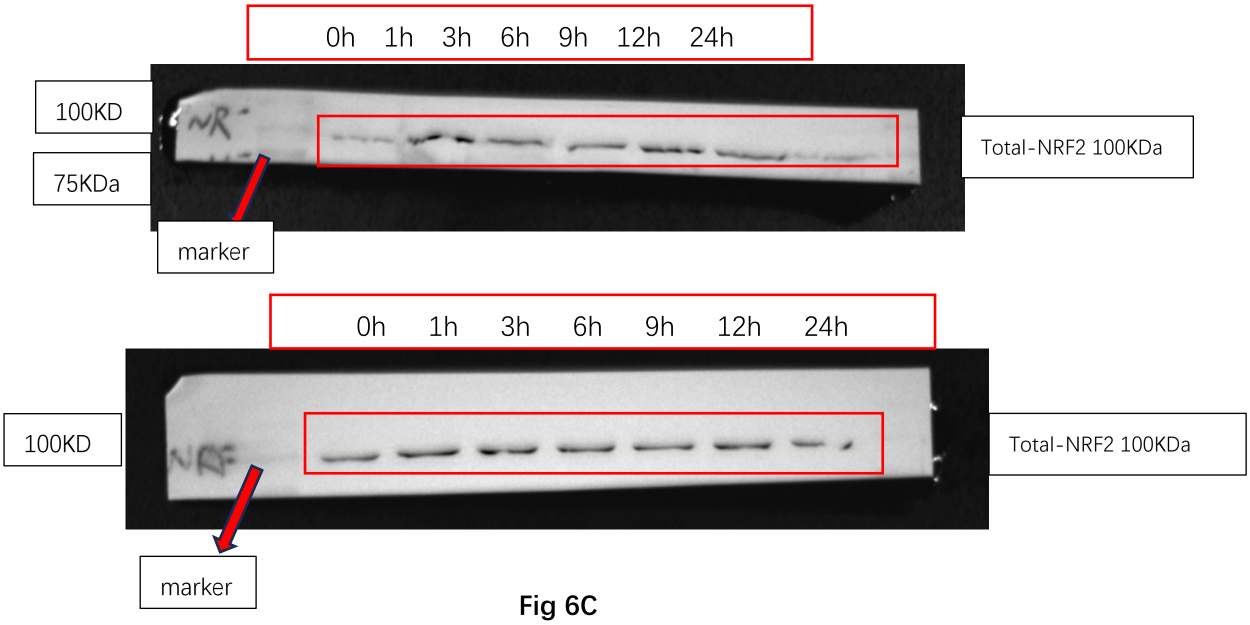
0h 1h 3h 6h 9h 12h 24h
100KD
75KDa
Total-NRF2 100KDa
marker
0h 1h 3h 6h 9h 12h 24h
100KD
Total-NRF2 100KDa
marker

Fig 6C

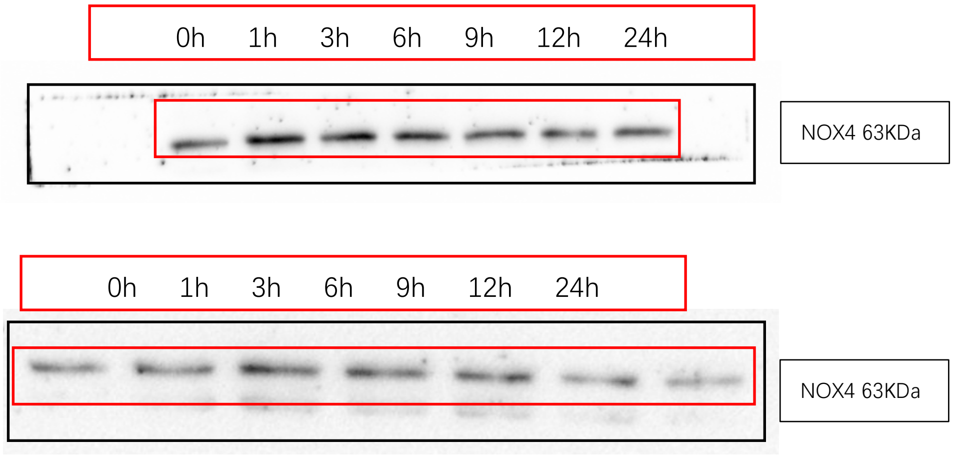
0h 1h 3h 6h 9h 12h 24h
NOX4 63KDa
0h 1h 3h 6h 9h 12h 24h
NOX4 63KDa

Fig 6E

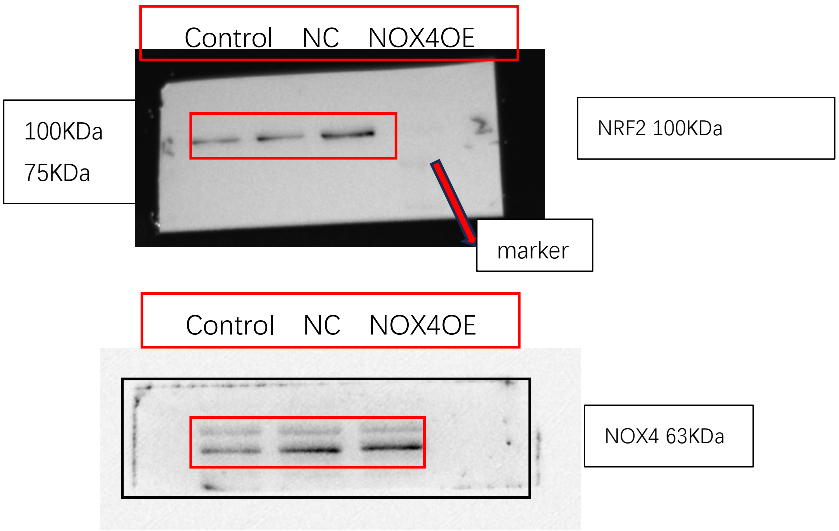
Control NC NOX4OE
100KDa
75KDa
NRF2 100KDa
marker
Control NC NOX4OE
NOX4 63KDa

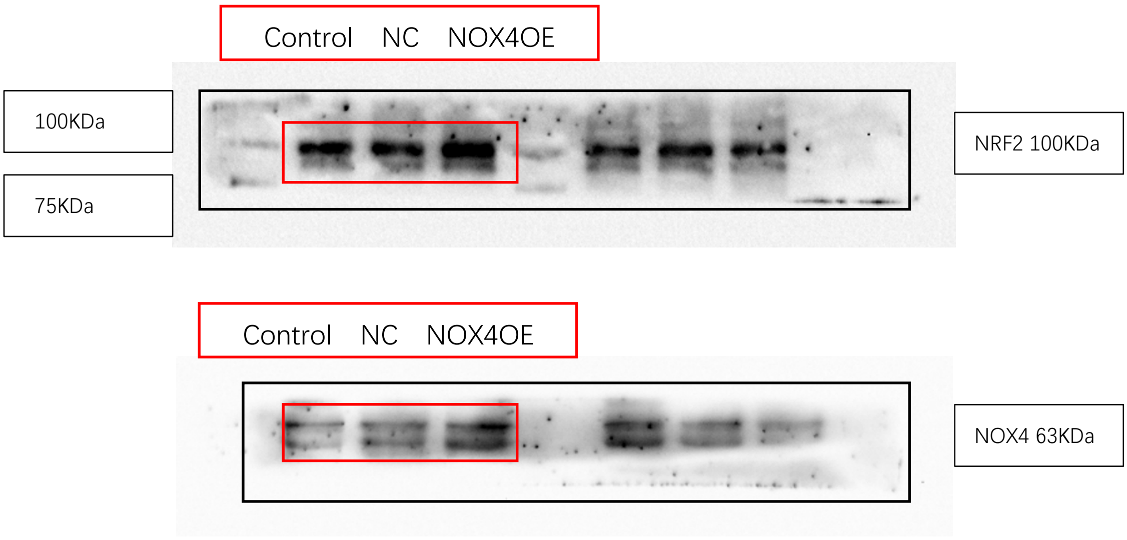

Fig 6G

multiple exposure images of NOX4

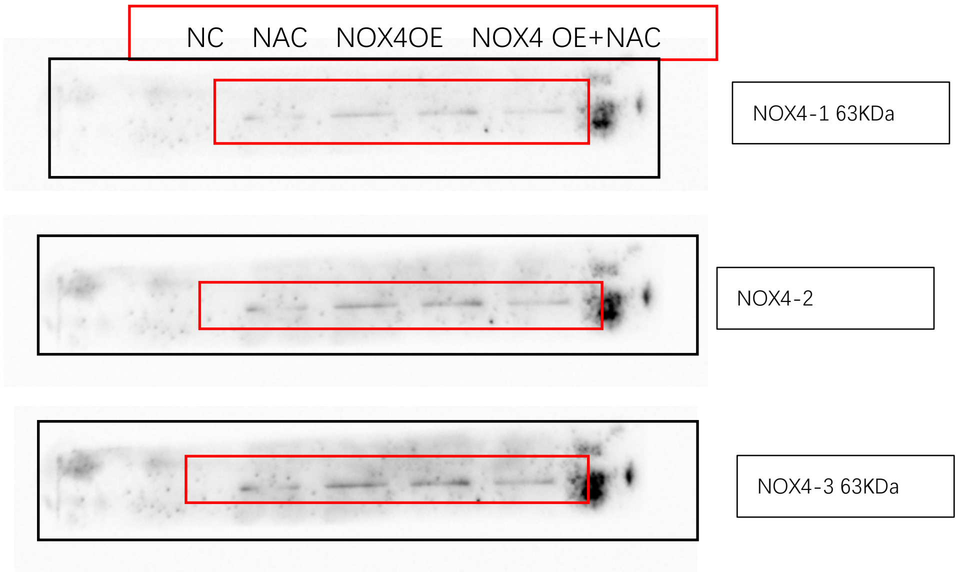

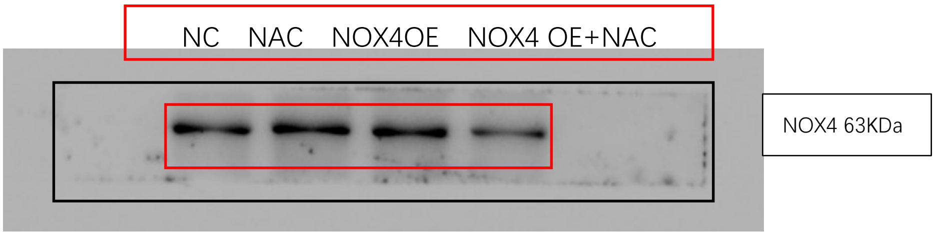

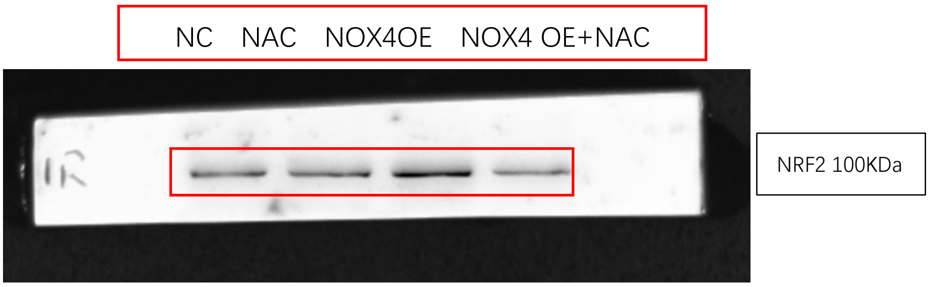
NC NAC NOX4OE NOX4 OE+NAC
NRF2 100KDa

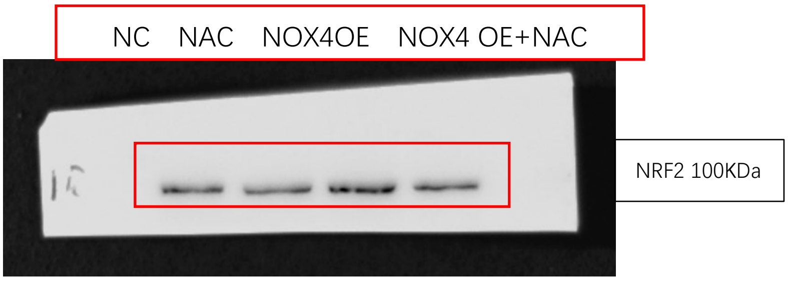
NC NAC NOX4OE NOX4 OE+NAC
NRF2 100KDa

Fig 6H

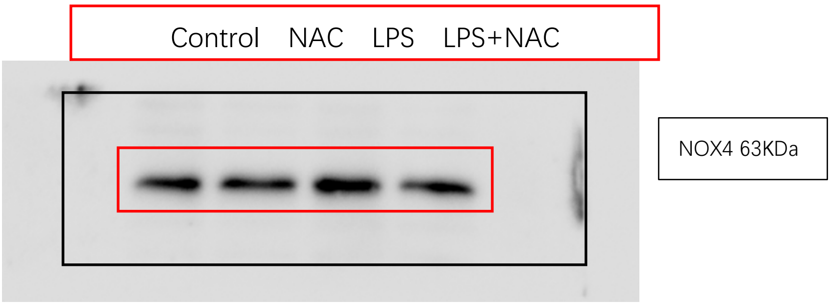
Control NAC LPS LPS+NAC
NOX4 63KDa

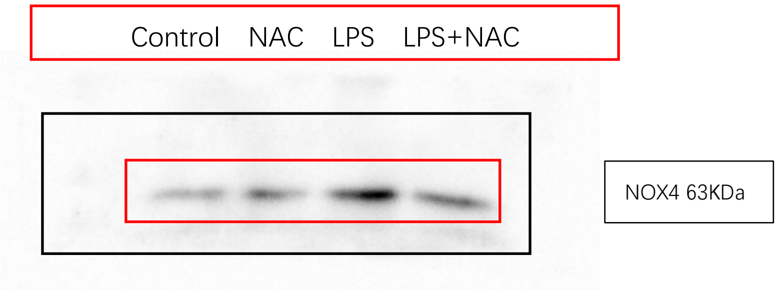
Control NAC LPS LPS+NAC
NOX4 63KDa

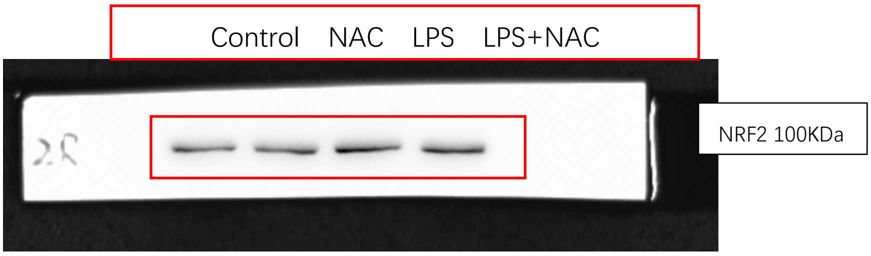
Control NAC LPS LPS+NAC
NRF2 100KDa

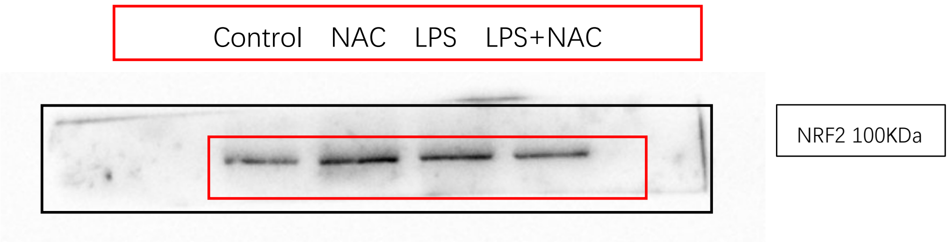
Control NAC LPS LPS+NAC
NRF2 100KDa
